# Synthesis and Cytotoxic Analysis of Novel Myrtenyl Grafted Pseudo-Peptides Revealed Potential Candidates for Anticancer Therapy

**DOI:** 10.3390/molecules25081911

**Published:** 2020-04-21

**Authors:** Odette Concepción, Julio Belmar, Alexander F. de la Torre, Francisco M. Muñiz, Mariano W. Pertino, Barbara Alarcón, Valeska Ormazabal, Estefania Nova-Lamperti, Felipe A. Zúñiga, Claudio A. Jiménez

**Affiliations:** 1 Department of Organic Chemistry, Faculty of Chemical Sciences, Universidad de Concepción, Edmundo Larenas 129, Concepción P.C. 4070371, Chile; jbelmar@udec.cl (J.B.); afernandezd@udec.cl (A.F.d.l.T.); fmunozm@udec.cl (F.M.M.); 2 Institute of Natural Resources Chemistry, Universidad de Talca, Casilla 747, Avenida Lircay, Talca P.C. 3462227, Chile; mwalter@utalca.cl; 3 Department of Clinical Biochemistry and Immunology, Faculty of Pharmacy, Universidad de Concepción, Concepción P.C. 4070371, Chile; balarconz@udec.cl (B.A.); enova@udec.cl (E.N.-L.); fzuniga@udec.cl (F.A.Z.); 4 Department of Pharmacology, Faculty of Biological Sciences, Universidad de Concepción, Concepción P.C. 4070371, Chile; vormazabal@udec.cl

**Keywords:** cytotoxicity, cancer, Myrtenyl derivatives, multicomponent reactions

## Abstract

Myrtenal is a natural monoterpene isolated from essential oils of several plants and their derivates have shown to have several biological properties including cytotoxicity. The cytotoxic activity of these derivates are being investigated for their antitumor effect leading to the development of potential anticancer agents. In this study, novels Myrtenyl grafted pseudo-peptides were designed, synthesized and functionally characterized as possible therapeutic agents for cancer treatment. Thirteen novel Myrtenyl grafted pseudo-peptides were prepared in high atom economy and efficiency by a classic Ugi-4CR and sequential post-modification. Their structures were confirmed by NMR, and ESI-MS, and its cytotoxic activity was evaluated in three cancer cell lines and primary CD4+ T cells at different proliferative cycles. Our results revealed that some of these compounds showed significant cytotoxicity against human gastric, breast and colon adenocarcinoma cells lines, but not against human dermal fibroblast cell line. Moreover, from the thirteen novel myrtenyl synthesized the compound (1*R*,5*S*)-*N*-{[1-(3-chlorophenyl)-1*H*-1,2,3-triazol-4-yl]methyl}-*N*-[2-(cyclohexylamino)-2–oxoethyl]-6,6-dimethylbicyclo[3.1.1]hept-2-ene-2-carboxamide (**3b**) proved to be the best candidate in terms of acceptable EC_50_, and E_max_ values in cancer cell lines and at inducing cytotoxicity in CD4+ T cells undergoing active proliferation, without affecting non-proliferating T cells. Overall, the synthesis and characterization of our Myrtenyl derivates revealed novel potential anticancer candidates with selective cytotoxic activity.

## 1. Introduction

Cancer is one of the leading causes of human death and is estimated to likely rise to 13 million worldwide per year by 2030 [1]. During the last 50 years, anticancer drugs have been developed based on the fact that cancer cells have high proliferation rates relative to healthy cells from the corresponding tissues. In this sense, the DNA molecule is one of the most therapeutic target used [2]. Currently, developing new drugs has evolved towards the design of specific compounds. New drugs not only should be compared by the drug’s potency or IC_50_ (the half-maximum inhibitory concentration), but also by the maximum effect or E_max_ of the drug that is likely to be more informative when the aim is to understand variability in patient responses [3]. Myrtenal is a natural monoterpene isolated from essential oils of several plants, e.g., *Chamaecyparis formosensis*, *Artemisia douglasiana*, *Ferula hermonis*, among others [4,5,6]. This monoterpene can be conveniently prepared [7,8] and exciting and versatile applications have been found such as chiral auxiliaries [9,10,11,12], antimicrobial [5] and antimalarial agents [13], acetylcholinesterase inhibitors [14], anti-inflammatory effect [15], antitumor activities [16,17,18,19,20], and antidiabetic and hepatoprotective effects [21,22].

**Figure 1:**
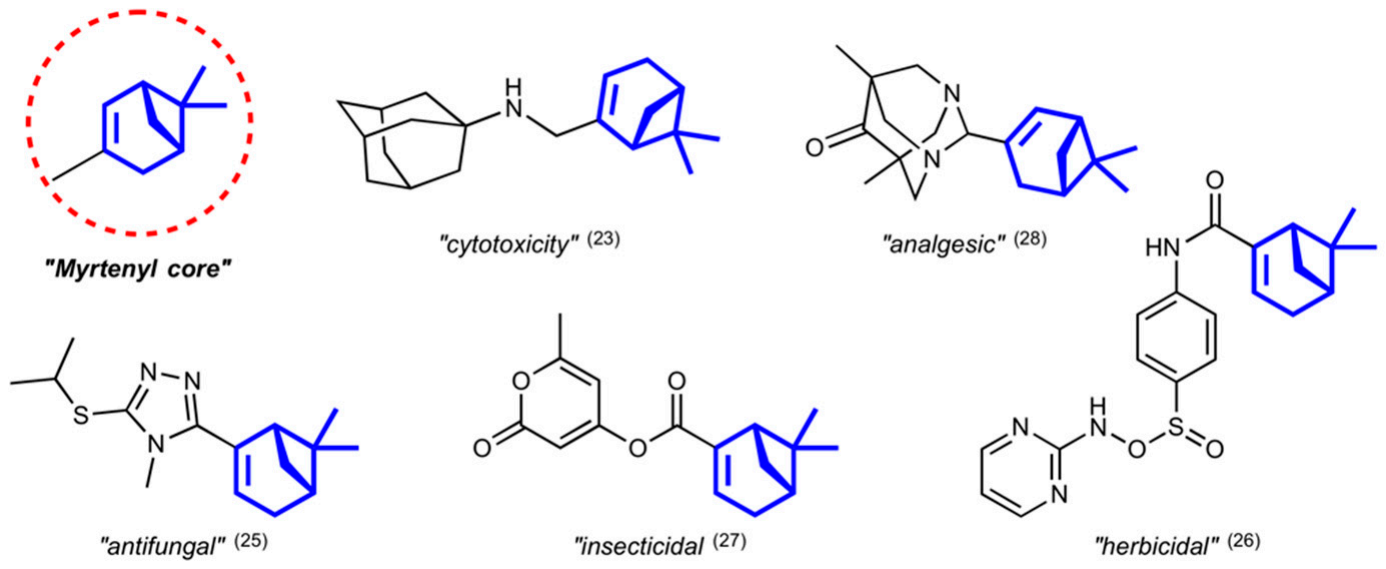
Relevant bioactive Myrtenal hybrid compounds and structure of Myrtenyl core.

Despite the diversified biological activities described for Myrtenal and their derivatives, there is a growing search for novel bioactive compounds, mainly due to their poor stability when used as therapeutic drugs [23]. To this date, there are just a few Myrtenal hybrids described (Figure 1), and aminoadamantane moiety derivatives possess a potent cytotoxic and mutagenic effects [23]. Wang et al. used Myrtenal to obtain analogues of two natural antibiotics (i.e., platensimycin and platencina) with promising antibacterial activity [24]. Additionally, the synthesis of novel 4–methyl–1,2,4–triazole–thioethers [25] and *N-*(4-*N*′-substitutedsulfamoyl) phenyl)myrtenamides [26] with potential antifungal and herbicidal activities were reported. Some Myrtenal-based ester derivatives are proven to have great insecticidal activity [27]. In 2015, novel Myrtenal hybrids were successfully tested as potent analgesic [28]. Nonetheless, none report the synthesis pseudo-peptides decorated with Myrtenyl core for the evaluation of the cytotoxic activity. In view of this, we describe the use of Ugi-4CR (Ugi four-component reaction) with some post-modification reactions, to obtain novel and structurally complex Myrtenyl grafted pseudo-peptides as possible therapeutic agents in cancer treatment.

Pseudo-peptides can be obtained either by solid-phase peptides synthesis (SPPS) or by multicomponent reactions. The Ugi reaction to produce peptoids has many advantages over classic SPPS, such as; they are easy to handle, environmentally friendly, highly diverse scaffold, high atom economy, simple late-stage modifications, and others. In literature, several reports are using the Ugi-4CR for the preparation of scaffold’s peptoids to be successfully evaluated against cancer (see some cited references). Thus, having in mind that peptoids are effective against cancer, and also the monoterpene Myrtenal we assumed that creating pseudo-peptides with embedded myrtenal core will improve their biological activity. Furthermore, the Myrtenyl was used as oxo and acid component in the Ugi reaction to obtain different chemical structures that it is not possible by classic SPPS [29,30]. On the other hand, peptides meet several obstacles in their early development, including proteolytic susceptibility, short ‘in vivo’ half-life (i.e., down in minutes), solubility issues, rapid renal clearance, and poor bioavailability. Thus, based on the structure of peptides, a rational design being efficiently employed to obtain improved candidates with fewer such obstacles. Some structural motifs could improve their therapeutic properties (i.e., pseudo-peptides).

## 2. Results

### 2.1. Synthesis of Myrtenyl Derivativess

**Scheme 1:**
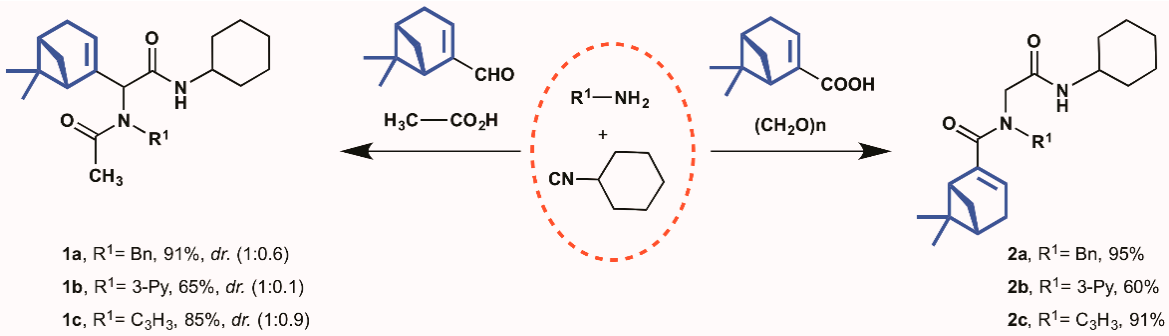
Synthesis of Myrtenyl grafted pseudo-peptides **1a**–**1c** and **2a**–**2c**.

**Scheme 2:**
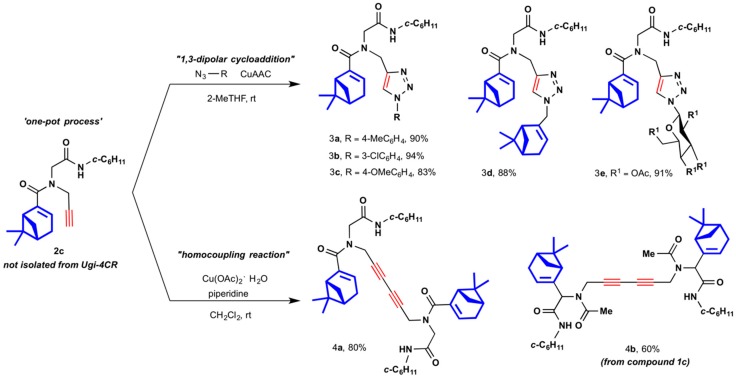
Synthetic post-modification of **2c**, by 1,3-dipolar cycloaddition to obtain **3a**–**3c**, and homocoupling reaction for **4a** and **4b** (was obtained from **1c**).

The well-known Ugi four-component reaction [31,32] (Ugi-4CR), which comprises an acid, an amine, an isocyanide, and carbonyl compound, is further employed for a diverse-oriented synthesis with an application for developing potential drugs [33,34,35,36,37]. Based on the advantages known for the Ugi-4CR [38], we selected this reaction for the one–pot assembly of Myrtenyl derivatives **1a**–**c** and **2a**–**c** (Scheme 1). The standard multicomponent reaction conditions comprise the use of 1.0 equiv. of each reagent, 2-methyltetrahydrofurane (2-MeTHF) as the suitable green solvent, and room temperature. As shown in Scheme 1, the cyclohexyl isocyanide was fixed for all Myrtenyl derivatives obtained. For compounds **1a**–**1c**, Myrtenal and acetic acid were employed in combination with three different amine-components. When either benzylamine or propargylamine was applied, good yield was obtained for **1a** and **1c**, respectively. However, poor diastereoselectivity can be seen by NMR, where the singlet signal of CH_2_ benzyl group has been selected to determine the diastereomeric ratio (see Figures S4 and S11). This poor stereoselectivity is not unexpected because the Ugi-reaction mechanism describes the lack of selectivity when adding isocyanide to the imine. However, even when the lower reactivity of the 3–pyridylamine causes a significant reduction in the yield, it is possible to observe almost exclusively a single diastereomer of the crude and pure compound **1b** (Figure S9) by NMR analysis. The pyridyl group determines the steric or electronic control in the mechanism, allowing a stereoselective approach of isocyanide to the imine. On the other hand, compounds **2a**–**2c** were obtained by Ugi-4CR with the same amines and the isocyanide derivative. The use of Myrtenoic acid and paraformaldehyde as both the acid and oxo-components aimed to evaluate if the position of Myrtenyl moiety performs a fundamental role in the biological activity when compared with compounds **1a**–**1c**. Once again, it is possible to observe a reduced reactivity of 3-pyridylamine, which gave a moderate yield (60%) as reported for **2b**. Heterocycles containing the core 1,2,3-triazole are known to be chemically stable as well as stable in metabolic degradation, which is vital to consider when a new drug is in development. In addition, it is well known that compounds with this heterocycle exhibit a broad spectrum of biological properties and are easy to prepare from available raw materials [39,40,41]. Considering the previous information, the pseudo-peptide **2c** with a terminal alkyne moiety, was synthetically modified by copper-catalyzed 1,3-dipolar cycloaddition (CuAAC) via one-pot methodology and provided compounds **3a**–**3e** in good yields, i.e., 83%–94% (Scheme 2). Satisfyingly, commercially available aryl azides gave the expected 1,4–products. The compounds **3a** and **3b** were obtained in yields slightly above 90%; however, for compound **3c** there was a small drop in yield (83%). The lower reactivity of the inner nitrogen in the azide group could be responsible for this situation when it is compared to the methyl and methoxy derivatives.

The favorable results with the cycloaddition reaction can be extended to include the rapid generation of more complex structures, for example, the second unit of Myrtenyl (**3d**) or a D-glucopyranosyl unit (**3e**). We expect that the incorporation of these biologically related moieties into the skeleton of pseudo-peptides could increase their potential in biological activity. It is necessary to mention that the Myrtenyl azide is not commercially available, and it was prepared by the reaction of (1*R*)-(−)-myrtenol with sodium azide and Ph_3_P in a mixture of CCl_4_–DMF (1:4). This azide derivative was obtained as a light-yellow liquid in 70% yield. Following with the idea of incorporating two Myrtenyl units into the skeleton of the compounds, we designed two novel dimeric compounds. As depicted in Scheme 2, based on a sequential homocoupling reaction, it was possible to synthesize two new compounds having 1,3-dyine moiety. Compounds **4a** and **4b** were obtained from the respective **2c** and **1c** monodyine, in good yield (80 and 60%, respectively). Both dimeric compounds differ mainly in the position of Myrtenyl unit, as shown in Scheme 2.

### 2.2. Biological Assessment

#### 2.2.1. Preliminary Testing

**Figure 2:**
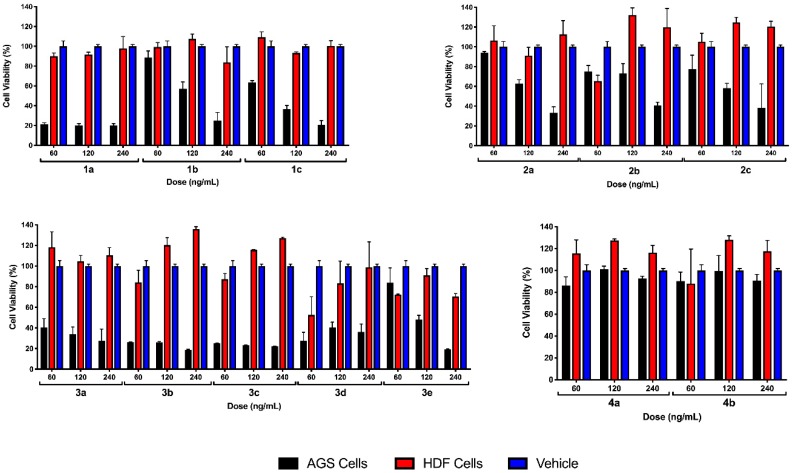
Comparison of human gastric adenocarcinoma (AGS) and human dermal fibroblast (HDF) cells lines viability (%) against Myrtenyl derivates by Sulforhodamine B assay. Three different concentrations of Myrtenyl derivates were analyzed after 24 h of drug treatment in AGS and HFD cells, and cell cytotoxicity was determined by Sulforhodamine B assay. Vehicle: 1% Methanol. The data represent mean ± standard deviation of triplicated determinations in three independent experiments.

The cytotoxic activity of the thirteen myrtenyl derivatives was first screened by using three different concentrations (at 60, 120, and 240 ng/mL) in human cancer gastric adenocarcinoma cell line (AGS), and human dermal fibroblast (HDF) as non-cancer cells after 24 h of incubation (Figure 2). Our preliminary results showed that Myrtenyl derivatives **4a,b** were not cytotoxic to AGS cells, whereas all Myrtenyl derivatives **1a**–**c**, **2a**–**c** and **3a**–**e** were cytotoxic to AGS cells, without affecting HDF. Moreover, Myrtenyl derivatives **1a**, **3a**, **3b**, **3c**, and **3d** showed high cytotoxicity against AGS cells at the lowest concentration (60 ng/mL) without affecting HDF viability, thus the latter five Myrtenyl derivatives were selected for further experiments. A structural analysis in this screening assay showed that extended molecules like **4a** and **4b** may have difficulties entering cells; this is inferred from the activities of the synthetic precursors (**2c**). On the other hand, even when **1a**–**c** (except **1a**) and **2a**–**c** derivatives shown cell cytotoxicity, at comparable concentration **1a**–**c** derivatives have a better performance (**1c**, 40% and **2c**, 60% at 240 ng/mL, Figure 2). Finally, the incorporation of the 1,2,3-triazole core, seems to be the most successful modification, as all **3a**–**e** Myrtenyl derivatives show significant cytotoxicity at 60 ng/mL [42], except **3e** where the incorporation of the D-glucopyranosyl unit seems to be responsible for the low activity.

#### 2.2.2. EC_50_ Determination on Myrtenyl Derivatives

**Figure 3:**
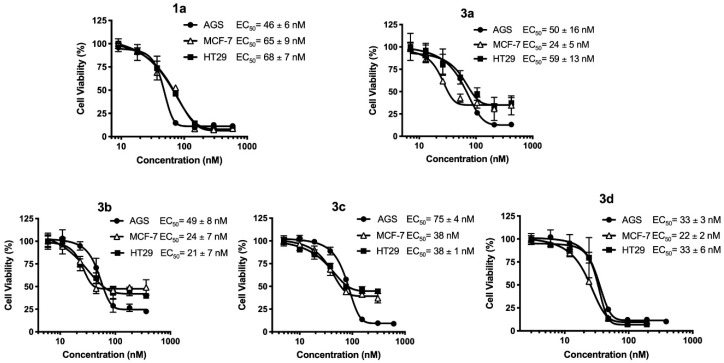
EC_50_ determination of Myrtenyl derivates. Dose-response curves and corresponding EC_50_ values of Myrtenyl derivates were determined after 24 h of drug treatment in AGS, MCF-7, and HT-29 cells lines by Sulforhodamine B assay. Viability is expressed as % compared to control, and data represent mean ± standard deviation of triplicated determinations in three independent experiments.

After selecting the Myrtenyl derivatives with the highest therapeutic potential, we determined their EC_50_ value (i.e., the concentration of a drug that gives half-maximal response) for AGS cells and two additional human cancer cell lines, Caucasian breast adenocarcinoma (MCF-7) and Caucasian colon adenocarcinoma (HT-29) line cells. Our results showed an EC_50_ range between 21 to 75 nM that varies according to the cancer cell line tested and the Myrtenyl derivative used (Figure 3). We also found different E_max_ (or efficacy, i.e., the maximal drug effect on viability rate) values that also depends on the Myrtenyl derivatives used and the cancer cell tested. E_max_ varies between 1 at low drug doses and 0 at high drug doses, which corresponds to the death of all cells.

When we analyze the Myrtenyl derivatives according to EC_50_ and E_max_ values, we observed in detail the consequences of the incorporation of triazole ring in the structure and how the substitution pattern also can be related to the biological activity. First, the integration of triazole ring with electron-donating groups (**3a** and **3c**) showed an EC_50_ value as low as 24 nM, however, the most are around 50 nM and higher. On the other hand, the same compounds present E_max_ values close to 0.1 only for AGS cell lines, where the EC_50_ are not the lower. For the remaining cell lines, the E_max_ values are close to 0.5. When these values are compared with **3b**, where the triazole ring presents an electron-withdrawing group the EC_50_ values fall, in particular for MCF-7 (24 ± 7 nM) and HT-29 (21 ± 7 nM) cells, however, E_max_ values move only slightly to 0.4. These results are supported by the bibliographic information where the 1,2,3-triazoles are important five-membered heterocyclic scaffold strongly associated with many biological activities [42]. On the other hand, over time, it has been found empirically that the introduction of a chlorine atom into one or more specific positions of a biologically active molecule may substantially improve the intrinsic biological activity [43].

Compounds **1a** and **3d** showed the best performance in relation to E_max_ values; all of them are close to 0.1 for each cancer cell lines tested. It draws attention to the structural simplicity of **1a** and its excellent results, but only the Myrtenyl derivative **3d** achieved lower EC_50_ values as well (22 to 33 nM), together with E_max_ values close to 0. The incorporation of two units of Myrtenyl in the compounds **3d** seems to be closely connected with a significant impact in the biological activity.

#### 2.2.3. Viability and Cell Count Analysis of Myrtenyl Derivatives on CD4+ T Lymphocytes in Progressive Proliferative Cycles

**Figure 4:**
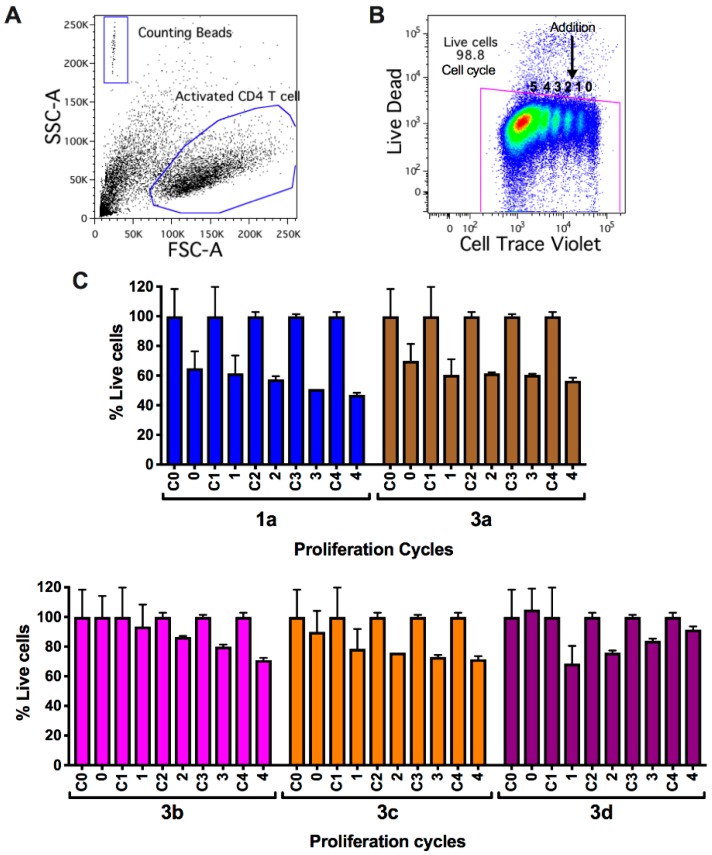
Viability and cell count analysis of Myrtenyl derivatives on CD4+ T lymphocytes in progressive proliferative cycles. (**A**) Representative dot plot of activated CD4+ T cells and counting beads after 6 days of culture. (**B**) Representative dot plot of cell cycles after activation of live CD4+ T cells (2 × 10^5^) CellTrace Violet+ with CD3/CD28 Dynabeads and IL–2 (50 U/mL) in X–VIVO for 6 days. All compounds were added (EC_25_) after 48 h post activation (Addition arrow) and incubated for four days. (**C**) Total number of live cells were counted with flow cytometry, using counting beads as a reference and a Live Dead dye. Viability is expressed as % compared to control, and data represent mean ± standard deviation of triplicated determinations in three independent experiments.

Having shown that Myrtenyl derivatives reduced viability preferentially in cells with high proliferation rates, without affecting low proliferation rates, we evaluated whether Myrtenyl derivatives selectively reduce viability of primary CD4+ T lymphocytes at different proliferation stages using flow cytometry. CD4+ T cells were first stained with a cell tracer dye, a fluorescent reagent that diffuses into cells and binds covalently to intracellular amines, and then with a Live/Dead dye that permeates into cells with damaged membranes, while live cells remain unstained. In addition, counting beads were added into each sample to calculate the exact number of cells per cell cycle (Figure 4A). Then, in order to induce proliferation, fluorescent-labelled CD4+ T cells were activated with anti-CD3/CD28 beads, which mimic physiological T cell receptor triggering and co-stimulation in T lymphocytes. After activation, a proportion of cells proliferated, while some stayed in a non-proliferative state. For proliferating cells, each divided cell showed half of the fluorescence of its precursor, thus after 6 days of activation, we observed five populations of CD4+ T cells in five different proliferation cycles (Figure 4B). Myrtenyl derivatives were added after the second day of culture to evaluate their effect on non-proliferating CD4+ T cells at cycle 0 and 1 and in proliferating cells in cycle 2, 3, and 4. Live CD4+ T cells activated in the presence of five Myrtenyl derivatives were counted and results were normalized with the control sample per cell cycle. Our data show that Myrtenyl derivatives **1a**, **3a**, and **3c** reduced viability of both non-proliferating and proliferating T cells. However, the Myrtenyl derivatives **1a** and **3a** were more cytotoxic than **3c** in non-proliferating cells at cycle 0 (Figure 4C). On the other hand, Myrtenyl derivative **3d** did not reduced viability of non-proliferating cells at cycle 0, however it was the derivative with the lowest capacity to reduce viability in proliferating cells at cycle 4. Finally, Myrtenyl derivative **3b** did not reduce viability of non-proliferating cells at cycle 0 and 1, but it was able to reduce number of proliferating cells at cycle 2, 3, and 4 (Figure 4C). Therefore, **3b** was the most effective derivative at reducing viability of proliferating cells without affecting non-proliferating cells.

## 3. Discussion

In summary, the successful execution of a series of steps such as synthesis, characterization, and evaluation produce thirteen novel Myrtenyl derivatives with promising cytotoxicity in proliferative cells. The multicomponent assembly allows constructing novel pseudo-peptide hybrid compounds with a Myrtenyl moiety in their structure, which together with 1,3–dipolar cycloaddition or homocoupling reaction with terminal alkynes achieve the incorporation of heterocycle or dyine subunits, with very good yields. Notoriously, among all the compounds, **1a**, **3a**, **3b**, **3c**, and **3d** exhibited high cytotoxicity against gastric adenocarcinoma (AGS), Caucasian breast adenocarcinoma (MCF-7), and Caucasian colon adenocarcinoma (HT-29) line cells without affecting human dermal fibroblast (HDF) viability, where the details discussion between EC_50_ and E_max_ values would show some correlation among structure and cell cytotoxicity. In a final evaluation of the cytotoxic effect in primary CD4+ T cells, **3b** proved to be the candidate with the lowest cytotoxicity in the non-proliferative stages, emerging as the most effective derivative at targeting proliferating cells. A comparison of **3b** derivative with another peptomimetic compounds reported [44] shows a better performance in the order of nM concentration, in particular for Caucasian breast adenocarcinoma (MCF-7) and Caucasian colon adenocarcinoma (HT-29) line cells (AGS, 49 ± 8 nM; MCF-7, 24 ± 7 nM and HT-29, 21 ± 7 nM). This study corresponds to the first example where a series of Myrtenal hybrid compounds with complex structures were evaluated, revealing nanomolar cytotoxic activities in 3 different cancer cell lines, showing great potential for the further development of myrtenyl-based pseudo-peptides as anticancer agents.

## 4. Materials and Methods 

### 4.1. Chemistry

#### 4.1.1. General

Myrtenal was purchased from Sigma Aldrich Co. (Darmstadt, Germany). Other reagents were purchased from commercial suppliers and used without further purification. ^1^H NMR and ^13^C NMR spectra were recorded using a Brucker Avance 400 (Bruker Daltonics, Bremen, Germany) 400 MHz for ^1^H and 100 MHz for ^13^C, respectively. Chemical shifts (δ) are reported in parts per million relatives to the residual solvent signals and are given relative to tetramethylsilane (TMS). Coupling constants (*J*) are reported in hertz. Flash column chromatography was carried out using silica gel 60 (230–400 mesh) and analytical thin layer chromatography (TLC) was performed using silica gel aluminum sheets. Compounds were visualized by I_2_, UV, and KMnO_4_. High resolution ESI mass spectra were obtained from an Applied Biosystems QSTAR XL (Foster City/USA) with Fourier transform ion cyclotron resonance (FT–ICR) mass spectrometer, an RF-only hexapole ion guide, and an external electrospray ion source.

#### 4.1.2. General Multicomponent Procedure (**1a**–**2c**)

A suspension of amine (1.0 mmol, 1.0 equiv.) and the oxo-compound (1.0 mmol, 1.0 equiv.) in 2-MeTHF (5 mL) was stirred for 1 h at room temperature. The carboxylic acid (1.0 mmol, 1.0 equiv.) and the isocyanide (1.0 mmol, 1.0 equiv.) were added and the reaction mixture was stirred at room temperature for 24 h. All volatile solvents were removed in a rotary vacuum evaporation system and the crude product was purified by flash column chromatography on silica gel (hexane/EtOAc 1:1).

*2-(N-benzylacetamido)-N-cyclohexyl-2-((1R,5S)-6,6-dimethylbicyclo[3.1.1]hept-2-en-2-yl)acetamide* (**1a**). Yield: 371.5 mg (91%) as an amorphous yellow light solid. *R*_f_ = 0.45 (EtOAc/hexane = 1:3 v/v). A mixture of diastereomers in a 1:0.6 ratio was observed by NMR analysis. ^1^H NMR (CDCl_3_) δ 7.34–7.27 (m, 6H), 7.26–7.22 (m, 4H), 5.78 (d, *J* = 7.9 Hz, 2H), 5.69*, 5.63 (2×s, 1H), 5.18*, 5.04 (2xs, 1H), 4.77, 4.72* (2xd, *J* = 7.4 Hz, 1H), 3.74*, 3.70 (2xm, 1H), 2.33–2.20 (m, 4H), 2.17–2.07 (m, 4H), 2.04, 2.02* (2 x s, 3H), 1.94–1.83 (m, 4H), 1.72–1.55 (m, 8H), 1.24* (s, 3H), 1.38–1.08 (m, 12H), 1.22 (s, 3H), 0.84*, 0.77 (2 x s, 3H). ^13^C NMR (CDCl_3_) δ 173.43*, 172.61, 168.74*, 168.14, 142.29*, 138.07*, 128.63*, 127.19, 126.97*, 126.22*, 124.95*, 65.30, 63.69*, 51.50, 50.32*, 48.56, 48.33*, 45.51*, 44.33, 40.35, 40.11*, 38.04*, 37.98, 36.78*, 33.06*, 32.93, 31.77*, 31.62, 31.49*, 26.13*, 26.11, 25.64*, 24.84*, 22.66*, 22.39, 21.14*, 21.05. (*Correspond to the major diastereomer). HRMS (ESI–FT–ICR) *m/z*: Calcd. for C_26_H_36_N_2_O_2_Na [M + Na]^+^ 431.26745 found 431.26630.

*N-cyclohexyl-2-((1R,5S)-6,6-dimethylbicyclo[3.1.1]hept-2-en-2-yl)-2-(N-(pyridine-3-yl)acetamido)acetamide* (**1b**). Yield: 256.9 mg (65%) as an amorphous yellow light solid. *R*_f_ = 0.15 (EtOAc/hexane = 1:1 v/v). A mixture of diastereomers in a 1:0.1 ratio was observed by NMR analysis. ^1^H NMR (400 MHz, CDCl_3_) δ 8.49 (d, *J* = 4.7 Hz, 2H), 8.13 (d, *J* = 11.2 Hz, 1H), 7.29 (dd, *J* = 8.0, 4.8 Hz, 1H), 5.71 (d, *J* = 7.9 Hz, 1H), 5.50 (s, 1H), 5.21(s, 1H), 3.86–3.77 (m, 1H), 2.07–1.93 (m, 4H), 1.88 (s, 3H), 1.75–1.52 (m, 4H), 1.40–1.29 (m, 2H), 1.19 (s, 3H), 1.26–1.09 (m, 6H), 0.77 (s, 3H). ^13^C NMR (100 MHz, CDCl_3_) δ 171.51, 168.79, 151.02, 148.77, 140.89, 138.08, 137.82, 125.93, 123.56, 66.63, 48.84, 45.92, 39.80, 38.14, 33.18, 33.02, 31.63, 30.80, 26.05, 25.64, 24.90, 24.88, 23.49, 21.07. HRMS (ESI-FT-ICR) *m/z*: Calcd. For C_24_H_33_N_3_O_2_Na [M + Na]^+^ 418.24705 found 418.2459.

*N-cyclohexyl-2-((1R,5S)-6,6-dimethylbicyclo[3.1.1]hept-2-en-2-yl)-2-(N-(prop-2-yn-1-yl)acetamido) acetamide* (**1c**). Yield: 302.8 mg (85%) as an amorphous white solid. *R*_f_ = 0.40 (EtOAc/hexane = 1:3 v/v). A mixture of diastereomers in a 1:0.9 ratio was observed by NMR analysis. ^1^H NMR (CDCl_3_) δ 5.81 (d, *J* = 7.5 Hz, 1H), 5.67*, 5.61 (2xs, 1H), 5.37, 5.27* (2xd, *J* = 1.7 Hz, 1H), 4.25 (dd, *J* = 18.9, 2.4 Hz, 1H), 4.12 (dd, *J* = 19.0, 2.4 Hz, 2H), 3.73 (m, 2H), 2.39 (d, *J* = 5.3 Hz, 2H), 2.28 (s, 3H), 2.27 (s, 3H), 2.23 (t, *J* = 2.4 Hz, 2H), 2.15–2.05 (m, 4H), 1.89 (m, 4H), 1.62 (m, 8H), 1.27*, 1.26 (2xs, 3H), 1.13 (m, 8H), 0.86, 0.84* (2xs, 3H). ^13^C NMR (CDCl_3_) δ 172.54,* 172.23, 168.56,* 168.06, 142.56,* 141.77, 124.61,* 124.33, 80.21,* 72.52, 72.03,* 62.41, 61.81,* 48.55, 48.35,* 44.85,* 44.21, 40.39,* 38.21,* 37.96, 36.77,* 36.57, 35.67,* 33.09,* 32.93, 32.84,* 32.12,* 32.04, 31.85,* 31.65, 28.57, 26.13,* 25.60,* 24.89, 24.81,* 24.78, 24.01, 23.56,* 22.28,* 22.21, 21.13, 21.04*. (* Correspond to the major diastereomer). HRMS (ESI–FT–ICR) *m/z*: Calcd. for C_22_H_32_N_2_O_2_Na [M + Na]^+^ 379.23615 found 379.23580.

*(1R,5S)-N-benzyl-N-[2-(cyclohexylamino)-2-oxoethyl]-6,6-dimethylbicyclo[3.1.1]hept-2-ene-2-carboxamide* (**2a**). Yield: 374.9 mg, (95%) as an amorphous solid. *R*_f_ = 0.35 (EtOAc/hexane = 1:3 v/v). ^1^H NMR (CDCl_3_) δ 7.34 (m, 5H), 6.36 (bs, 1H, NH), 5.92 (bs, 1H), 4.74 (d, *J* = 16.2 Hz, 1H), 4.70 (d, *J* = 16.1 Hz, 1H), 3.97 (d, *J* = 15.7 Hz, 1H), 3.85 (d, *J* = 16.1 Hz, 1H), 3.70 (m, 1H), 2.52–2.42 (m, 2H), 2.37 (t, *J* = 3.0 Hz, 1H), 2.35 (t, *J* = 3.0 Hz, 1H), 2.13 (m, 1H), 1.94–1.49 (m, 6H), 1.31 (s, 3H), 1.42–1.08 (m, 5H), 0.92 (s, 3H). ^13^C NMR (CDCl_3_) δ 172.66, 167.95, 142.85, 136.55, 128.99, 127.89, 126.55, 48.16, 44.59, 40.42, 38.08, 36.76, 32.88, 31.75, 26.00, 25.55, 24.80, 24.76, 21.23. HRMS (ESI–FT–ICR) *m/z*: Calcd. for C_25_H_34_N_2_O_2_Na [M + Na]^+^ 417.25180 found 417.25125.

*(1R,5S)-N-[2-(cyclohexylamino)-2-oxoethyl]-6,6-dimethyl-N-(pyridine-3-yl-methyl)bicyclo[3.1.1]hept-2-ene-2-carboxamide* (**2b**). Yield: 228.9 mg (60%) as an amorphous white solid. *R*_f_ = 0.20 (EtOAc/hexane = 1:1 v/v). ^1^H NMR (CDCl_3_) δ 8.49 (dd, *J* = 4.7, 1.1 Hz, 1H), 8.43 (d, *J* = 2.3 Hz, 1H), 7.65 (ddd, *J* = 8.1, 2.3, 1.5 Hz, 1H), 7.32 (dd, *J* = 8.1, 4.8 Hz, 1H), 6.35 (d, *J* = 7.6 Hz, 1H, NH), 5.83 (m, 1H), 4.32 (s, 2H), 3.76 (m, 1H), 2.27 (m, 1H), 2.22–2.17 (m, 3H), 2.02–1.83 (m, 2H), 1.74–1.65 (m, 2H), 1.63–1.55 (m, 2H), 1.43–1.12 (m, 6H), 1.21 (s, 3H), 0.74 (s, 3H). ^13^C NMR (CDCl_3_) δ: 171.00, 167.58, 148.53, 148.02, 142.72, 140.43, 134.50, 132.69, 123.98, 54.44, 48.32, 44.04, 39.99, 37.92, 32.99, 31.98, 31.11, 25.91, 25.57, 24.72, 20.98. HRMS (ESI–FT–ICR) *m/z*: Calcd. for C_23_H_31_N_3_O_2_H [M + H]^+^ 382.24945 found 382.24890.

*(1R,5S)-N-[2-(cyclohexylamino)-2-oxoethyl]-6,6-dimethyl-N-(prop-2-yn-1-yl)bicyclo[3.1.1]hept-2-ene-2-carboxamide* (**2c**). Yield: 311.7 mg (91%) as an amorphous white solid. *R*_f_ = 0.41 (EtOAc/hexane = 1:3 v/v). ^1^H NMR (CDCl_3_) δ 6.25 (bs, 1H), 6.06 (bs, 1H), 4.28 (dd, *J* = 17.8, 2.2 Hz, 1H), 4.17 (dd, *J* = 17.8, 2.4 Hz, 1H), 4.09 (d, *J* = 15.9 Hz, 1H), 3.98 (d, *J* = 15.9 Hz, 1H), 3.78–3.68 (m, 1H), 2.51–2.32 (m, 4H), 2.12 (m, 1H), 1.84 (m, 2H), 1.71–1.52 (m, 3H), 1.30 (s, 3H), 1.42–1.09 (m, 7H), 0.88 (s, 3H). ^13^C NMR (CDCl_3_) δ 171.76, 167.75, 142.36, 128.11, 78.68, 73.27, 48.27, 44.17, 40.35, 37.96, 36.70, 32.91, 31.84, 31.69, 25.92, 25.54, 24.75, 21.14. HRMS (ESI–FT–ICR) *m/z*: Calcd. for C_21_H_30_N_2_O_2_Na [M + Na]^+^ 365.22050 found 365.21995.

#### 4.1.3. One-Pot Multicomponent-Click General Procedure (**3a**–**3e**)

A suspension of amine (0.1 mmol, 1.0 equiv.) and the oxo-compound (0.1 mmol, 1.0 equiv.) in 2–MeTHF (5 mL) was stirred for 1 h at room temperature. The carboxylic acid (0.1 mmol, 1.0 equiv.) and the isocyanide (0.1 mmol, 1.0 equiv.) were added and the reaction mixture was stirred at room temperature for 24 h. Then, the correspondent azide (1.1 equiv.) was added and the mixture was treated with a solution of CuSO_4_·H_2_O (20 mol%) and with a solution of sodium ascorbate (40 mol%), enabling the formation of Cu(I) in situ. The reaction mixture was stirred for 12 h at room temperature. After the reaction ended, which was checked by TLC, all volatile solvents were removed in a rotary vacuum evaporation system and the crude product was diluted in 20 mL of EtOAc and washed successively with NaHCO_3_ (sat), NaCl solution (sat), and water. The extract was dried over Na_2_SO_4_ and purified by silica gel column chromatography.

*(1R,5S)-N-[2-(cyclohexylamino)-2-oxoethyl]-6,6-dimethyl-N-{[1-(p-tolyl)-1H-1,2,3-triazol-4-yl]methyl} bicyclo[3.1.1]hept-2-ene-2-carboxamide* (**3a**). Yield: 42.8 mg (90%) as an amorphous white solid. *R*_f_ = 0.20 (EtOAc/hexane = 1:1 v/v). ^1^H NMR (MeOD) δ 8.39 (s, 1H), 7.71 (d, *J* = 8.0 Hz, 2H), 7.40 (d, *J* = 8.0 Hz, 2H), 6.08 (m, 1H), 4.75 (bs, 1H), 4.12 (m, 2H), 3.65 (m, 1H), 2.57–2.36 (m, 4H), 2.44 (s, 3H), 2.15 (d, *J* = 7.1 Hz, 1H), 1.79–1.60 (m, 4H), 1.64 (d, *J* = 12.0 Hz, 1H), 1.34 (s, 3H), 1.44–1.14 (m, 7H), 0.93 (s, 3H). ^13^C NMR (MeOD) δ 174.45, 169.68, 140.53, 136.07, 131.36, 121.46, 49.91, 45.50, 41.65, 38.86, 33.68, 32.58, 32.55, 26.59, 26.34, 26.00, 21.55, 21.06. HRMS (ESI–FT–ICR) *m/z*: Calcd. for C_28_H_37_N_5_O_2_H [M + H]^+^ 476.30255 found 476.30200.

*(1R,5S)-N-{[1-(3-chlorophenyl)-1H-1,2,3-triazol-4-yl]methyl}-N-[2-(cyclohexylamino)-2-oxoethyl]-6,6-dimethylbicyclo[3.1.1]hept-2-ene-2-carboxamide* (**3b**). Yield: 46.6 mg (94%) as an amorphous white solid. *R*_f_ = 0.18 (EtOAc/hexane = 1:1 v/v). ^1^H NMR (400 MHz, MeOD) δ 8.50 (bs, 1H), 8.00 (s, 1H), 7.83 (d, *J* = 7.9 Hz, 1H), 7.58 (t, *J* = 8.0 Hz, 1H), 7.52 (d, *J* = 8.0 Hz, 1H), 6.08 (bs, 1H), 4.75 (bs, 2H), 4.31–4.05 (m, 2H), 3.69–3.60 (m, 1H), 2.58–2.34 (m, 5H), 2.15 (d, *J* = 7.0 Hz, 1H), 1.79–1.60 (m, 5H), 1.64 (d, *J* = 12.0 Hz, 1H), 1.34 (s, 3H), 1.42–1.17 (m, 9H), 0.93 (s, 3H). ^13^C NMR (100 MHz, MeOD) δ 174.43, 169.67, 145.85, 144.04, 139.46, 136.60, 132.45, 129.97, 128.65, 123.31, 121.63, 119.80, 49.93, 45.49, 41.71, 38.86, 33.69, 32.79, 26.59, 26.34, 26.01, 21.56. HRMS (ESI-FT-ICR) *m/z*: Calcd. for C_27_H_34_ClN_5_O_2_Na [M + Na]+ 518.22987 found 518.22932.

*(1R,5S)-N-[2-(cyclohexylamino)-2-oxoethyl]-N-{[1-(4-methoxyphenyl)-1H-1,2,3-triazol-4-yl]methyl}-6,6-dimethylbicyclo[3.1.1]hept-2-ene-2-carboxamide* (**3c**). Yield: 40.8 mg (83%) as an amorphous white solid. *R*_f_ = 0.15 (EtOAc/hexane = 1:1 v/v). ^1^H NMR (400 MHz, MeOD) δ 8.33 (s, 1H), 7.73 (d, *J* = 8.2 Hz, 2H), 7.12 (d, *J* = 8.2 Hz, 2H), 6.07 (d, *J* = 7.3 Hz, 1H), 4.74 (bs, 2H), 4.24–4.03 (m, 2H), 3.88 (s, 3H), 3.71–3.59 (m, 1H), 2.59–2.34 (m, 5H), 2.14 (brs, 1H), 1.90–1.56 (m, 7H), 1.31 (s, 3H), 1.37–1.22 (m, 7H), 0.93 (s, 3H). ^13^C NMR (100 MHz, CDCl_3_) δ 172.57, 160.00, 142.60, 130.47, 128.03, 122.28, 114.89, 60.50, 55.74, 44.25, 40.40, 37.99, 32.94, 31.86, 31.70, 31.04, 25.97, 25.56, 24.94, 21.23, 14.31. HRMS (ESI-FT-ICR) *m/z*: Calcd. for C_28_H_37_N_5_O_3_H [M + H]+ 492.29747 found 492.29691.

*(1R,5S)-N-[2-(cyclohexylamino)-2-oxoethyl]-N-({1-[((1R,5S)-6,6-dimethylbicyclo[3.1.1]hept-2-en-2-yl)methyl]-1H-1,2,3-triazol-4-yl}methyl)-6,6-dimethylbicyclo[3.1.1]hept-2-ene-2-carboxamide* (**3d**). Yield: 45.7 mg (88%) as an amorphous white solid. *R*_f_ = 0.25 (EtOAc/hexane = 1:1 v/v). ^1^H NMR (400 MHz, CDCl_3_) δ 7.54 (s, 1H), 6.01 (s, 1H), 5.58 (s, 1H), 4.82 (s, 2H), 4.58 (s, 2H), 4.17–3.83 (m, 4H), 3.82–3.63 (m, 1H), 2.50–2.21 (m, 9H), 2.09 (m, 2H), 1.99 (t, *J* = 5.2 Hz, 2H), 1.92–1.54 (m, 9H), 1.40–1.26 (m, 9H), 1.24 (s, 3H), 1.20 (s, 3H), 1.12–1.01 (m, 6H), 0.87 (s, 3H), 0.70 (s, 3H). ^13^C NMR (100 MHz, CDCl_3_) δ 172.45, 167.88, 143.88, 142.62, 142.02, 127.86, 123.32, 60.51, 59.64, 55.46, 44.21, 43.54, 40.51, 40.40, 38.26, 38.11, 37.97, 32.94, 32.89, 31.83, 31.72, 31.67, 31.35, 29.81, 26.00, 25.97, 25.57, 21.07. HRMS (ESI-FT-ICR) *m/z*: Calcd. for C_31_H_45_N_5_O_2_Na [M + Na]^+^ 542.34710 found 542.346547.

*(2S,3S,4R,5S,6S)-2-(acetoxymethyl)-6-[4-({(1R,5S)-N-[2-(cyclohexylamino)-2-oxoethyl]-6,6-dimethylbicyclo [3.1.1]hept-2-ene-2-carboxamido}methyl)-1H-1,2,3-triazol-1-yl]tetrahydro-2H-pyran-3,4,5-triyl triacetate* (**3e**). Yield: 65 mg (91%) as an amorphous white solid. *R*_f_ = 0.27 (EtOAc/hexane = 1:1 v/v). ^1^H NMR (CDCl_3_) δ 7.83 (s, 1H), 6.05 (s, 1H), 5.84 (d, *J* = 8.3 Hz, 1H), 5.49–5.36 (m, 2H), 5.24 (t, *J* = 9.4 Hz, 1H), 4.68 (2 x bs, 2H), 4.32 (dd, *J* = 12.7, 4.7 Hz, 1H), 4.19–3.99 (m, 4H), 3.79 (bs, 1H), 2.46 (m, 2H), 2.37 (d, *J* = 10.6 Hz, 2H), 2.10 (s, 3H), 2.07 (s, 3H), 2.03 (s, 3H), 1.87 (s, 3H), 1.87 (m, 2H), 1.67 (2 x d, *J* = 11.1 Hz, 4H), 1.31 (s, 3H), 1.43–1.12 (m, 7H), 0.90 (s, 3H). ^13^C NMR (CDCl_3_) δ 172.41, 170.63, 170.02, 169.42, 168.91, 167.70, 144.47, 142.53, 127.85, 121.66, 85.92, 75.30, 72.61, 70.54, 67.75, 61.57, 44.21, 40.38, 37.97, 32.96, 32.92, 31.83, 31.68, 25.96, 25.57, 24.89, 21.21, 21.15, 20.81, 20.63, 20.62, 20.23. HRMS (ESI–FT–ICR) *m/z*: Calcd. for C_35_H_49_N_5_O_11_Na [M + Na]^+^ 738.33263 found 738.33207.

#### 4.1.4. General Multicomponent Sequential Homocoupling Procedure (**4a** and **4b**)

A suspension of amine (0.1 mmol, 1.0 equiv.) and the oxo-compound (0.1 mmol, 1.0 equiv.) in 2-MeTHF (5 mL) was stirred for 1 h at room temperature. The carboxylic acid (0.1 mmol, 1.0 equiv.) and the isocyanide (0.1 mmol, 1.0 equiv.) were added and the reaction mixture was stirred at room temperature for 24 h. The reaction course was monitored by TLC, and additional cycles of 30 min were applied in cases of poor consumption of the starting material. The volatile section was concentrated under reduced pressure and the resulting crude product was dissolved in 100 mL of CH_2_Cl_2_. The organic phase was washed sequentially with an aqueous saturated solution of citric acid (50 mL), aqueous 10% NaHCO_3_ (2 × 50 mL), and brine (50 mL), and then dried over anhydrous Na_2_SO_4_ and concentrated under reduced pressure. The resulting amorphous solid was used in the homocoupling step without further purification. A mixture of alkyne (1.0 mmol, 1.0 equiv.), piperidine (1.0 mmol, 1.0 equiv.), and Cu(OAc)_2_·H_2_O (0.1 mmol, 0.10 equiv., 10 mol%) in CH_2_Cl_2_ (2 mL) was stirred at atmospheric air at 25 °C. After the reaction ended, which was checked by TLC, all volatile solvents were removed in a rotary vacuum evaporation system and the residue was purified by silica gel column chromatography.

*(1R,1′R,5S,5′S)-N,N′-(hexa-2,4-diyne-1,6-diyl)-bis{N-[2-(cyclohexylamino)-2-oxoethyl]-6,6-dimethylbicyclo [3.1.1]hept-2-ene-2-carboxamide}* (**4a**). Yield: 55 mg (80%) as an amorphous white solid. *R*_f_ = 0.35 (EtOAc/hexane = 1:1 v/v). ^1^H NMR (CDCl_3_) δ 6.26 (s, 2H), 6.06 (s, 2H), 4.40 (d, *J* = 18.2 Hz, 2H), 4.30 (d, *J* = 18.2 Hz, 2H), 4.13 (d, *J* = 15.7 Hz, 2H), 3.98 (d, *J* = 15.7 Hz, 2H), 3.76 (m, 2H), 2.60–2.31 (m, 8H), 2.16 (d, *J* = 7.1 Hz, 2H), 1.93–1.79 (m, 6H), 1.73–1.53 (m, 6H), 1.33 (s, 6H), 1.44–1.10 (m, 15H), 0.91 (s, 6H). ^13^C NMR (CDCl_3_) δ 171.63, 167.43, 142.28, 128.44, 73.93, 68.79, 60.49, 48.36, 44.20, 40.36, 37.98, 32.96, 31.89, 31.74, 25.95, 25.56, 24.80, 21.17, 14.29. HRMS (ESI–FT–ICR) *m/z*: Calcd. for C_42_H_58_N_4_O_4_H [M + H]^+^ 683.45363 found 683.45308.

*2,2′-[hexa-2,4-diyne-1,6-diylbis(acetylazanediyl)]-bis[N-cyclohexyl-2-((1R,5S)-6,6-dimethylbicyclo[3.1.1] hept-2-en-2-yl)acetamide]* (**4b**). Yield: 43 mg (60%) as an amorphous white solid. *R*_f_ = 0.40 (EtOAc/hexane = 1:1 v/v). A mixture of diastereomers in a 1:0.9 ratio was observed by NMR analysis. ^1^H NMR (CDCl_3_) δ 5.67 (m, 6H), 5.38 (s, 2H), 5.26 (s, 2H), 4.41 (dd, *J* = 19.3, 3.7 Hz, 2H), 4.27 (d, *J* = 20.2 Hz, 2H), 4.17 (d, *J* = 9.6 Hz, 2H), 3.80–3.68 (m, 4H), 2.45 (m, 4H), 2.29 (m, 4H), 2.26 (s, 6H), 2.25 (s, 6H), 2.15–2.02 (m, 10H), 1.96–1.79 (m, 12H), 1.76–1.57 (m, 12H), 1.29 (s, 6H), 1.28 (s, 6H), 1.42–1.06 (m, 26H), 0.88 (s, 6H), 0.86 (s, 6H). ^13^C NMR (CDCl_3_) δ 172.23, 172.03, 168.50, 168.08, 142.54, 141.69, 124.86, 124.69, 75.25, 75.00, 68.43, 67.94, 61.99, 61.36, 48.62, 48.39, 44.93, 44.14, 40.44, 40.36, 38.21, 37.98, 37.14, 36.25, 33.09, 32.96, 32.85, 32.10, 31.89, 31.68, 31.04, 26.12, 25.59, 24.93, 24.80, 22.24, 21.12, 21.06. HRMS (ESI–FT–ICR) *m/z*: Calcd. for C_44_H_62_N_4_O_4_H [M + H]^+^ 711.48493 found 711.48438.

### 4.2. Biological Activity

#### 4.2.1. Cell Culture

We used the human gastric adenocarcinoma (AGS, ATCC CRL–1 1739), human Caucasian colon adenocarcinoma (HT-29, ECACC 91072201), human Caucasian breast adenocarcinoma (MCF-7, ECACC 86012803) as cancer cells and human dermal fibroblast (HDF, Cell Applications, INC., San Diego, CA, USA, 106–05A) as non-cancer cells. The cell lines were expanded, cultured, and maintained at 37 °C in a humid atmosphere with 5% CO_2_ in modified Dulbecco’s medium (Thermo Fisher Scientific, Waltham, MA, USA), with 10% FBS (Thermo Fisher Scientific, Waltham, MA, USA) and 100 U/mL of the antibiotic mixture penicillin/streptomycin (Thermo Fisher Scientific, Waltham, MA, USA).

#### 4.2.2. Cell Cytotoxicity Assay

We evaluated the cytotoxicity effects of novel Myrtenal hybrids against on cells by the sulforhodamine B (SRB) assay [45]. Initially, the cells were seeded in 96-well microplates at a density of 2 × 10^4^ cells/well. All compounds were initially dissolved in 100% of methanol and prediluted to reduce methanol concentration to 1% or lower in culture medium and added to the cells at different concentrations for 24 h. Then, cells were fixed by adding 10% (w/v) trichloroacetic acid (TCA) (Winkler) in each well and were stained with 0.057% (w/v) Sulforhodamine B (SRB) (Sigma-Aldrich) in 1% (v/v) acetic acid (Winkler). The bound dye was dissolved in 10 mM Tris base (Sigma-Aldrich), and the absorbance was determined at 510 nm using a multiplate reader (Synergy 2, Biotek Instruments). Percentage cell viability was determined using the formula: Percentage cell viability = [(Absorbance of untreated cells − Absorbance of treated cells)/Absorbance of untreated cells] × 100. The absorbance from the wells of the negative control (1% methanol in culture medium) was considered as the 100% viability. EC_50_ values were determined by regression analysis (R^2^ > 0.95) of the corresponding dose response curves of percentage of cell viability and concentration of the compounds. E_max_, the maximum effect of the drug at the highest tested concentration were determined using an online calculator (http://www.grcalculator.org/). E_max_ lies between 1 and 0; a value of 0 corresponds to a fully cytotoxic response.

#### 4.2.3. Cell Cycle and Proliferation Assays

CD4+ T cells were obtained from peripheral blood from healthy volunteers. Blood samples were diluted 1:2 with PBS before adding 50 μL/mL of RosettSep Human CD4+ T cell enrichment cocktail (STEM CELL) for 20 min at room temperature. After incubation, samples were diluted again 1:4 with PBS and CD4+ T cells were obtained directly from the mononuclear fraction obtained after Ficoll–Hypaque density gradient centrifugation. CD4+ T cells were washed twice, counted, and resuspended in PBS. CD4+ T cells were then stained with 1 μM Cell Trace Violet Cell Proliferation Kit (Invitrogen, Thermo Fisher Scientific, Waltham, MA, USA) for 20 min at 37 °C, protected from light. Unbound dye was quenched by adding cold FCS for 5 min at 4 °C. CD4+ T cells were washed two times and resuspended in X–VIVO (Gibco) with IL–2 (50 U/mL, ProLeukin). Cells (2 × 10^5^) were activated for 48 h with CD3/CD28 Dynabeads (Invitrogen) prior to adding the compounds. After 48 h, compounds were added (EC_25_) and incubated for four days. To analyze proliferation and viability, cells were stained with Live/Dead fixable far red dead cell staining kit (Invitrogen) for 30 min at 4 °C and counting beads were added before acquisition. Cells were acquired on an LSRFortessa X20 (BD) within 24 h. Data were analyzed using FlowJo (Tree Star, Inc., Ashland, OR, USA).

#### 4.2.4. Statistical Analysis

Graphs and statistical analysis of the data were performed using GraphPad Prism version 6 (GraphPad Software, La Jolla, CA, USA, www.graphpad.com). Results are represented as the mean ± SD. Statistical differences were analyzed by Student’s t-tests. A *p*-value of less than 0.05 was considered statistically significant.

## Supplementary Materials

The following are available, Synthesis and Spectra data for selected products, Table S1: EC_50_ and Emax values of derivative against different cancer cell lines, Figures S1–S43: ^1^H and ^13^C NMR, HRMS spectra of compounds **1a**–**c**, **2a**–**c**, **3a**–**e** and **4a**–**b**.
