# Supplementary material for: Synthesis and Cytotoxic Analysis of Novel Myrtenyl Grafted Pseudo-Peptides Revealed Potential Candidates for Anticancer Therapy
Source: Molecules. 2020 Apr 21;25(8):1911. doi: 10.3390/molecules25081911 (PMC7221699; doi:10.3390/molecules25081911)
Supplement: Supplementary file 1 [file molecules-25-01911-s001.pdf]

Supporting Information

Article

Synthesis and Cytotoxic Analysis of Novel Myrtenyl Grafted Pseudo-Peptides Revealed Potential Candidates for Anticancer Therapy

Odette Concepción [1,*], Julio Belmar [1], Alexander F. de la Torre [1], Francisco M. Muñiz [1], Mariano W. Pertino [2], Barbara Alarcón [3], Valeska Ormazabal [4], Estefania Nova-Lamperti [3], Felipe A. Zúñiga [3] and Claudio A. Jiménez [1,*]

1 Department of Organic Chemistry, Faculty of Chemical Sciences, Universidad de Concepción, Edmundo Larenas 129, Concepción, P.C. 4070371, Chile; jbelmar@udec.cl (J.B.); afernandezd@udec.cl (A.F.T.); fmunozm@udec.cl (F.M.M.)
2 Institute of Natural Resources Chemistry, Universidad de Talca, Casilla 747, P.C. 3462227, Avenida Lircay, Talca, Chile; mwalter@utalca.cl
3 Department of Clinical Biochemistry and Immunology, Faculty of Pharmacy, Universidad de Concepción, P.C. 4070371, Concepción, Chile; balarconz@udec.cl (B.A.); enova@udec.cl (E.N.-L.); fzuniga@udec.cl (F.A.Z.)
4 Department of Pharmacology, Faculty of Biological Sciences, Universidad de Concepción, P.C. 4070371, Concepción, Chile; vormazabal@udec.cl
* Correspondence: oconcepcion@udec.cl (O.C.); cjimenez@udec.cl (C.A.J.); Tel. +56-41-22042658 (O.C. & C.A.J.)

Table of contents

Table S1. EC_{50} and E_{max} values of derivative against different cancer cell lines. 13
FIGURE S1. 400 MHz ^1H NMR spectra in $CDCl_3$ of **1a** (Crude mixture of diastereomers) 13
FIGURE S2. 100 MHz ^{13}C NMR spectra in $CDCl_3$ of **1a** (Crude mixture of diastereomers) 14
FIGURE S3. HRMS (ESI-FT-ICR) *m/z* spectra of **1a**. 14
FIGURE S4. 400 MHz ^1H NMR spectra in $CDCl_3$ of major diastereomer of **1a**. 15
FIGURE S5. 100 MHz ^{13}C NMR spectra in $CDCl_3$ of major diastereomer of **1a**. 15
FIGURE S6. 400 MHz ^1H NMR spectra in $CDCl_3$ of **1b** (Crude mixture of diastereomers) 16
FIGURE S7. 100 MHz ^{13}C NMR spectra in $CDCl_3$ of **1b** (Crude mixture of diastereomers) 16
FIGURE S8. HRMS (ESI-FT-ICR) *m/z* spectra of **1b**. 17
FIGURE S9. 400 MHz ^1H NMR spectra in $CDCl_3$ of **1c** (Crude mixture of diastereomers) 17
FIGURE S10. 100 MHz ^{13}C NMR spectra in $CDCl_3$ of **1c** (Crude mixture of diastereomers) 18
FIGURE S11. 400 MHz ^1H NMR spectra in $CDCl_3$ of major diastereomer of **1c.** 18
FIGURE S12. 100 MHz ^{13}C NMR spectra in $CDCl_3$ of major diastereomer of **1c.** 19
FIGURE S13. HRMS (ESI-FT-ICR) *m/z* spectra of **1c**. 19
FIGURE S14. 400 MHz ^1H NMR spectra in $CDCl_3$ of **2a**. 20
FIGURE S15. 100 MHz ^{13}C NMR spectra in $CDCl_3$ of **2a**. 20
FIGURE S16. HRMS (ESI-FT-ICR) *m/z* spectra of **2a**. 21
FIGURE S17. 400 MHz ^1H NMR spectra in $CDCl_3$ of **2b**. 21
FIGURE S18. 100 MHz ^{13}C NMR spectra in $CDCl_3$ of **2b**. 22
FIGURE S19. HRMS (ESI-FT-ICR) *m/z* spectra of **2b**. 22
FIGURE S20. 400 MHz ^1H NMR spectra in $CDCl_3$ of **2c**. 23
FIGURE S21. 100 MHz ^{13}C NMR spectra in $CDCl_3$ of **2c**. 23
FIGURE S22. HRMS (ESI-FT-ICR) *m/z* spectra of **2c**. 24
FIGURE S23. 400 MHz ^1H NMR spectra in CD_3OD of **3a**. 24
FIGURE S24. 100 MHz ^{13}C NMR spectra in CD_3OD of **3a**. 25
FIGURE S25. HRMS (ESI-FT-ICR) *m/z* spectra of **3a**. 25
FIGURE S26. 400 MHz ^1H NMR spectra in CD_3OD of **3b**. 26
FIGURE S27. 100 MHz ^{13}C NMR spectra in CD_3OD of **3b**. 26
FIGURE S28. HRMS (ESI-FT-ICR) *m/z* spectra of **3b**. 27
FIGURE S29. 400 MHz ^1H NMR spectra in MeOD of **3c**. 27
FIGURE S30. 100 MHz ^{13}C NMR spectra in $CDCl_3$ of **3c**. 28
FIGURE S31. HRMS (ESI-FT-ICR) *m/z* spectra of **3c**. 28
FIGURE S32. 400 MHz ^1H NMR spectra in $CDCl_3$ of **3d**. 29
FIGURE S33: 100 MHz ^{13}C NMR spectra in $CDCl_3$ of **3d**. 29
FIGURE S34. HRMS (ESI-FT-ICR) *m/z* spectra of **3d**. 30
FIGURE S35. 400 MHz ^1H NMR spectra in $CDCl_3$ of **3e**. 30
FIGURE S36. 100 MHz ^{13}C NMR spectra in $CDCl_3$ of **3e**. 31
FIGURE S37. HRMS (ESI-FT-ICR) *m/z* spectra of **3e**. 31
FIGURE S38. 400 MHz ^1H NMR spectra in $CDCl_3$ of **4a**. 32

FIGURE S39. 100 MHz ^{13}C NMR spectra in $CDCl_3$ of **4a**. 32
FIGURE S40. HRMS (ESI-FT-ICR) *m*/*z* spectra of **4a**. 33
FIGURE S41. 400 MHz ^{1}H NMR spectra in $CDCl_3$ of **4b**. 33
FIGURE S42. 100 MHz ^{13}C NMR spectra in $CDCl_3$ of **4b**........ 34
FIGURE S43. HRMS (ESI-FT-ICR) *m*/*z* spectra of **4b**. 34

Experimental section

Synthesis and Spectra data for selected products

2-(N-benzylacetamido)-N-cyclohexyl-2-((1R,5S)-6,6-dimethylbicyclo[3.1.1]hept-2-en-2-yl)acetamide (**1a**)

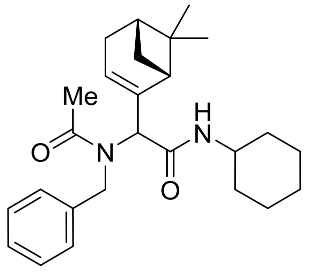

(1*R*)-(−)-Myrtenal (152 μL, 1.0 mmol), benzylamine (109 μL, 1.0 mmol), acetic acid (57 μL, 1.0 mmol) and cyclohexyl isocyanide (124 μL, 1.0 mmol) were reacted according to the general multicomponent procedure. Flash column chromatography purification (EtOAc/hexane = 1:3 v/v) afforded **1a** (371.5 mg, 91%) as an amorphous yellow light solid. R_f = 0.45 (EtOAc/hexane = 1:3 v/v). A mixture of diastereomers in a 1:0.6 ratio was observed by NMR analysis. ^{1}H NMR (400 MHz, $CDCl_3$) δ 7.34–7.27 (m, 6H), 7.26–7.22 (m, 4H), 5.78 (d, *J* = 7.9 Hz, 2H), 5.69* ; 5.63 (2 x s, 1H), 5.18* ; 5.04 (2 x s, 1H), 4.77; 4.72* (2 x d, J = 7.4 Hz, 1H), 3.74* ; 3.70 (2 x m, 1H), 2.33–2.20 (m, 4H), 2.17–2.07 (m, 4H), 2.04, 2.02* (2 x s, 3H), 1.94–1.83 (m, 4H), 1.72–1.55 (m, 8H), 1.24* (s, 3H), 1.38–1.08 (m, 12H), 1.22 (s, 3H), 0.84* (s, 3H), 0.77 (s, 3H). ^{13}C NMR (100 MHz, $CDCl_3$) δ 173.43*, 172.61, 168.74*, 168.14, 142.29*, 138.07*, 128.63*, 127.19, 126.97*, 126.22*, 124.95*, 65.30, 63.69*, 51.50, 50.32*, 48.56, 48.33*, 45.51*, 44.33, 40.35, 40.11*, 38.04*, 37.98, 36.78*, 33.06*, 32.93, 31.77*, 31.62, 31.49*, 26.13*, 26.11, 25.64*, 24.84*, 22.66*, 22.39, 21.14*, 21.05. (*Correspond to the major diastereomer). HRMS (ESI-FT-ICR) *m/z*: Calcd. for $C_{26}H_{36}N_2O_2Na$ $[M + Na]^+$ 431.26745 found 431.26630.

Spectroscopic data of major diastereomer of **1a**:

^{1}H NMR (400 MHz, $CDCl_3$) δ 7.30 (m, 3H), 7.24–7.20 (m, 2H), 5.92 (d, *J* = 7.9 Hz, 1H), 5.69 (s, 1H), 5.21 (d, *J* = 1.1 Hz, 1H), 4.74 (d, *J* = 6.9 Hz, 1H), 3.81–3.70 (m, 1H), 2.24–2.20 (m, 2H), 2.13–2.06 (m, 2H), 2.02 (s, 3H), 1.94–1.86 (m, 2H), 1.72–1.54 (m, 4H), 1.24 (s, 3H), 1.31–1.12 (m, 7H), 0.84 (s, 3H).

^{13}C NMR (100 MHz, $CDCl_3$) δ 173.44, 168.76, 142.27, 138.07, 128.61, 126.94, 126.19, 124.92, 63.60, 50.26, 48.30, 45.54, 40.08, 38.02, 36.77, 33.03, 32.88, 31.74, 31.45, 26.12, 25.63, 24.81, 22.64, 21.12.

N-cyclohexyl-2-((1R,5S)-6,6-dimethylbicyclo[3.1.1]hept-2-en-2-yl)-2-(N-(pyridin-3-yl)acetamido) acetamide **(1b)**

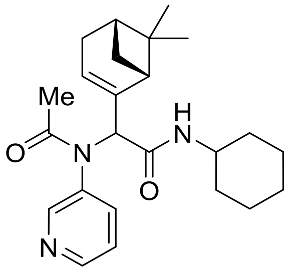

(1*R*)-(−)-Myrtenal (152 μL, 1.0 mmol), pyridin-3-amine (94 mg, 1.0 mmol), acetic acid (57 μL, 1.0 mmol) and cyclohexyl isocyanide (124 μL, 1.0 mmol) were reacted according to the general multicomponent procedure. Flash column chromatography purification (EtOAc/hexane = 1:1 v/v) afforded **1b** (256.9 mg, 65%) as an amorphous yellow light solid. R_f = 0.15 (EtOAc/hexane = 1:1 v/v). A mixture of diastereomers in a 1:0.1 ratio was observed by NMR analysis. ^{1}H NMR (400 MHz, $CDCl_3$) δ 8.49 (d, *J* = 4.7 Hz, 2H), 8.13 (d, *J* = 11.2 Hz, 1H), 7.29 (dd, *J* = 8.0, 4.8 Hz, 1H), 5.71 (d, *J* = 7.9 Hz, 1H), 5.50 (s, 1H), 5.21(s, 1H), 3.86–3.77 (m, 1H), 2.07–1.93 (m, 4H), 1.88 (s, 3H), 1.75–1.52 (m, 4H), 1.40–1.29 (m, 2H), 1.19 (s, 3H), 1.26–1.09 (m, 6H), 0.77 (s, 3H). ^{13}C NMR (100 MHz, $CDCl_3$) δ 171.51, 168.79, 151.02, 148.77, 140.89, 138.08, 137.82, 125.93, 123.56, 66.63, 48.84, 45.92, 39.80, 38.14, 33.18, 33.02, 31.63, 30.80, 26.05, 25.64, 24.90, 24.88, 23.49, 21.07. HRMS (ESI-FT-ICR) *m/z*: Calcd. For $C_{24}H_{33}N_3O_2Na$ [M + Na]$^+$ 418.24705 found 418.2459.

N-cyclohexyl-2-((1R,5S)-6,6-dimethylbicyclo[3.1.1]hept-2-en-2-yl)-2-(N-(prop-2-yn-1-yl)acetamido)acetamide **(1c)**

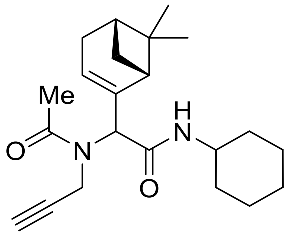

(1*R*)-(-)-Myrtenal (152 μL, 1.0 mmol), propargylamine (64.9 μL, 1.0 mmol), acetic acid (57.2 μL, 1.0 mmol) and cyclohexyl isocyanide (124.2 μL, 1.0 mmol) were reacted according to the general multicomponent procedure. Flash column chromatography purification (EtOAc/hexane = 1:3 v/v) afforded **1c** (302.8 mg, 85%) as an amorphous white solid. R_f = 0.40 (EtOAc/hexane = 1:3 v/v). A mixture of diastereomers in a 1:0.9 ratio was observed by NMR analysis. ^{1}H NMR (400 MHz, $CDCl_3$) δ 5.81 (d, *J* = 7.5 Hz, 1H), 5.67* ; 5.61 (2 x s, 1H), 5.37 ; 5.27* (2 x d, *J* = 1.7 Hz, 1H), 4.25 (dd, *J* = 18.9, 2.4 Hz, 1H), 4.12 (dd, *J* = 19.0, 2.4 Hz, 2H), 3.73 (m, 2H), 2.39 (d, J = 5.3 Hz, 2H), 2.28 (s, 3H), 2.27 (s, 3H), 2.23 (t, J = 2.4 Hz, 2H), 2.15–2.05 (m, 4H), 1.89 (m, 4H), 1.62 (m, 8H), 1.27* , 1.26 (2 x s, 3H), 1.13 (m, 8H), 0.86, 0.84* (2 x s, 3H). ^{13}C NMR ($CDCl_3$) δ 172.54,* 172.23, 168.56,* 168.06, 142.56,* 141.77, 124.61,* 124.33, 80.21,* 72.52, 72.03,* 62.41, 61.81,* 48.55, 48.35,* 44.85,* 44.21, 40.39,* 38.21,* 37.96, 36.77,* 36.57, 35.67,* 33.09,* 32.93, 32.84,* 32.12,* 32.04, 31.85,* 31.65, 28.57, 26.13,* 25.60,* 24.89, 24.81,* 24.78, 24.01, 23.56,* 22.28,* 22.21, 21.13, 21.04*. (*

Correspond to the major diastereomer). HRMS (ESI–FT–ICR) *m/z*: Calcd. for $C_{22}H_{32}N_2O_2Na$ [M + Na]$^+$ 379.23615 found 379.23580.

Spectroscopic data of major diastereomer of **1c**:

^{1}H NMR (400 MHz, $CDCl_3$) δ 5.85 (d, *J* = 7.5 Hz, 1H), 5.67 (s, 1H), 5.26 (s, 1H), 4.25 (dd, *J* = 18.9, 2.4 Hz, 1H), 4.12 (dd, *J* = 18.9, 2.3 Hz, 1H), 3.79–3.65 (m, 1H), 2.40 (m, 1H), 2.27 (s, 3H), 2.23 (t, *J* = 2.4 Hz, 1H), 2.05 (m, 2H), 1.92–1.80 (m, 4H), 1.73–1.53 (m, 3H), 1.36–1.23 (m, 4H), 1.27 (s, 3H), 0.84 (s, 3H). ^{13}C NMR (100 MHz, $CDCl_3$) δ 172.57, 168.58, 142.56, 124.63, 80.21, 72.03, 61.84, 48.36, 44.86, 40.40, 38.22, 36.78, 35.68, 33.09, 32.84, 32.13, 31.86, 26.15, 25.61, 23.64, 22.29, 21.05.

(1R,5S)-N-benzyl-N-(2-(cyclohexylamino)-2-oxoethyl)-6,6-dimethylbicyclo[3.1.1]hept-2-ene-2-carboxamide **(2a)**

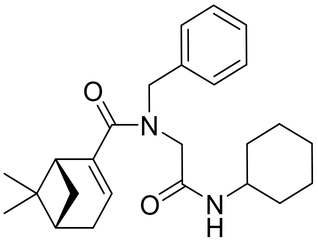

Paraformaldehyde (30.0 mg, 1.0 mmol), benzylamine (109.2 μL, 1.0 mmol), (1R,5S)-6,6-dimethylbicyclo[3.1.1]hept-2-ene-2-carboxylic acid (166.2 mg, 1.0 mmol) and cyclohexyl isocyanide (124.2 μL, 1.0 mmol) were reacted according to the general multicomponent procedure. Flash column chromatography purification (EtOAc/hexane = 1:3 v/v) afforded **2a** (374.9 mg, 95%) as an amorphous solid. R_f = 0.35 (EtOAc/hexane = 1:3 v/v). ^{1}H NMR (400 MHz, $CDCl_3$) δ 7.34 (m, 5H), 6.36 (bs, 1H, NH), 5.92 (bs, 1H), 4.74 (d, *J* = 16.2 Hz, 1H), 4.70 (d, *J* = 16.1 Hz, 1H), 3.97 (d, *J* = 15.7 Hz, 1H), 3.85 (d, *J* = 16.1 Hz, 1H), 3.70 (m, 1H), 2.52–2.42 (m, 2H), 2.37 (t, *J* = 3.0 Hz, 1H), 2.35 (t, *J* = 3.0 Hz, 1H), 2.13 (m, 1H), 1.94–1.49 (m, 6H), 1.31 (s, 3H), 1.42–1.08 (m, 5H), 0.92 (s, 3H). ^{13}C NMR (100 MHz, $CDCl_3$) δ 172.66, 167.95, 142.85, 136.55, 128.99, 127.89, 126.55, 48.16, 44.59, 40.42, 38.08, 36.76, 32.88, 31.75, 26.00, 25.55, 24.80, 24.76, 21.23. HRMS (ESI-FT-ICR) *m/z*: Calcd. for $C_{25}H_{34}N_2O_2Na$ [M + Na]$^+$ 417.25180 found 417.25125.

(1R,5S)-N-(2-(cyclohexylamino)-2-oxoethyl)-6,6-dimethyl-N-(pyridin-3-ylmethyl)bicyclo[3.1.1]hept-2-ene-2-carboxamide **(2b)**

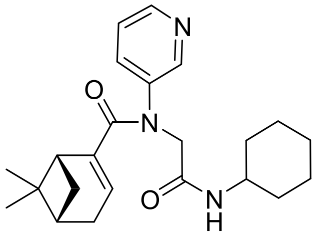

Paraformaldehyde (30.0 mg, 1.0 mmol), pyridin-3-amine (94.0 mg, 1.0 mmol), (1R,5S)-6,6-dimethylbicyclo[3.1.1]hept-2-ene-2-carboxylic acid (166.2 mg, 1.0 mmol) and cyclohexyl isocyanide (124.2 μL, 1.0 mmol) were reacted according to the general multicomponent procedure. Flash column chromatography purification (EtOAc/hexane = 1:1 v/v) afforded **2b** (228.9 mg, 60%) as an amorphous white solid. R_f = 0.20 (EtOAc/hexane = 1:1 v/v). ^{1}H NMR (400 MHz, $CDCl_3$) δ 8.49 (dd, *J* = 4.7, 1.1 Hz, 1H), 8.43 (d, *J* = 2.3 Hz, 1H), 7.65 (ddd, *J* = 8.1, 2.3, 1.5 Hz, 1H), 7.32 (dd, *J* = 8.1, 4.8 Hz, 1H), 6.35 (d, *J* = 7.6 Hz, 1H, NH), 5.83 (m, 1H), 4.32 (s, 2H), 3.76 (m, 1H), 2.27 (m, 1H), 2.22–2.17 (m, 3H), 2.02–1.83 (m, 2H), 1.74–1.65 (m, 2H), 1.63–1.55 (m, 2H), 1.43–1.12 (m, 6H), 1.21 (s, 3H), 0.74 (s, 3H). ^{13}C NMR (100 MHz, $CDCl_3$) δ: 171.00, 167.58, 148.53, 148.02, 142.72, 140.43, 134.50, 132.69, 123.98, 54.44, 48.32, 44.04, 39.99, 37.92, 32.99, 31.98, 31.11, 25.91, 25.57, 24.72, 20.98. HRMS (ESI-FT-ICR) *m/z*: Calcd. for $C_{23}H_{31}N_3O_2H$ [M + H]$^+$ 382.24945 found 382.24890.

(1R,5S)-N-(2-(cyclohexylamino)-2-oxoethyl)-6,6-dimethyl-N-(prop-2-yn-1-yl)bicyclo [3.1.1]hept-2-ene-2-carboxamide **(2c)**

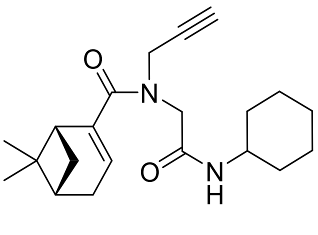

Paraformaldehyde (30.0 mg, 1.0 mmol), propargylamine (64.9 μL, 1.0 mmol), (1R,5S)-6,6-dimethylbicyclo[3.1.1]hept-2-ene-2-carboxylic acid (166.2 mg, 1.0 mmol) and cyclohexyl isocyanide (124.2 μL, 1.0 mmol) were reacted according to the general multicomponent procedure. Flash column chromatography purification (EtOAc/hexane = 1:3 v/v) afforded **2c** (311.7 mg, 91%) as an amorphous white solid. R_f = 0.41 (EtOAc/hexane = 1:3 v/v). ^{1}H NMR (400 MHz, $CDCl_3$) δ 6.25 (bs, 1H), 6.06 (bs, 1H), 4.28 (dd, *J* = 17.8, 2.2 Hz, 1H), 4.17 (dd, *J* = 17.8, 2.4 Hz, 1H), 4.09 (d, *J* = 15.9 Hz, 1H), 3.98 (d, *J* = 15.9 Hz, 1H), 3.78–3.68 (m, 1H), 2.51–2.32 (m, 4H), 2.12 (m, 1H), 1.84 (m, 2H), 1.71–1.52 (m, 3H), 1.30 (s, 3H), 1.42–1.09 (m, 7H), 0.88 (s, 3H).^{13}C NMR (100 MHz, $CDCl_3$) δ 171.76, 167.75, 142.36, 128.11, 78.68, 73.27, 48.27, 44.17, 40.35, 37.96, 36.70, 32.91, 31.84, 31.69, 25.92, 25.54, 24.75, 21.14. HRMS (ESI-FT-ICR) *m/z*: Calcd. for $C_{21}H_{30}N_2O_2Na$ [M + Na]$^+$ 365.22050 found 365.21995.

(1R,5S)-N-(2-(cyclohexylamino)-2-oxoethyl)-6,6-dimethyl-N-((1-(p-tolyl)-1H-1,2,3-triazol-4-yl)methyl)bicyclo[3.1.1]hept-2-ene-2-carboxamide **(3a)**

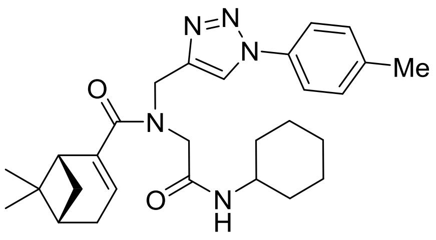

Paraformaldehyde (3.0 mg, 0.1 mmol), propargylamine (6.5 μL, 0.1 mmol), (1R,5S)-6,6-dimethylbicyclo[3.1.1]hept-2-ene-2-carboxylic acid (16.6 mg, 0.1 mmol), cyclohexyl isocyanide (12.4 μL, 0.1 mmol), 4-azidotoluene (14.6 mg, 0.1 mmol), $CuSO_4{\cdot}5H_2O$ (0.02 mmol) and sodium ascorbate (0.04 mmol) were reacted according to the general one-pot multicomponent-click procedure. Flash column chromatography purification (EtOAc/hexane = 1:1 v/v) afforded **3a** (42.8 mg, 90%) as an amorphous white solid. R_f = 0.20 (EtOAc/hexane = 1:1 v/v). ^{1}H NMR (400 MHz, MeOD) δ 8.39 (s, 1H), 7.71 (d, *J* = 8.0 Hz, 2H), 7.40 (d, *J* = 8.0 Hz, 2H), 6.08 (m, 1H), 4.75 (bs, 1H), 4.12 (m, 2H), 3.65 (m, 1H), 2.57–2.36 (m, 4H), 2.44 (s, 3H), 2.15 (d, *J* = 7.1 Hz, 1H), 1.79–1.60 (m, 4H), 1.64 (d, *J* = 12.0 Hz, 1H), 1.34 (s, 3H), 1.44–1.14 (m, 7H), 0.93 (s, 3H). ^{13}C NMR (100 MHz, MeOD) δ 174.45, 169.68, 140.53, 136.07, 131.36, 121.46, 49.91, 45.50, 41.65, 38.86, 33.68, 32.58, 32.55, 26.59, 26.34, 26.00, 21.55, 21.06. HRMS (ESI-FT-ICR) *m/z*: Calcd. for $C_{28}H_{37}N_5O_2H$ $[M + H]^+$ 476.30255 found 476.30200.

(1R,5S)-N-((1-(3-chlorophenyl)-1H-1,2,3-triazol-4-yl)methyl)-N-(2-(cyclohexylamino)-2-oxoethyl)-6,6-dimethylbicyclo[3.1.1]hept-2-ene-2-carboxamide **(3b)**

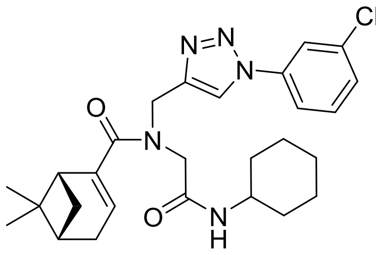

Paraformaldehyde (3.0 mg, 0.1 mmol), propargylamine (6.5 μL, 0.1 mmol), (1R,5S)-6,6-dimethylbicyclo[3.1.1]hept-2-ene-2-carboxylic acid (16.6 mg, 0.1 mmol), cyclohexyl isocyanide (12.4 μL, 0.1 mmol), 1-azido-3-chlorobenzene (16.9 mg, 0.1 mmol), $CuSO_4{\cdot}5H_2O$ (0.02 mmol) and sodium ascorbate (0.04 mmol) were reacted according to the general one-pot multicomponent-click procedure. Flash column chromatography purification (EtOAc/hexane = 1:1 v/v) afforded **3b** (46.6 mg, 94%) as an amorphous white solid. R_f = 0.18 (EtOAc/hexane = 1:1 v/v). ^{1}H NMR (400 MHz, MeOD) δ 8.50 (bs, 1H), 8.00 (s, 1H), 7.83 (d, *J* = 7.9 Hz, 1H), 7.58 (t, *J* = 8.0 Hz, 1H), 7.52 (d, *J* = 8.0 Hz, 1H), 6.08 (bs, 1H), 4.75 (bs, 2H), 4.31–4.05 (m, 2H), 3.69–3.60 (m, 1H), 2.58–2.34 (m, 5H), 2.15 (d, *J* = 7.0 Hz, 1H), 1.79–1.60 (m, 5H), 1.64 (d, *J* = 12.0 Hz, 1H), 1.34 (s, 3H), 1.42–1.17 (m, 9H), 0.93 (s, 3H). ^{13}C NMR (100 MHz, MeOD) δ 174.43, 169.67, 145.85, 144.04, 139.46, 136.60, 132.45, 129.97, 128.65, 123.31, 121.63, 119.80, 49.93, 45.49, 41.71, 38.86, 33.69, 32.79, 26.59, 26.34, 26.01, 21.56. HRMS (ESI-FT-ICR) *m/z*: Calcd. for $C_{27}H_{34}ClN_5O_2Na$ $[M + Na]^+$ 518.22987 found 518.22932.

(1R,5S)-N-(2-(cyclohexylamino)-2-oxoethyl)-N-((1-(4-methoxyphenyl)-1H-1,2,3-triazol-4-yl)methyl)-6,6-dimethylbicyclo[3.1.1]hept-2-ene-2-carboxamide **(3c)**

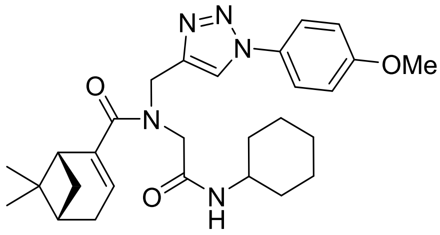

Paraformaldehyde (3.0 mg, 0.1 mmol), propargylamine (6.5 μL, 0.1 mmol), (1R,5S)-6,6-dimethylbicyclo[3.1.1]hept-2-ene-2-carboxylic acid (16.6 mg, 0.1 mmol), cyclohexyl isocyanide (12.4 μL, 0.1 mmol), 4-azidoanisole (16.4 mg, 0.1 mmol), $CuSO_4{\cdot}5H_2O$ (0.02 mmol) and sodium ascorbate (0.04 mmol) were reacted according to the general one-pot multicomponent-click procedure. Flash column chromatography purification (EtOAc/hexane = 1:1 v/v) afforded **3c** (40.8 mg, 83%) as an amorphous white solid. R_f = 0.15 (EtOAc/hexane = 1:1 v/v). ^{1}H NMR (400 MHz, MeOD) δ 8.33 (s, 1H), 7.73 (d, *J* = 8.2 Hz, 2H), 7.12 (d, *J* = 8.2 Hz, 2H), 6.07 (d, *J* = 7.3 Hz, 1H), 4.74 (bs, 2H), 4.24–4.03 (m, 2H), 3.88 (s, 3H), 3.71–3.59 (m, 1H), 2.59–2.34 (m, 5H), 2.14 (brs, 1H), 1.90–1.56 (m, 7H), 1.31 (s, 3H), 1.37–1.22 (m, 7H), 0.93 (s, 3H). ^{13}C NMR (100 MHz, $CDCl_3$) δ 172.57, 160.00, 142.60, 130.47, 128.03, 122.28, 114.89, 60.50, 55.74, 44.25, 40.40, 37.99, 32.94, 31.86, 31.70, 31.04, 25.97, 25.56, 24.94, 21.23, 14.31. HRMS (ESI-FT-ICR) *m/z*: Calcd. for $C_{28}H_{37}N_5O_3H$ [M + H]$^+$ 492.29747 found 492.29691.

(1R,5S)-N-(2-(cyclohexylamino)-2-oxoethyl)-N-((1-(((1R,5S)-6,6-dimethylbicyclo [3.1.1]hept-2-en-2-yl)methyl)-1H-1,2,3-triazol-4-yl)methyl)-6,6-dimethylbicyclo [3.1.1]hept-2-ene-2-carboxamide **(3d)**

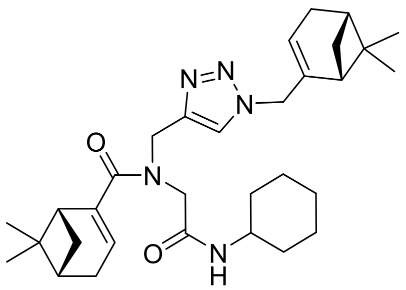

Paraformaldehyde (3.0 mg, 0.1 mmol), propargylamine (6.5 μL, 0.1 mmol), (1R,5S)-6,6-dimethylbicyclo[3.1.1]hept-2-ene-2-carboxylic acid (16.6 mg, 0.1 mmol), cyclohexyl isocyanide (12.4 μL, 0.1 mmol), (1R,5S)-2-(azidomethyl)-6,6-dimethylbicyclo[3.1.1]hept-2-ene [1] (18.0 mg, 0.1 mmol), $CuSO_4{\cdot}5H_2O$ (0.02 mmol) and sodium ascorbate (0.04 mmol) were reacted according to the general one-pot multicomponent-click procedure. Flash column chromatography purification (EtOAc/hexane = 1:1 v/v) afforded **3d** (45.7 mg, 88%) as an amorphous white solid. R_f = 0.25 (EtOAc/hexane = 1:1 v/v). ^{1}H NMR (400 MHz, $CDCl_3$) δ 7.54 (s, 1H), 6.01 (s, 1H), 5.58 (s, 1H), 4.82 (s, 2H), 4.58 (s, 2H), 4.17–3.83 (m, 4H), 3.82–3.63 (m, 1H), 2.50–2.21 (m, 9H), 2.09 (m, 2H), 1.99 (t, J = 5.2 Hz, 2H), 1.92–1.54 (m, 9H), 1.40–1.26 (m, 9H), 1.24 (s, 3H), 1.20 (s, 3H), 1.12–1.01 (m, 6H), 0.87 (s, 3H), 0.70 (s, 3H). ^{13}C NMR (100 MHz, $CDCl_3$) δ 172.45, 167.88, 143.88, 142.62, 142.02, 127.86, 123.32, 60.51, 59.64, 55.46, 44.21, 43.54, 40.51, 40.40, 38.26, 38.11, 37.97, 32.94, 32.89, 31.83, 31.72, 31.67, 31.35, 29.81, 26.00, 25.97, 25.57, 21.07. HRMS (ESI-FT-ICR) m/z: Calcd. for $C_{31}H_{45}N_5O_2Na$ $[M + Na]^+$ 542.34710 found 542.346547.

(2S,3S,4R,5S,6S)-2-(acetoxymethyl)-6-(4-(((1R,5S)-N-(2-(cyclohexylamino)-2-oxoethyl)-6,6-dimethylbicyclo[3.1.1]hept-2-ene-2-carboxamido)methyl)-1H-1,2,3-triazol-1-yl)tetrahydro-2H-pyran-3,4,5-triyl triacetate **(3e)**

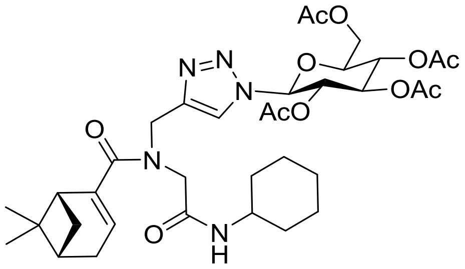

Paraformaldehyde (3.0 mg, 0.1 mmol), propargylamine (6.5 μL, 0.1 mmol), (1R,5S)-6,6-dimethylbicyclo[3.1.1]hept-2-ene-2-carboxylic acid (16.6 mg, 0.1 mmol), cyclohexyl isocyanide (12.4 μL, 0.1 mmol), 2,3,4,6-tetra-O-acetyl-β-D-glucopyranosyl azide[2] (41.1 mg, 0.1 mmol), $CuSO_4{\cdot}5H_2O$ (0.02 mmol) and sodium ascorbate (0.04 mmol) were reacted according to the general one-pot multicomponent-click procedure. Flash column chromatography purification (EtOAc/hexane = 1:1 v/v) afforded **3e** (65 mg, 91%) as an amorphous white solid. R_f = 0.27

(EtOAc/hexane = 1:1 v/v). ^{1}H NMR (400 MHz, $CDCl_3$) δ 7.83 (s, 1H), 6.05 (s, 1H), 5.84 (d, J = 8.3 Hz, 1H), 5.49–5.36 (m, 2H), 5.24 (t, J = 9.4 Hz, 1H), 4.68 (2xbs, 2H), 4.32 (dd, J = 12.7, 4.7 Hz, 1H), 4.19–3.99 (m, 4H), 3.79 (bs, 1H), 2.46 (m, 2H), 2.37 (d, J = 10.6 Hz, 2H), 2.10 (s, 3H), 2.07 (s, 3H), 2.03 (s, 3H), 1.87 (s, 3H), 1.87 (m, 2H), 1.67 (2xd, J = 11.1 Hz, 4H), 1.31 (s, 3H), 1.43–1.12 (m, 7H), 0.90 (s, 3H). ^{13}C NMR (100 MHz, $CDCl_3$) δ 172.41, 170.63, 170.02, 169.42, 168.91, 167.70, 144.47, 142.53, 127.85, 121.66, 85.92, 75.30, 72.61, 70.54, 67.75, 61.57, 44.21, 40.38, 37.97, 32.96, 32.92, 31.83, 31.68, 25.96, 25.57, 24.89, 21.21, 21.15, 20.81, 20.63, 20.62, 20.23. HRMS (ESI-FT-ICR) *m/z*: Calcd. for $C_{35}H_{49}N_5O_{11}Na$ [M + Na]$^+$ 738.33263 found 738.33207.

(1R,1′R,5S,5′S)-N,N′-(hexa-2,4-diyne-1,6-diyl)bis(N-(2-(cyclohexylamino)-2-oxoethyl)-6,6-dimethylbicyclo[3.1.1]hept-2-ene-2-carboxamide) **(4a)**

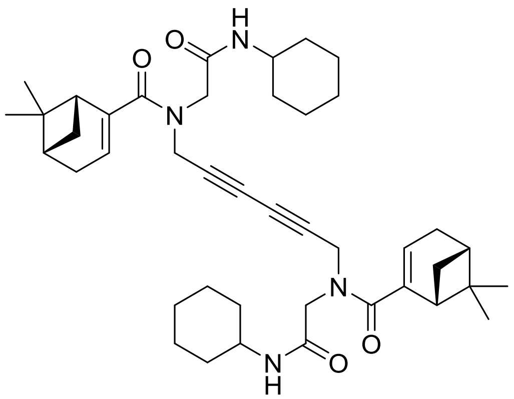

Paraformaldehyde (3.0 mg, 0.1 mmol), propargylamine (6.5 μL, 0.1 mmol), (1R,5S)-6,6-dimethylbicyclo[3.1.1]hept-2-ene-2-carboxylic acid (16.6 mg, 0.1 mmol), cyclohexyl isocyanide (12.4 μL, 0.1 mmol), piperidine (9.9 μL, 0.1 mmol), $CuAcO_2{\cdot}H_2O$ (2.0 mg, 0.1 mmol) were reacted according to the general tandem multicomponent-homocoupling procedure. Flash column chromatography purification (EtOAc/hexane = 1:1 v/v) afforded **4a** (55 mg, 80%) as an amorphous white solid. R_f = 0.35 (EtOAc/hexane = 1:1 v/v). ^{1}H NMR (400 MHz, $CDCl_3$) δ 6.26 (s, 2H), 6.06 (s, 2H), 4.40 (d, J = 18.2 Hz, 2H), 4.30 (d, J = 18.2 Hz, 2H), 4.13 (d, J = 15.7 Hz, 2H), 3.98 (d, J = 15.7 Hz, 2H), 3.76 (m, 2H), 2.60–2.31 (m, 8H), 2.16 (d, J = 7.1 Hz, 2H), 1.93–1.79 (m, 6H), 1.73–1.53 (m, 6H), 1.33 (s, 6H), 1.44–1.10 (m, 15H), 0.91 (s, 6H). ^{13}C NMR (100 MHz, $CDCl_3$) δ 171.63, 167.43, 142.28, 128.44, 73.93, 68.79, 60.49, 48.36, 44.20, 40.36, 37.98, 32.96, 31.89, 31.74, 25.95, 25.56, 24.80, 21.17, 14.29. HRMS (ESI-FT-ICR) *m/z*: Calcd. for $C_{42}H_{58}N_4O_4H$ [M + H]$^+$ 683.45363 found 683.45308.

2,2′-(hexa-2,4-diyne-1,6-diylbis(acetylazanediyl))bis(N-cyclohexyl-2-((1R,5S)-6,6-dimethylbicyclo[3.1.1]hept-2-en-2-yl)acetamide) **(4b)**

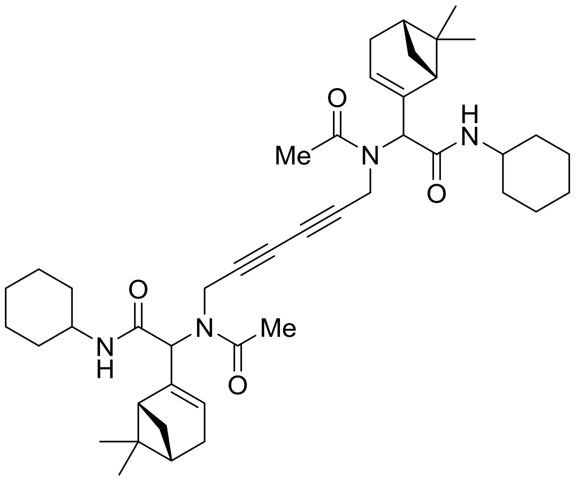

(1*R*)-(−)-Myrtenal (15.2 μL, 0.1 mmol), propargylamine (6.5 μL, 0.1 mmol), acetic acid (5.7 μL, 0.1 mmol) and cyclohexyl isocyanide (12.4 μL, 0.1 mmol), piperidine (9.9 μL, 0.1 mmol), $CuAcO_2{\cdot}H_2O$ (2.0 mg, 0.1 mmol) were reacted according to the general tandem multicomponent-homocoupling procedure. Flash column chromatography purification (EtOAc/hexane = 1:1 v/v) afforded **4b** (43 mg, 60%) as an amorphous white solid. R_f = 0.40 (EtOAc/hexane = 1:1 v/v). A mixture of diastereomers in a 1:0.9 ratio was observed by NMR analysis. ^{1}H NMR (400 MHz, $CDCl_3$) δ 5.67 (m, 6H), 5.38 (s, 2H), 5.26 (s, 2H), 4.41 (dd, *J* = 19.3, 3.7 Hz, 2H), 4.27 (d, *J* = 20.2 Hz, 2H), 4.17 (d, *J* = 9.6 Hz, 2H), 3.80–3.68 (m, 4H), 2.45 (m, 4H), 2.29 (m, 4H), 2.26 (s, 6H), 2.25 (s, 6H), 2.15–2.02 (m, 10H), 1.96–1.79 (m, 12H), 1.76–1.57 (m, 12H), 1.29 (s, 6H), 1.28 (s, 6H), 1.42–1.06 (m, 26H), 0.88 (s, 6H), 0.86 (s, 6H). ^{13}C NMR (100 MHz, $CDCl_3$) δ 172.23, 172.03, 168.50, 168.08, 142.54, 141.69, 124.86, 124.69, 75.25, 75.00, 68.43, 67.94, 61.99, 61.36, 48.62, 48.39, 44.93, 44.14, 40.44, 40.36, 38.21, 37.98, 37.14, 36.25, 33.09, 32.96, 32.85, 32.10, 31.89, 31.68, 31.04, 26.12, 25.59, 24.93, 24.80, 22.24, 21.12, 21.06. HRMS (ESI-FT-ICR) *m/z*: Calcd. for $C_{44}H_{62}N_4O_4H$ $[M + H]^+$ 711.48493 found 711.48438.

Table S1. EC_{50} and E_{max} values of derivative against different cancer cell lines

Myrtenyl derivatives	AGS EC_{50} (nM) / E_{max}	MCF–7 EC_{50} (nM) / E_{max}	HT29 EC_{50} (nM) / E_{max}
**1a**	46 ± 6 / 0.1	65 ± 9 / 0.1	68 ± 7 / 0.1
**3a**	50 ± 16 / 0.1	24 ± 5 / 0.3	59 ± 13 / 0.3
**3b**	49 ± 8 / 0.2	24 ± 7 / 0.5	21 ± 7 / 0.4
**3c**	75 ± 4 / 0.1	38 / 0.4	38 ± 1 / 0.4
**3d**	33 ± 3 / 0.1	22 ± 2 / 0.1	33 ± 6 / 0.1

Spectra of selected compounds

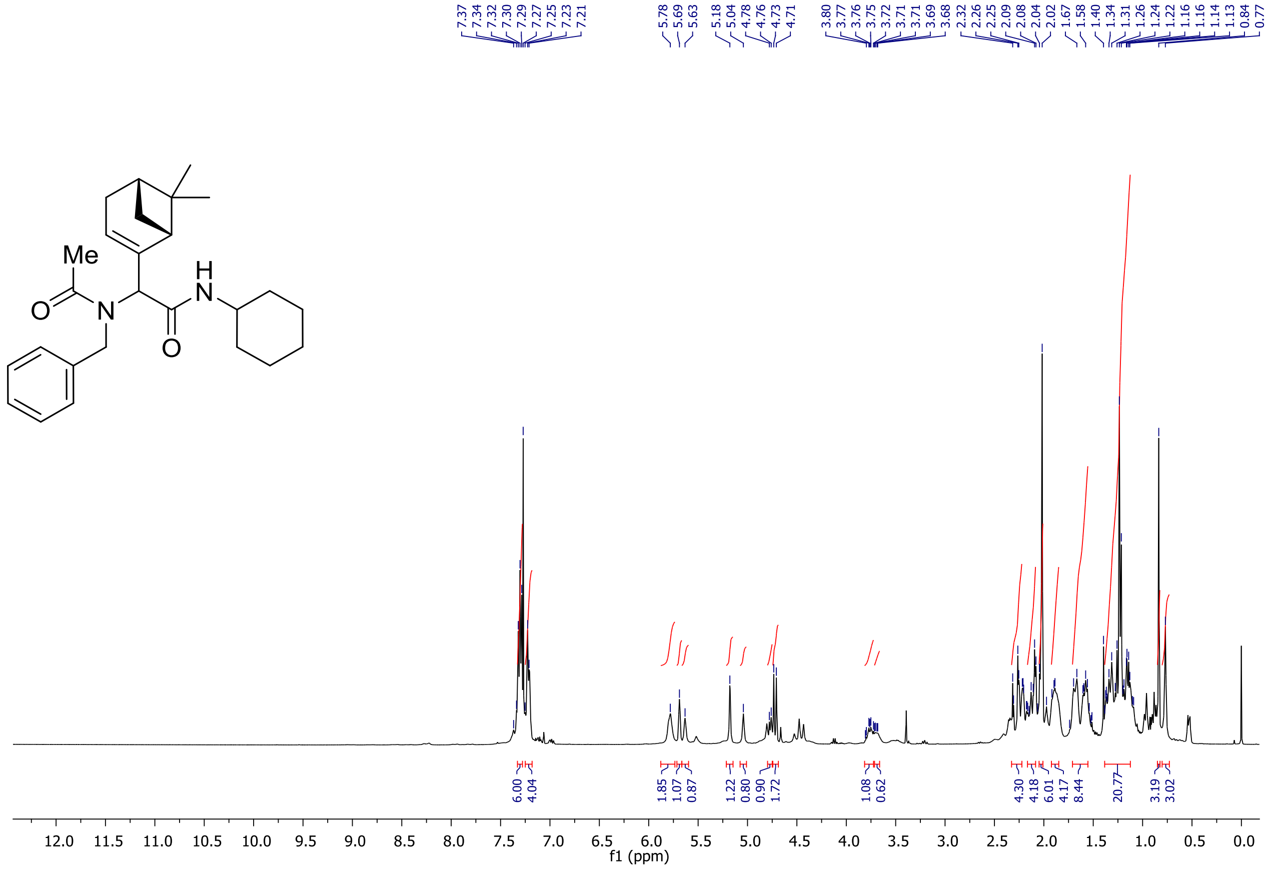

FIGURE S1. 400 MHz ^{1}H NMR spectra in $CDCl_3$ of **1a** (Crude mixture of diastereomers).

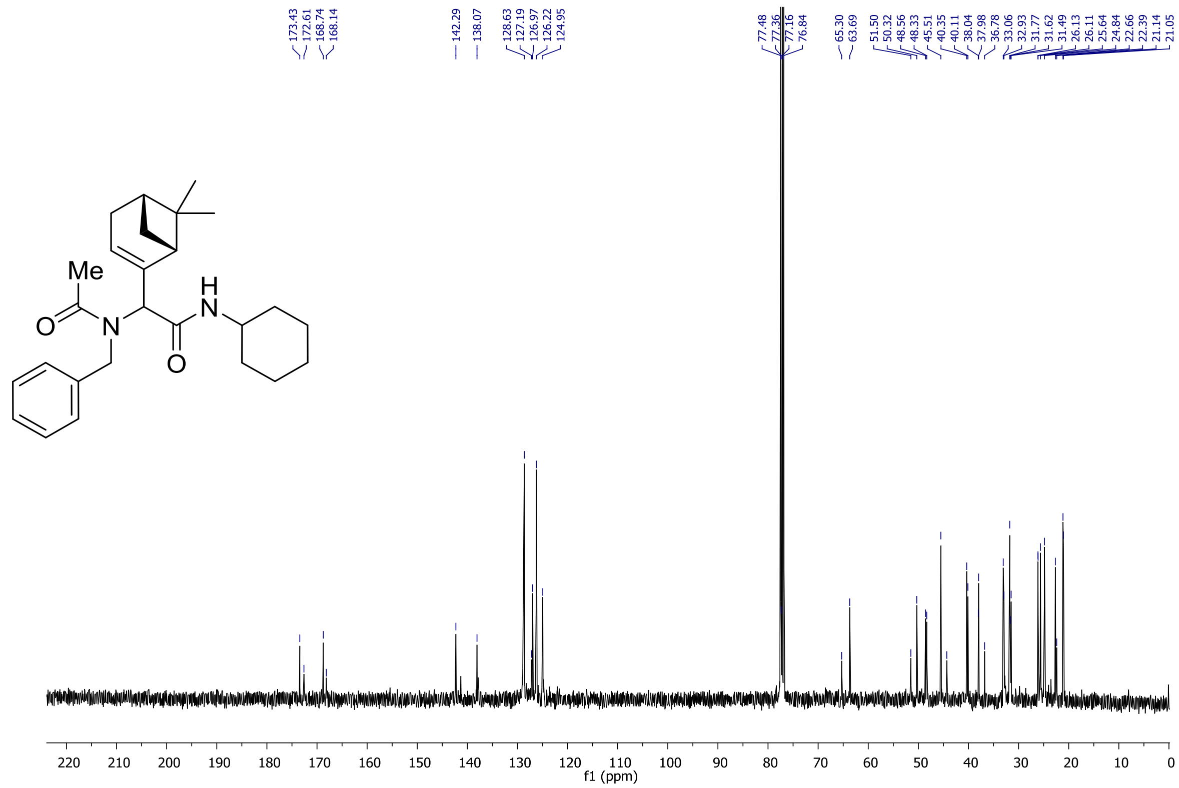

FIGURE S2. 100 MHz ^{13}C NMR spectra in $CDCl_3$ of **1a** (Crude mixture of diastereomers).

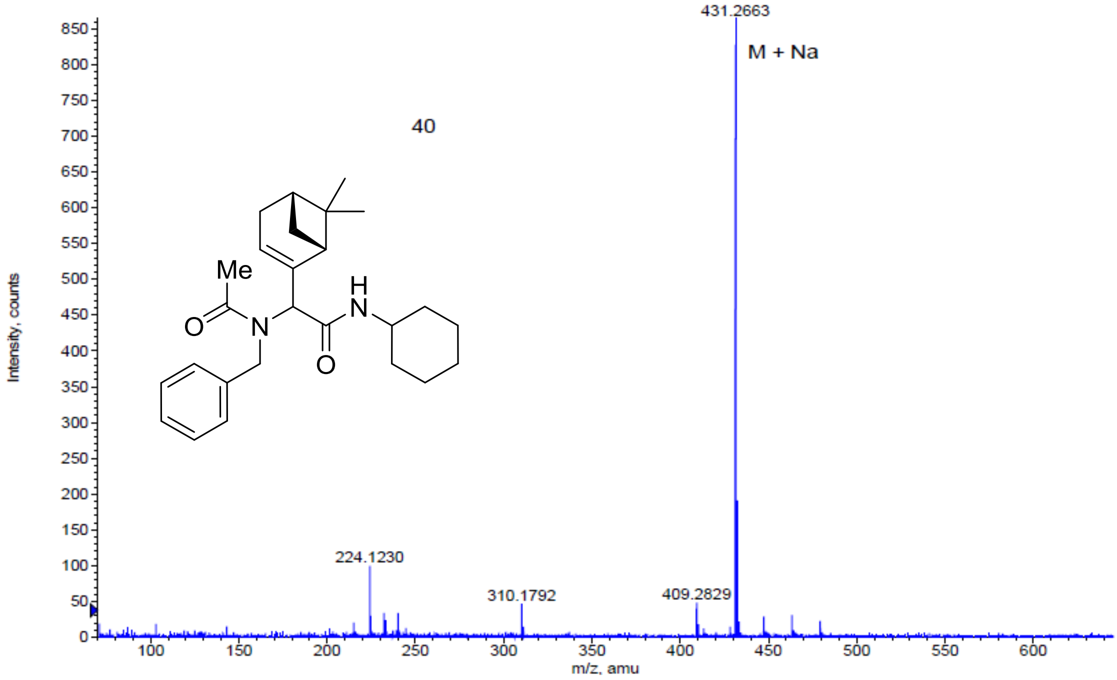

FIGURE S3. HRMS (ESI-FT-ICR) *m/z* spectra of **1a**.

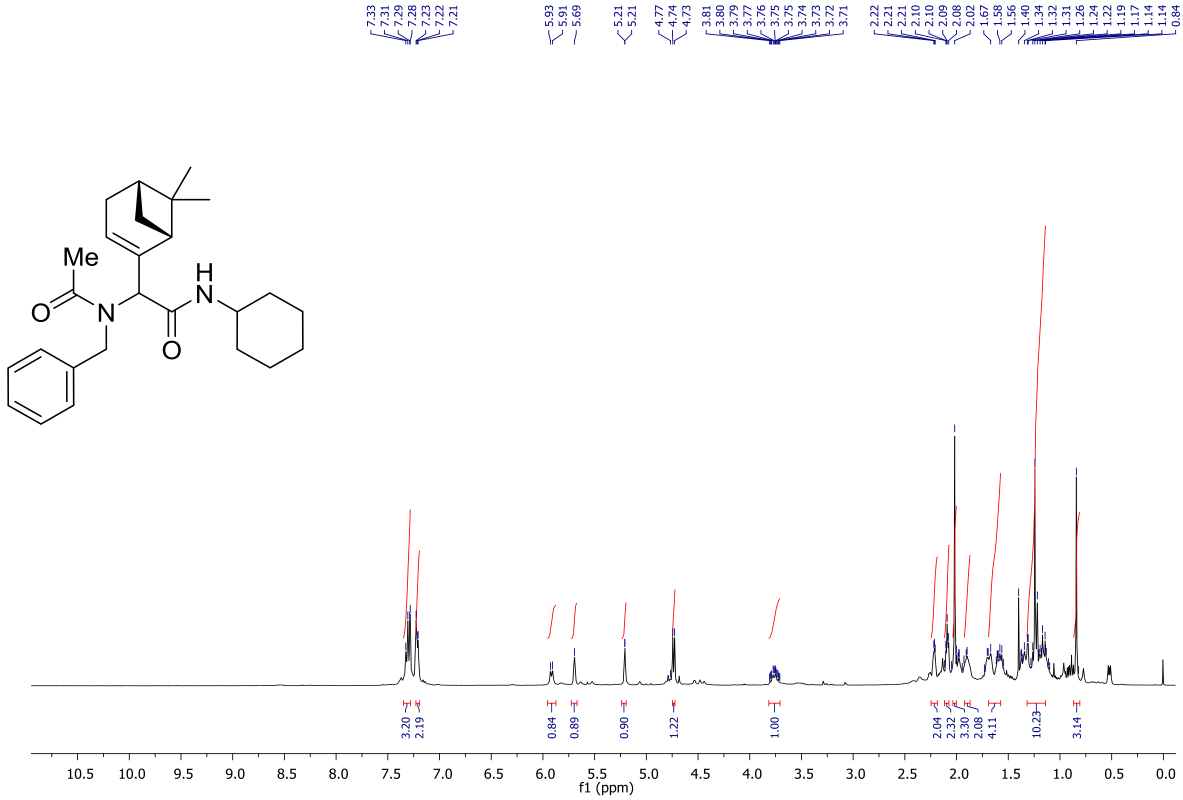

FIGURE S4. 400 MHz ^{1}H NMR spectra in $CDCl_3$ of major diastereomer of **1a**.

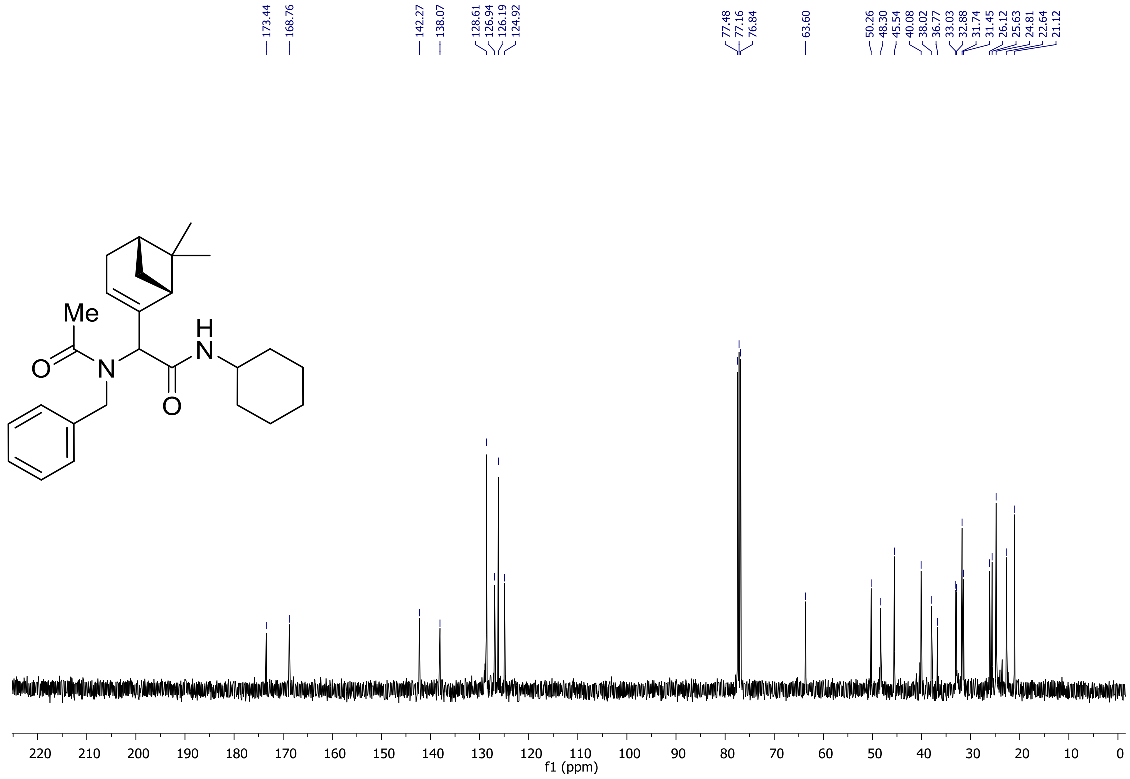

FIGURE S5. 100 MHz ^{13}C NMR spectra in $CDCl_3$ of major diastereomer of **1a**.

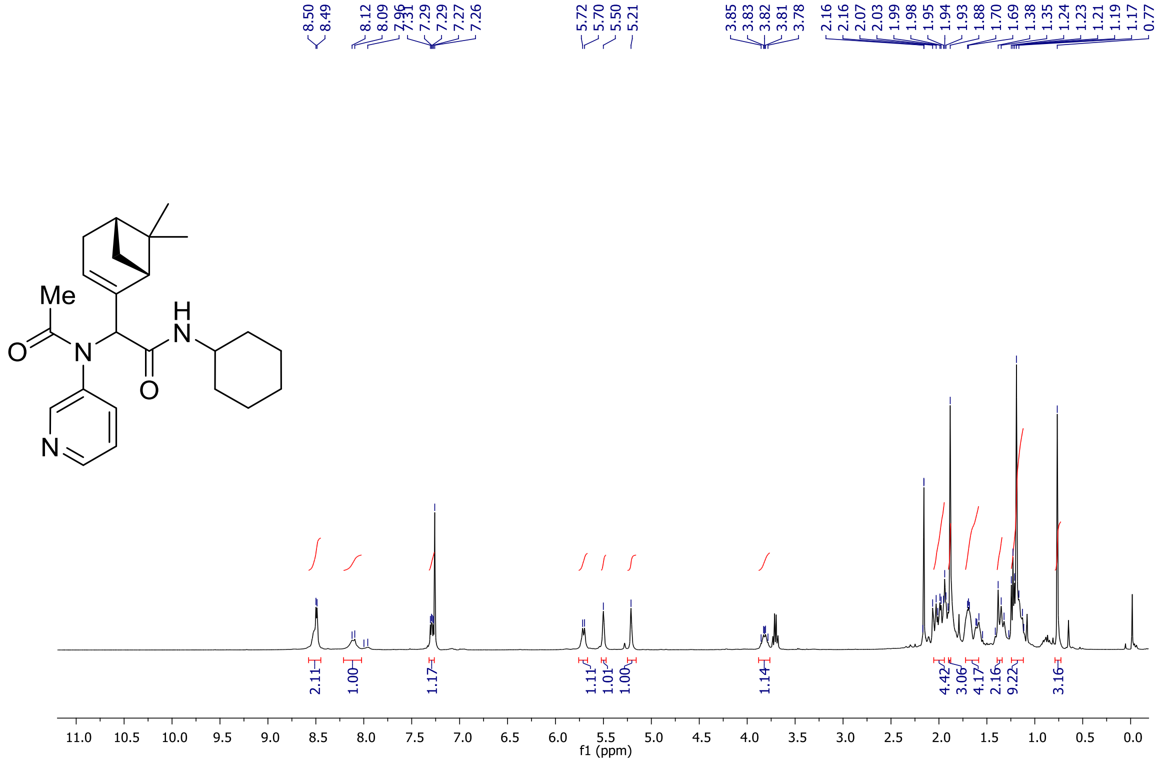

FIGURE S6. 400 MHz ^{1}H NMR spectra in $CDCl_3$ of **1b** (Crude mixture of diastereomers).

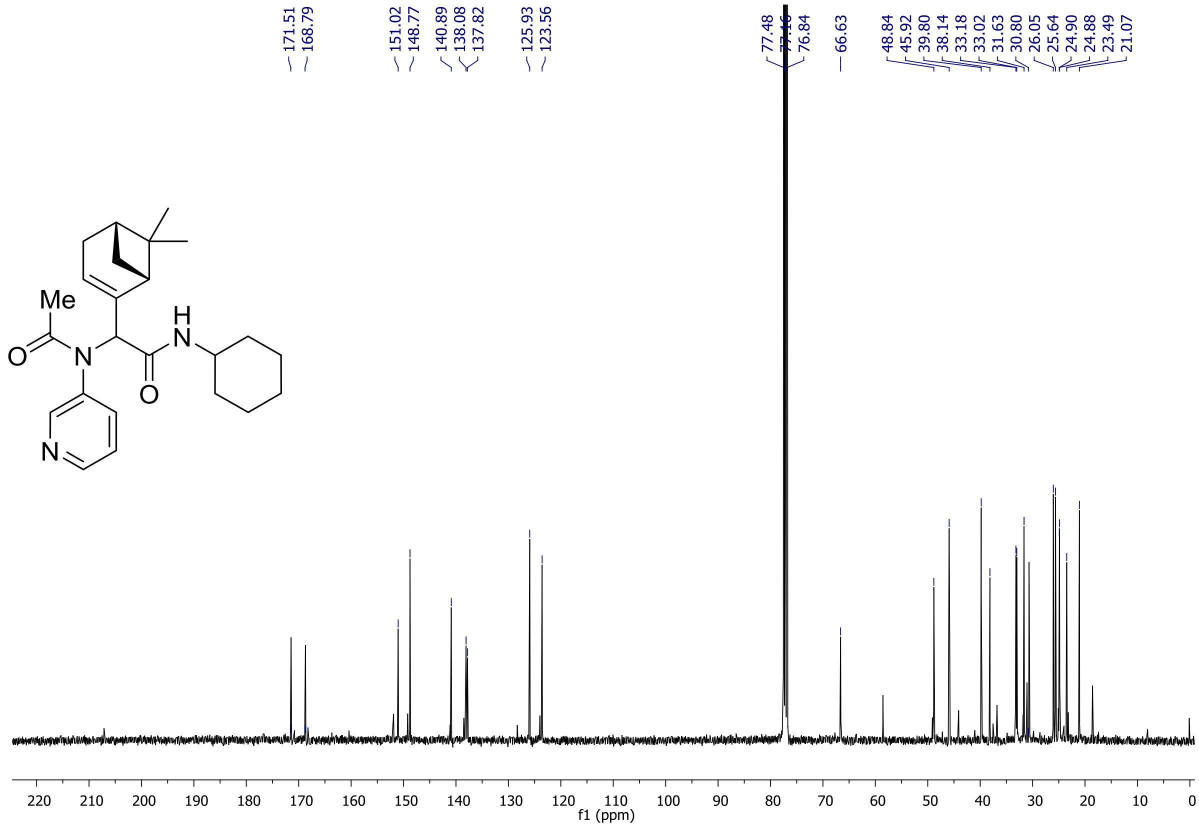

FIGURE S7. 100 MHz ^{13}C NMR spectra in $CDCl_3$ of **1b** (Crude mixture of diastereomers).

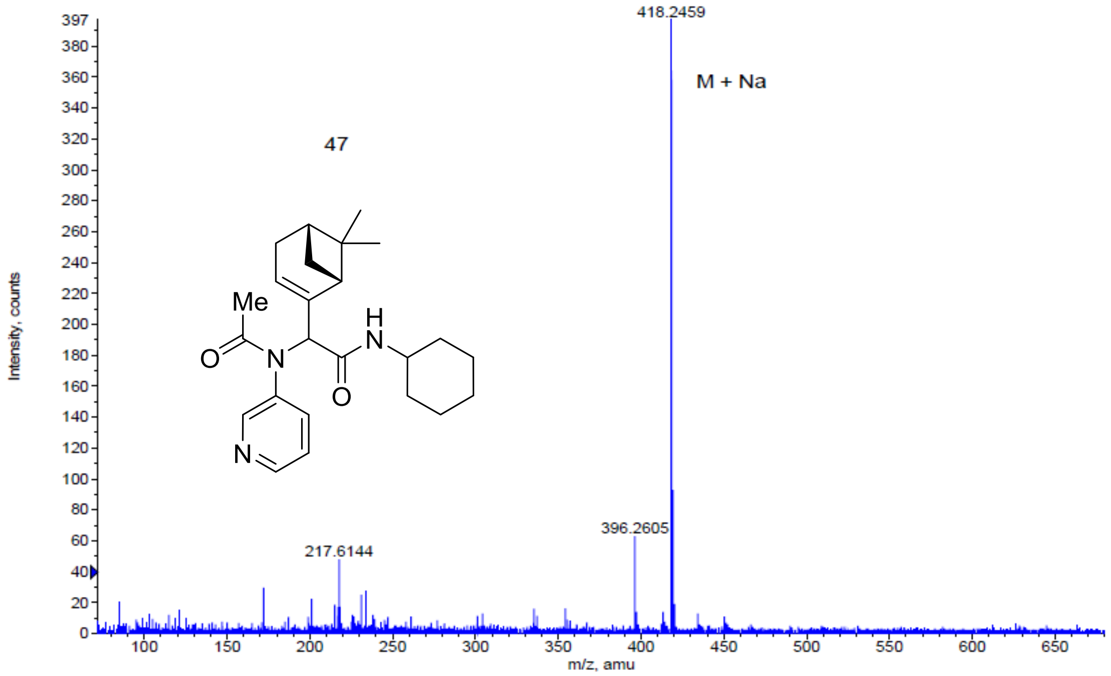

FIGURE S8. HRMS (ESI-FT-ICR) *m*/*z* spectra of **1b**.

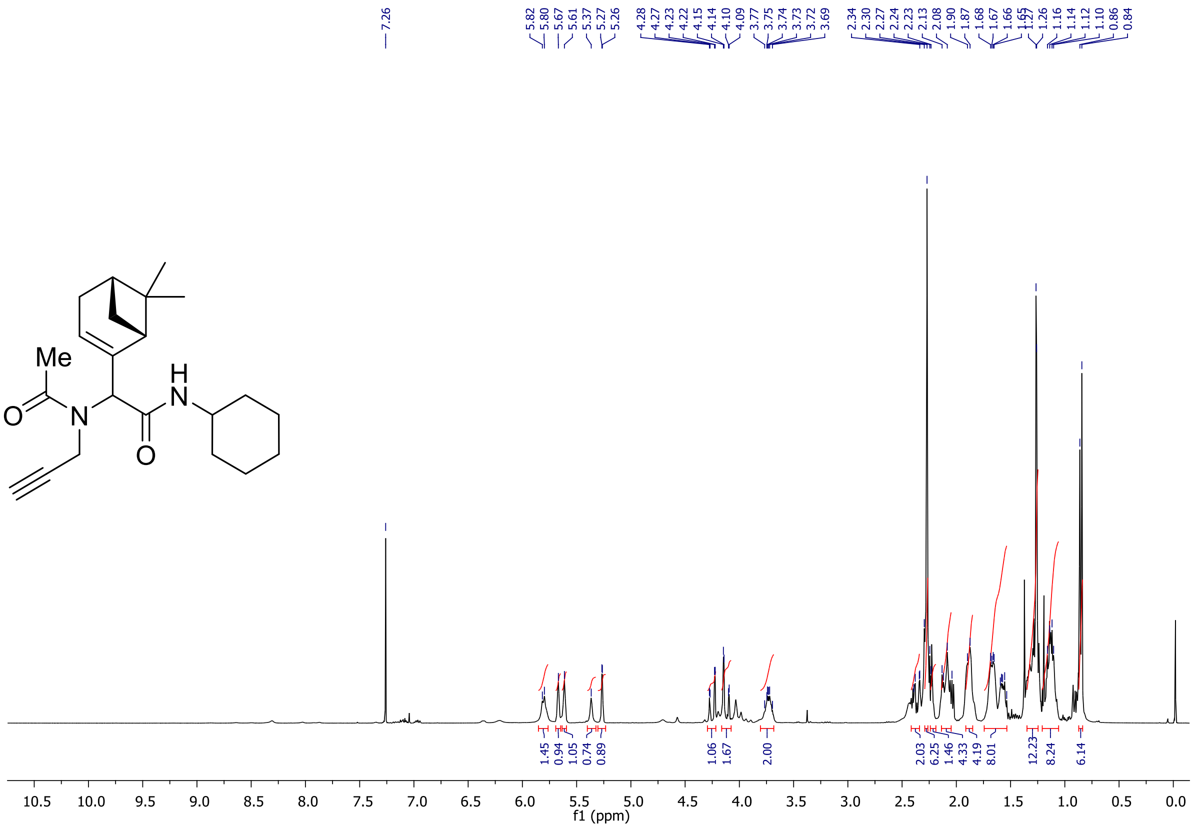

FIGURE S9. 400 MHz ^{1}H NMR spectra in $CDCl_3$ of **1c** (Crude mixture of diastereomers).

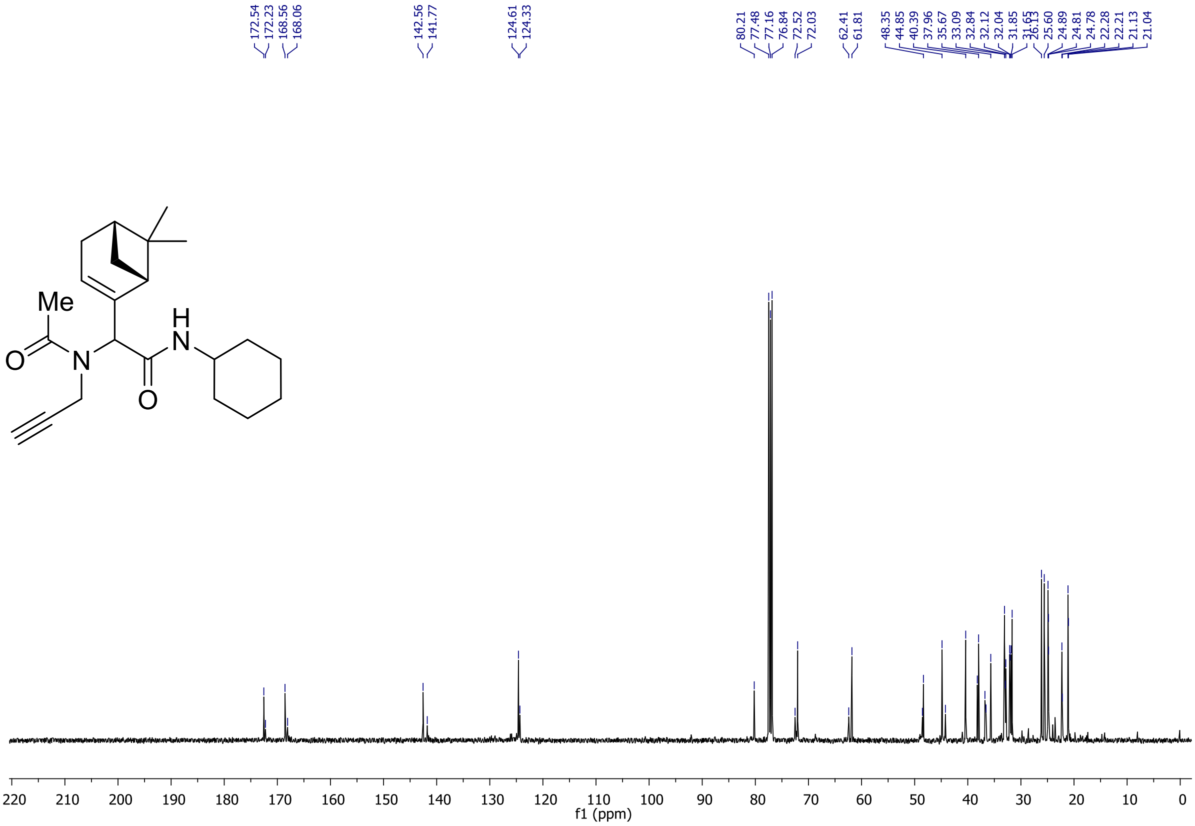

FIGURE S10. 100 MHz ^{13}C NMR spectra in $CDCl_3$ of **1c** (Crude mixture of diastereomers).

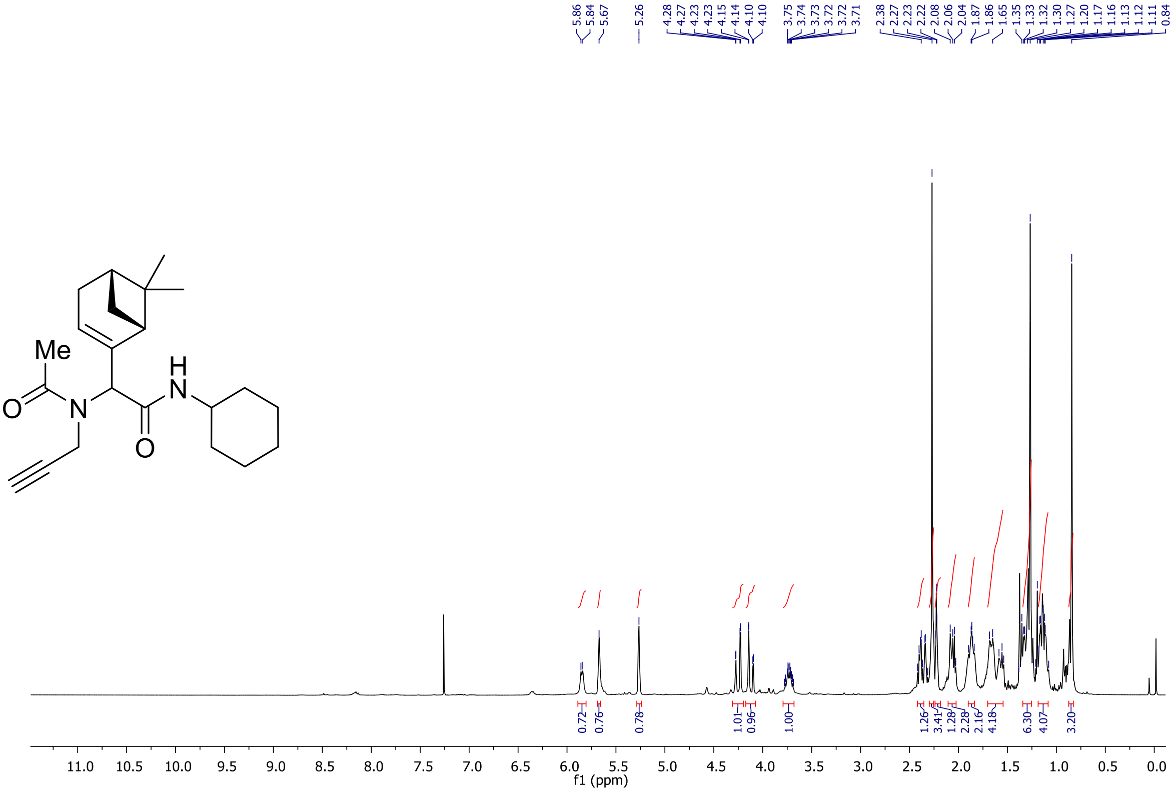

FIGURE S11. 400 MHz ^{1}H NMR spectra in $CDCl_3$ of major diastereomer of **1c.**

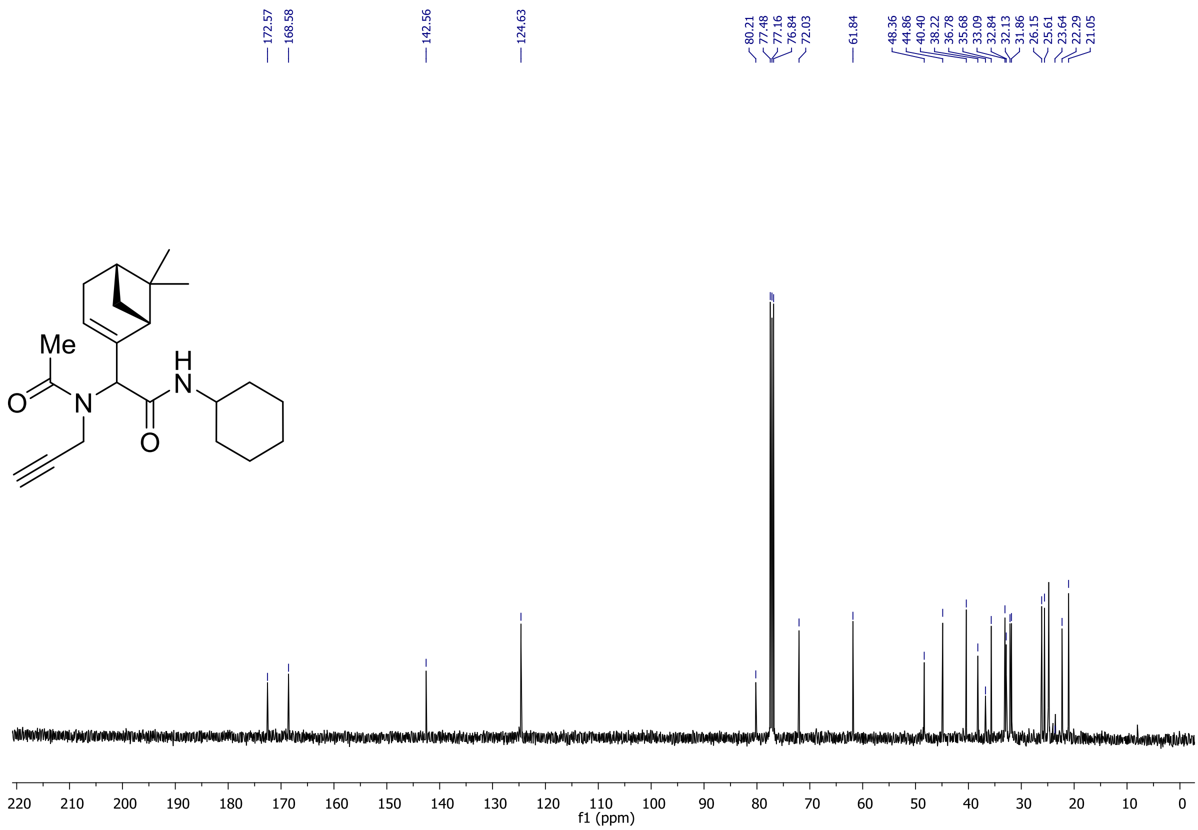

FIGURE S12. 100 MHz ^{13}C NMR spectra in $CDCl_3$ of major diastereomer of **1c.**

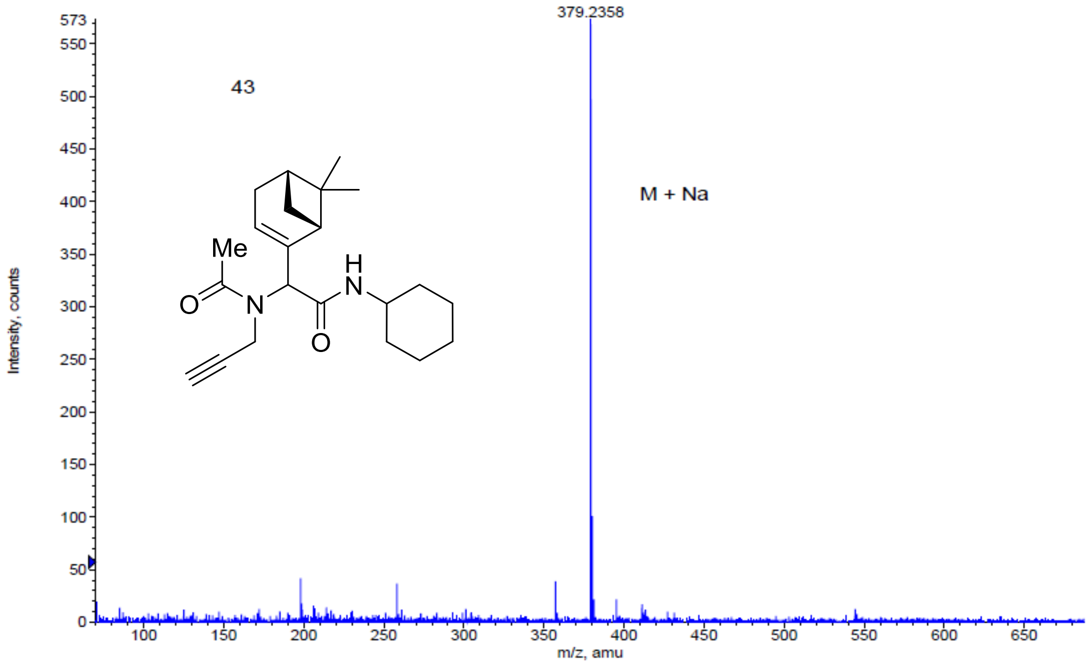

FIGURE S13. HRMS (ESI-FT-ICR) *m*/*z* spectra of **1c**.

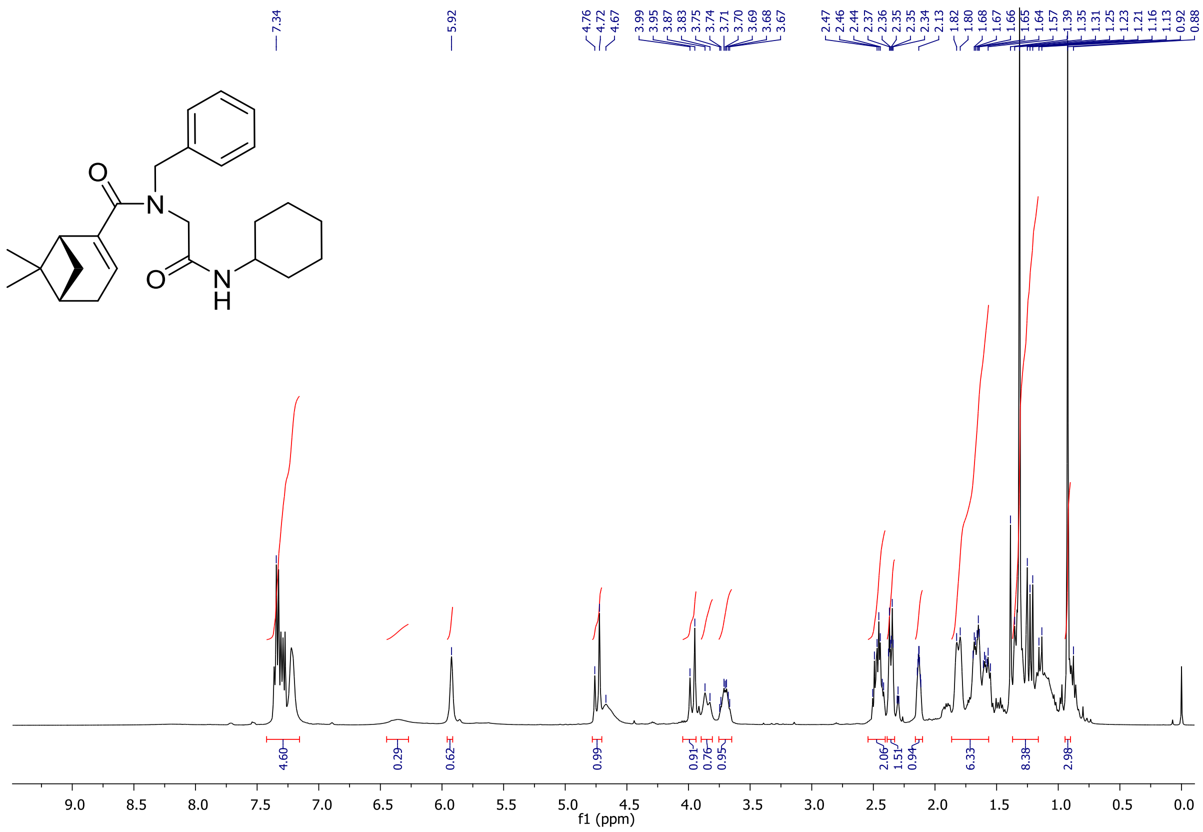

FIGURE S14. 400 MHz ^{1}H NMR spectra in $CDCl_3$ of **2a**.

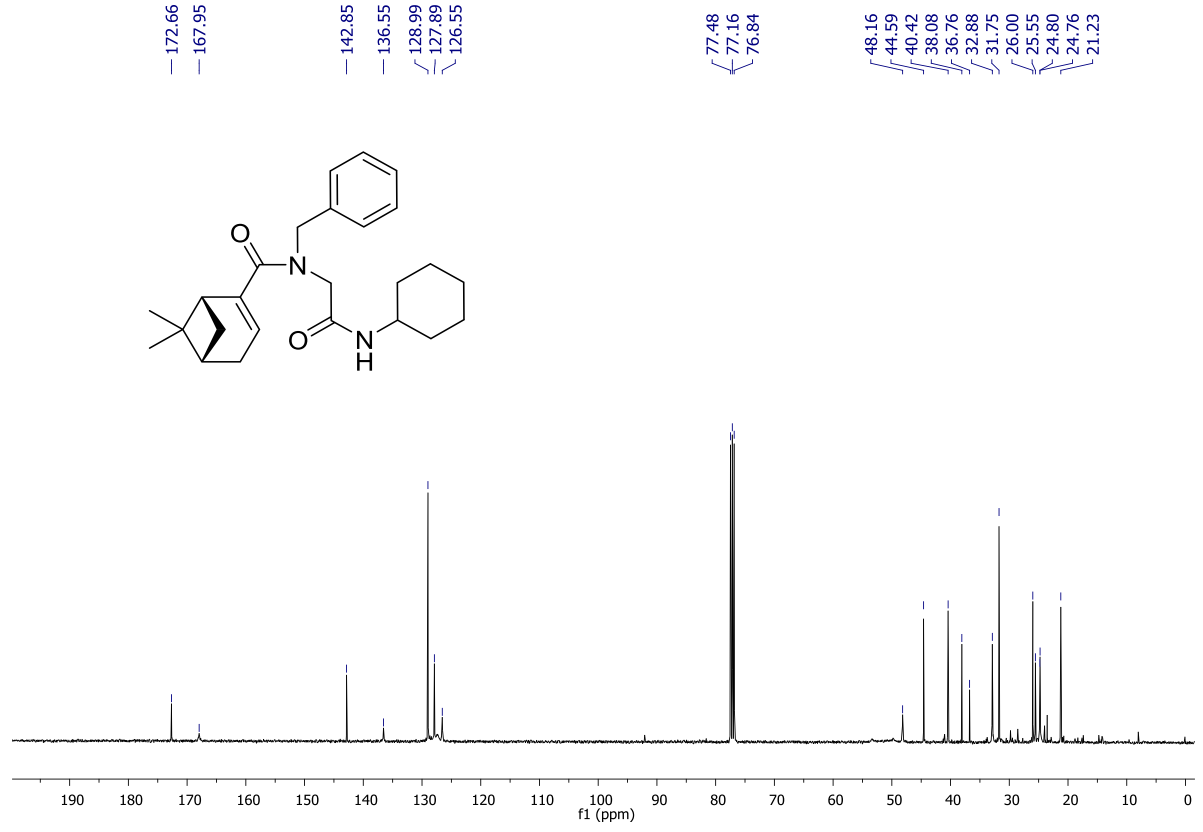

FIGURE S15. 100 MHz ^{13}C NMR spectra in $CDCl_3$ of **2a**.

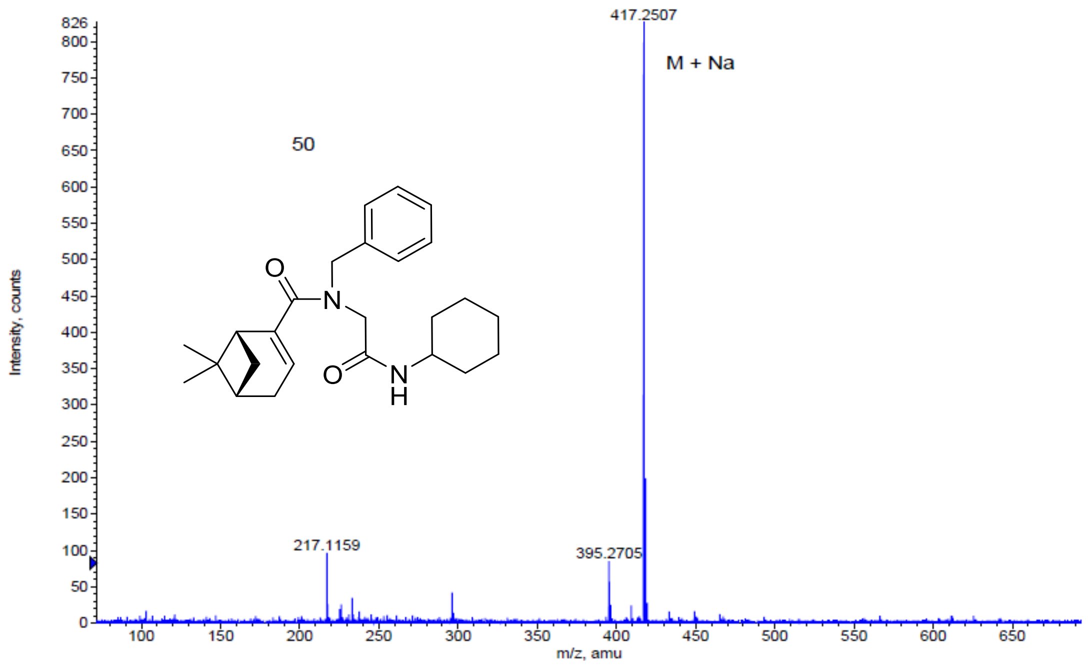

FIGURE S16. HRMS (ESI-FT-ICR) *m/z* spectra of **2a**.

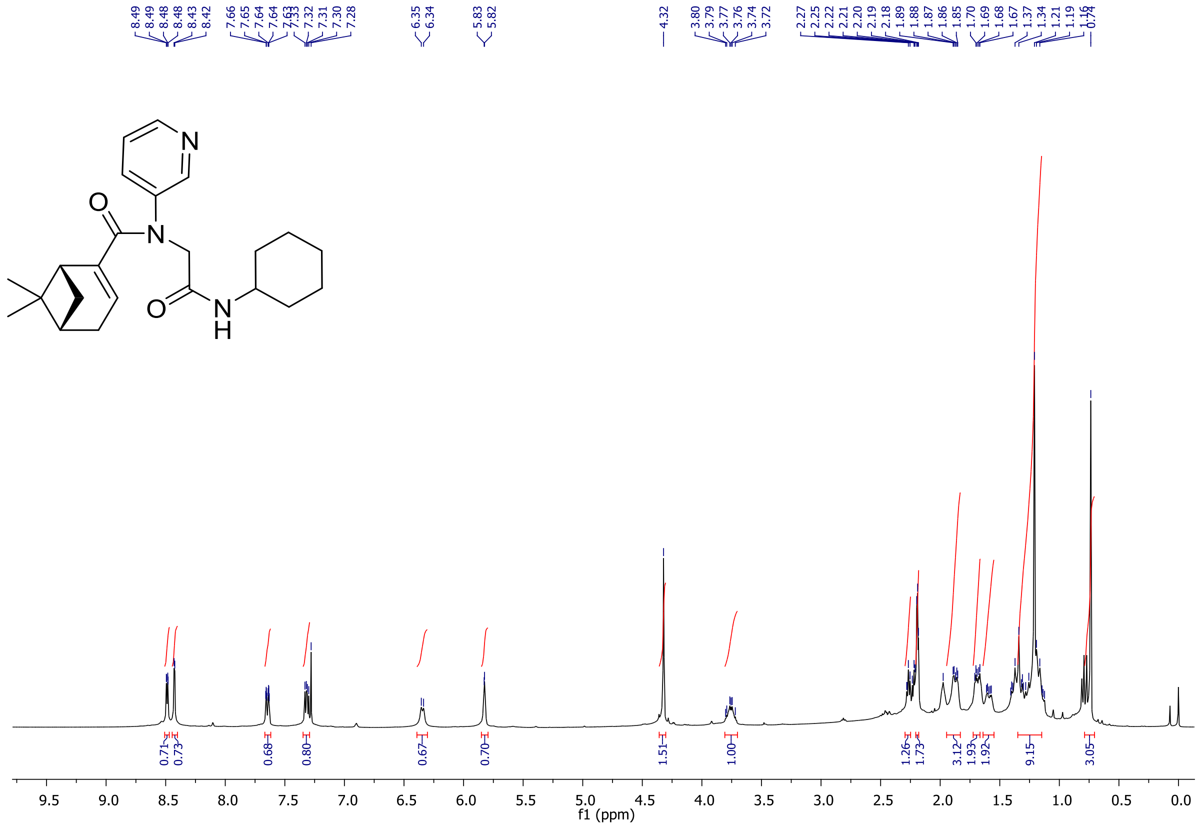

FIGURE S17. 400 MHz ^{1}H NMR spectra in $CDCl_3$ of **2b**.

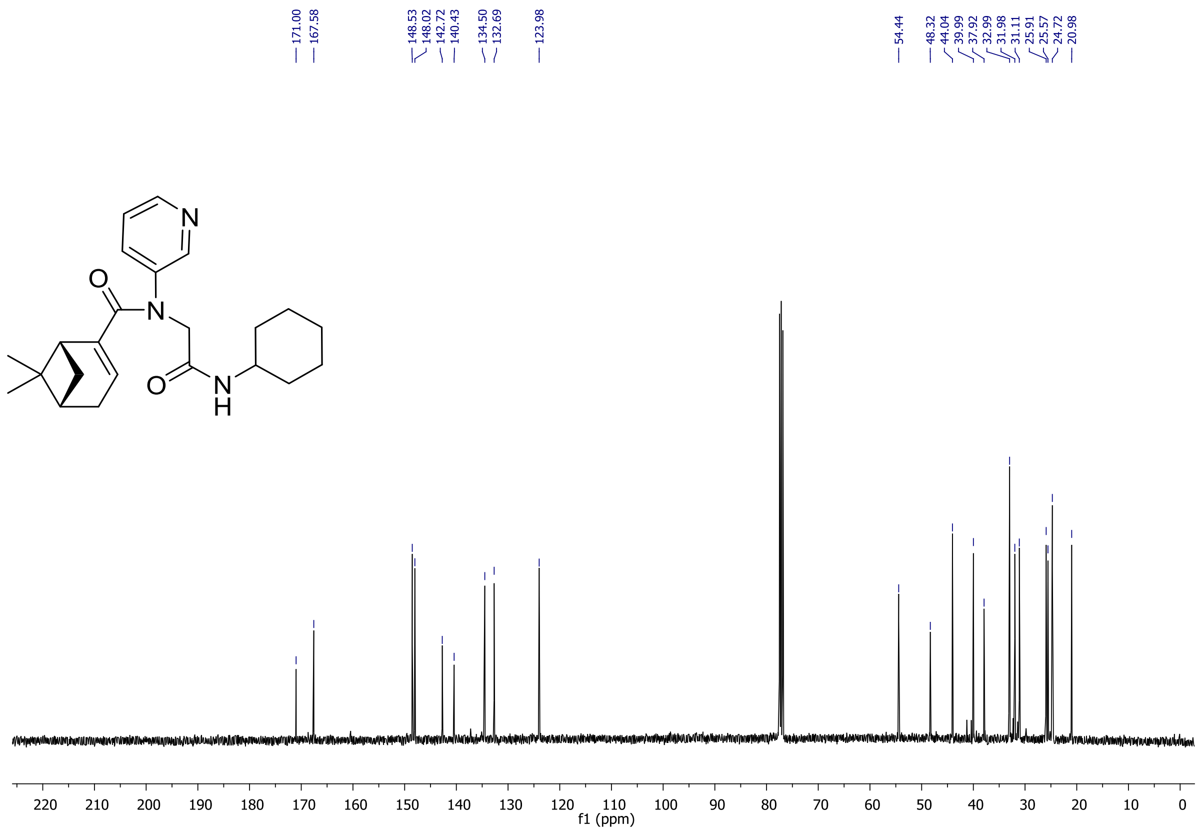

FIGURE S18. 100 MHz ^{13}C NMR spectra in $CDCl_3$ of **2b**.

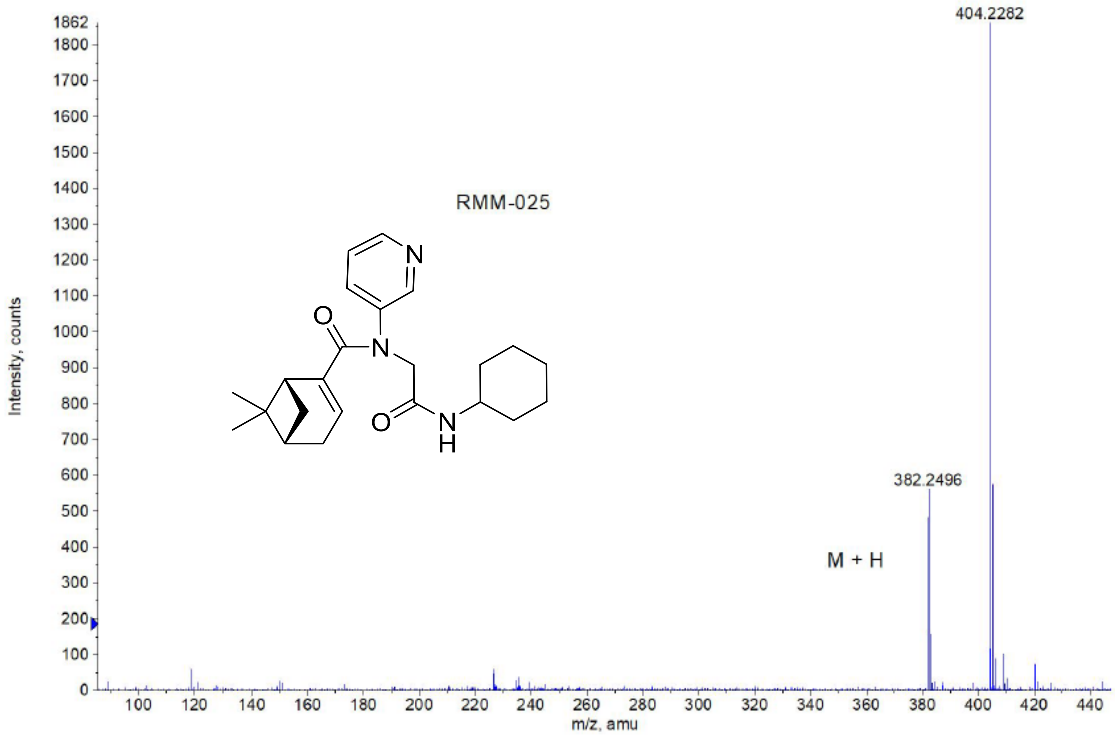

FIGURE S19. HRMS (ESI-FT-ICR) *m*/*z* spectra of **2b**.

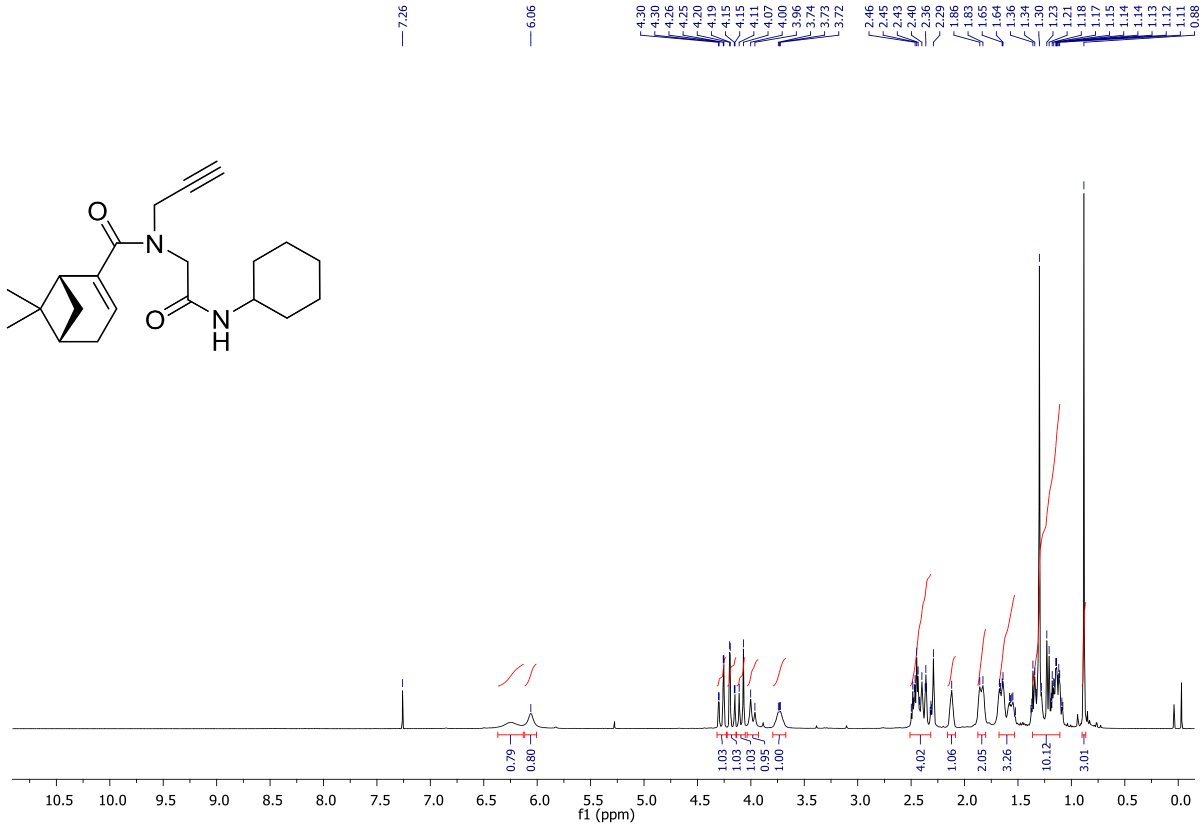

FIGURE S20. 400 MHz ^{1}H NMR spectra in $CDCl_3$ of **2c**.

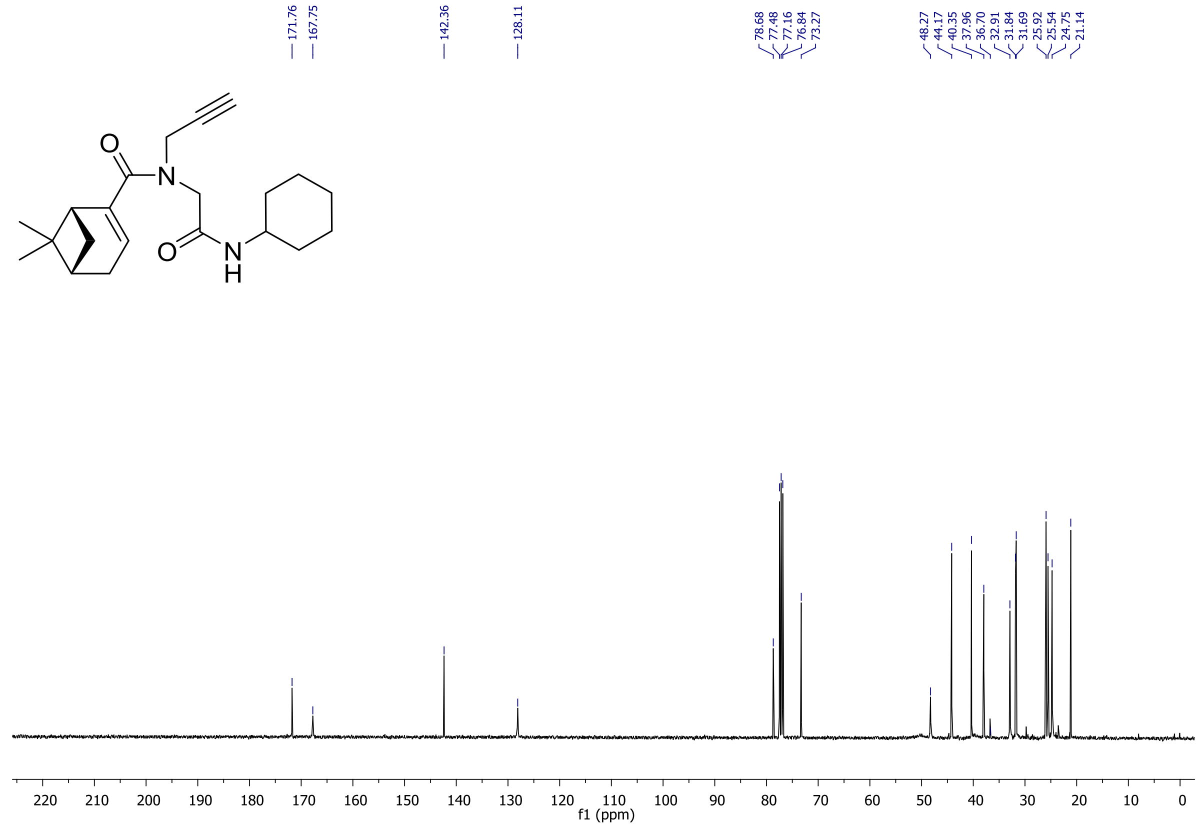

FIGURE S21. 100 MHz ^{13}C NMR spectra in $CDCl_3$ of **2c**.

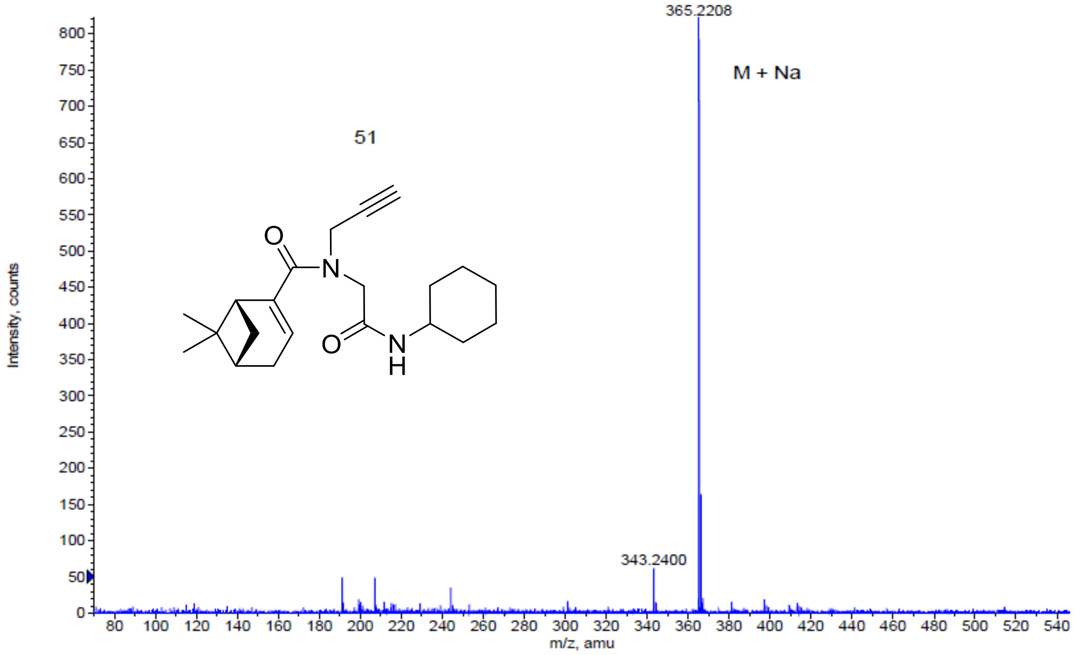

FIGURE S22. HRMS (ESI-FT-ICR) *m*/*z* spectra of **2c**.

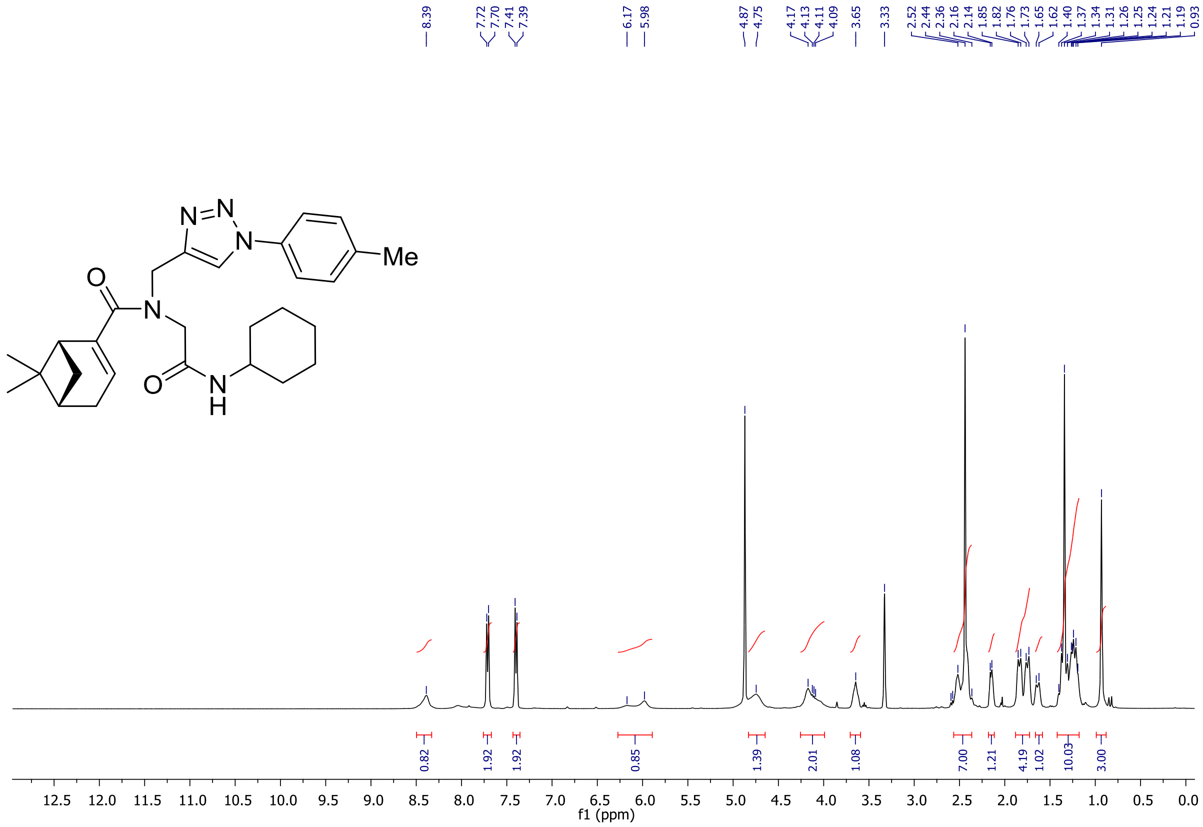

FIGURE S23. 400 MHz ^{1}H NMR spectra in CD_3OD of **3a**.

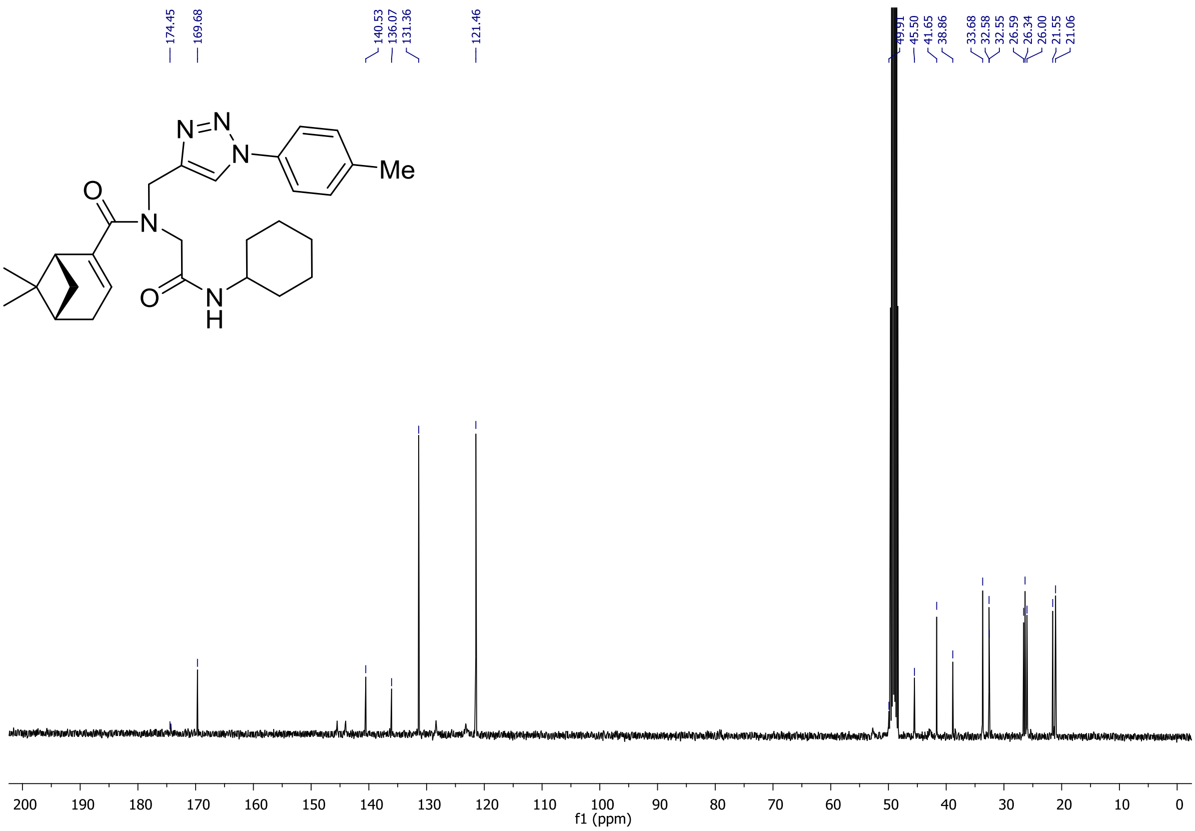

FIGURE S24. 100 MHz ^{13}C NMR spectra in CD_3OD of **3a**.

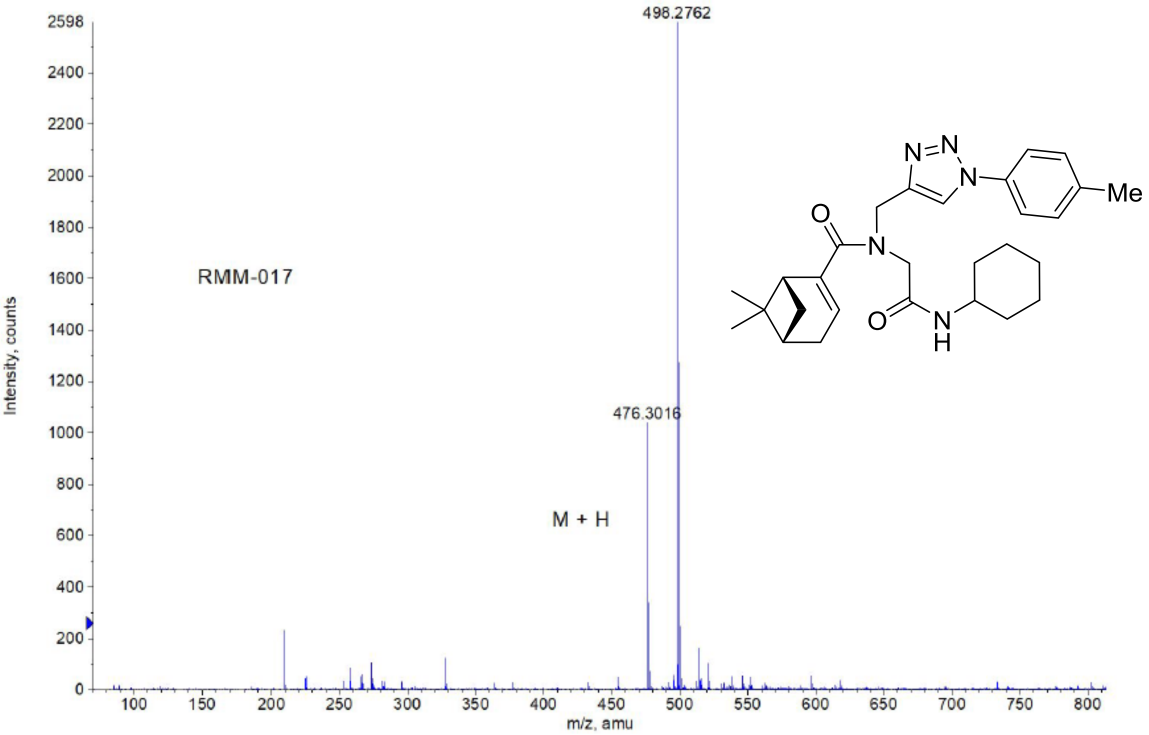

FIGURE S25. HRMS (ESI-FT-ICR) *m*/*z* spectra of **3a**.

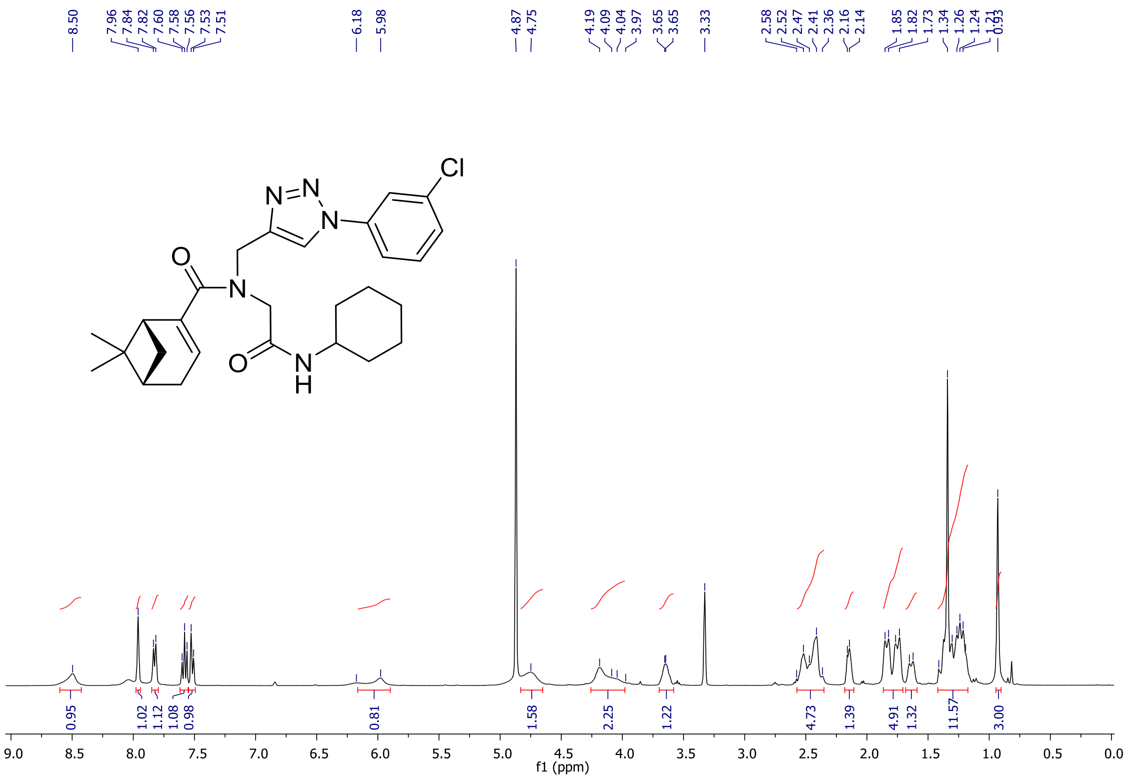

FIGURE S26. 400 MHz ^{1}H NMR spectra in CD_3OD of **3b**.

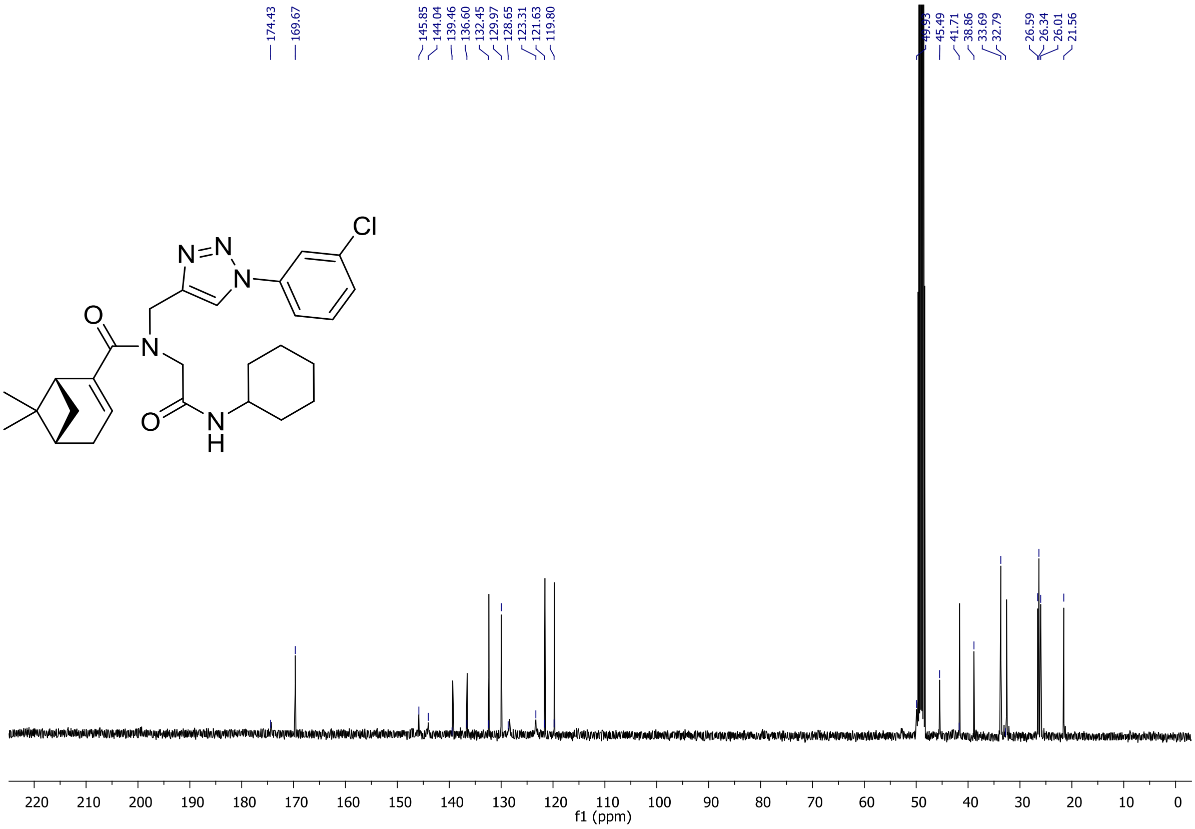

FIGURE S27. 100 MHz ^{13}C NMR spectra in CD_3OD of **3b**.

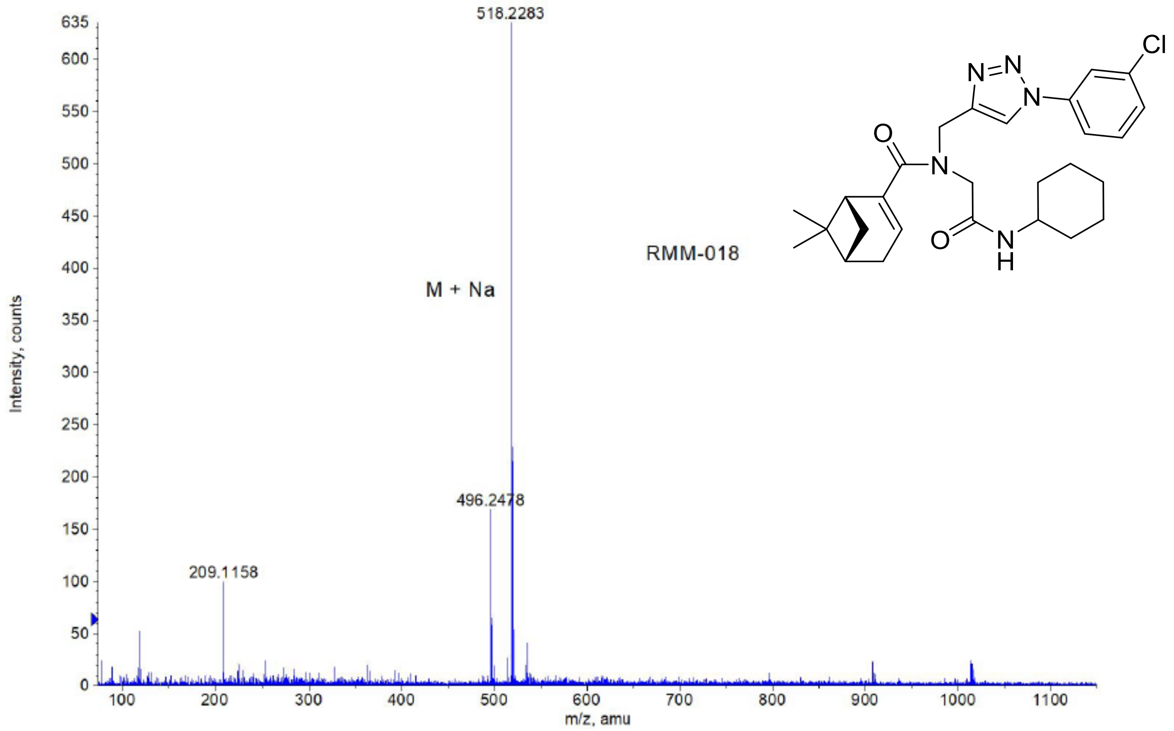

FIGURE S28. HRMS (ESI-FT-ICR) *m*/*z* spectra of **3b**.

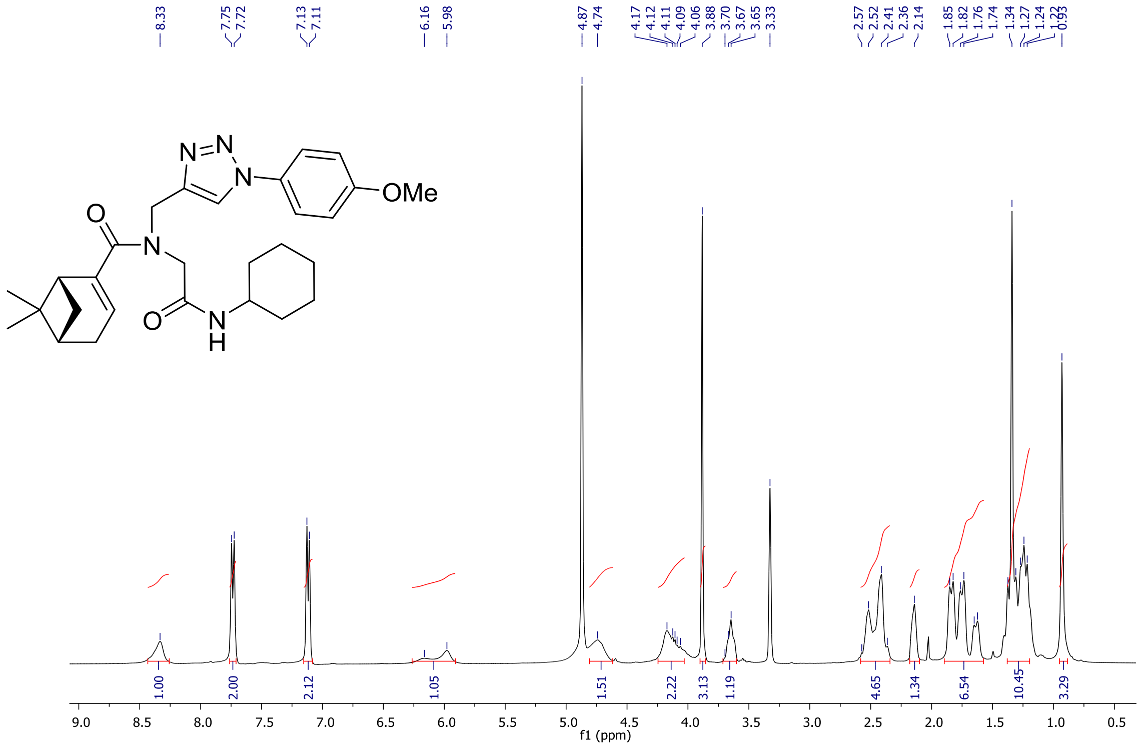

FIGURE S29. 400 MHz ^{1}H NMR spectra in MeOD of **3c**.

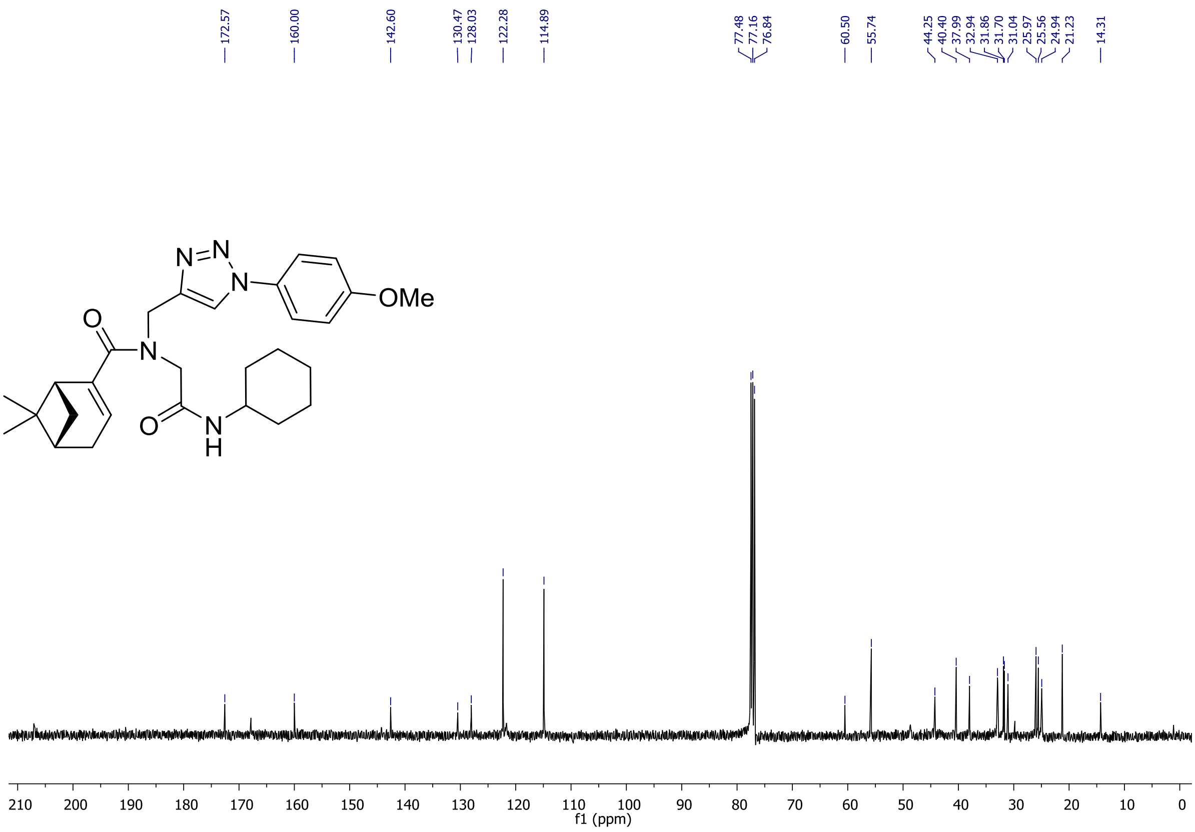

FIGURE S30. 100 MHz ^{13}C NMR spectra in $CDCl_3$ of **3c**.

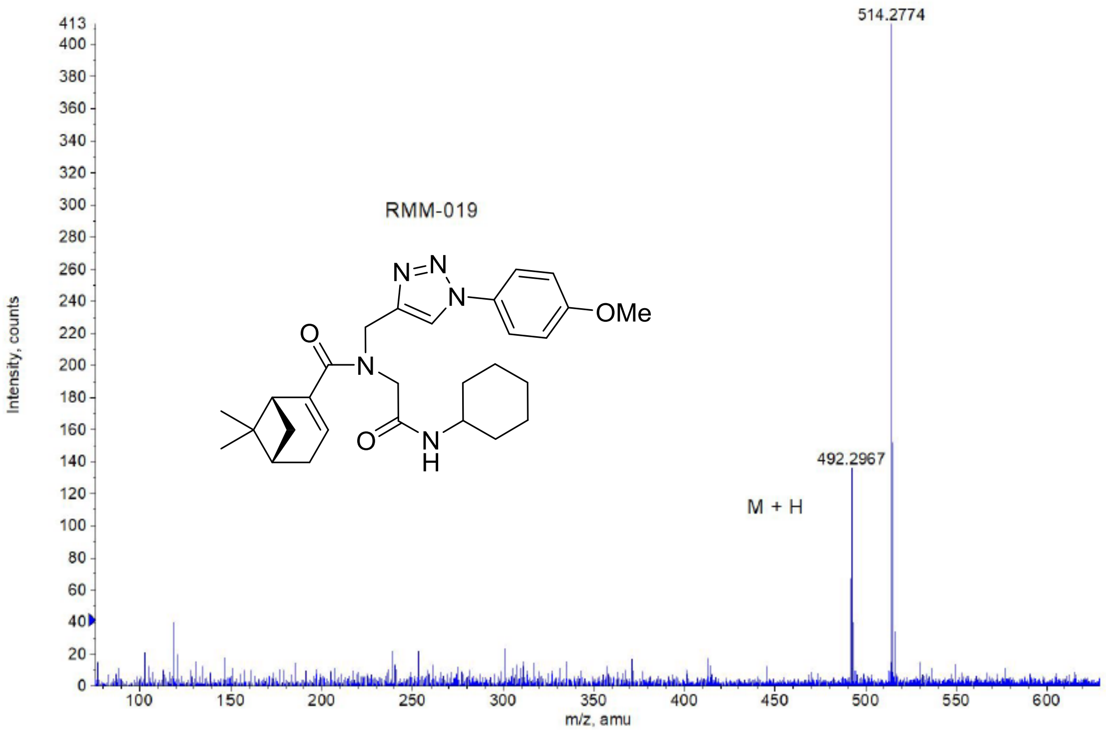

FIGURE S31. HRMS (ESI-FT-ICR) *m*/*z* spectra of **3c**.

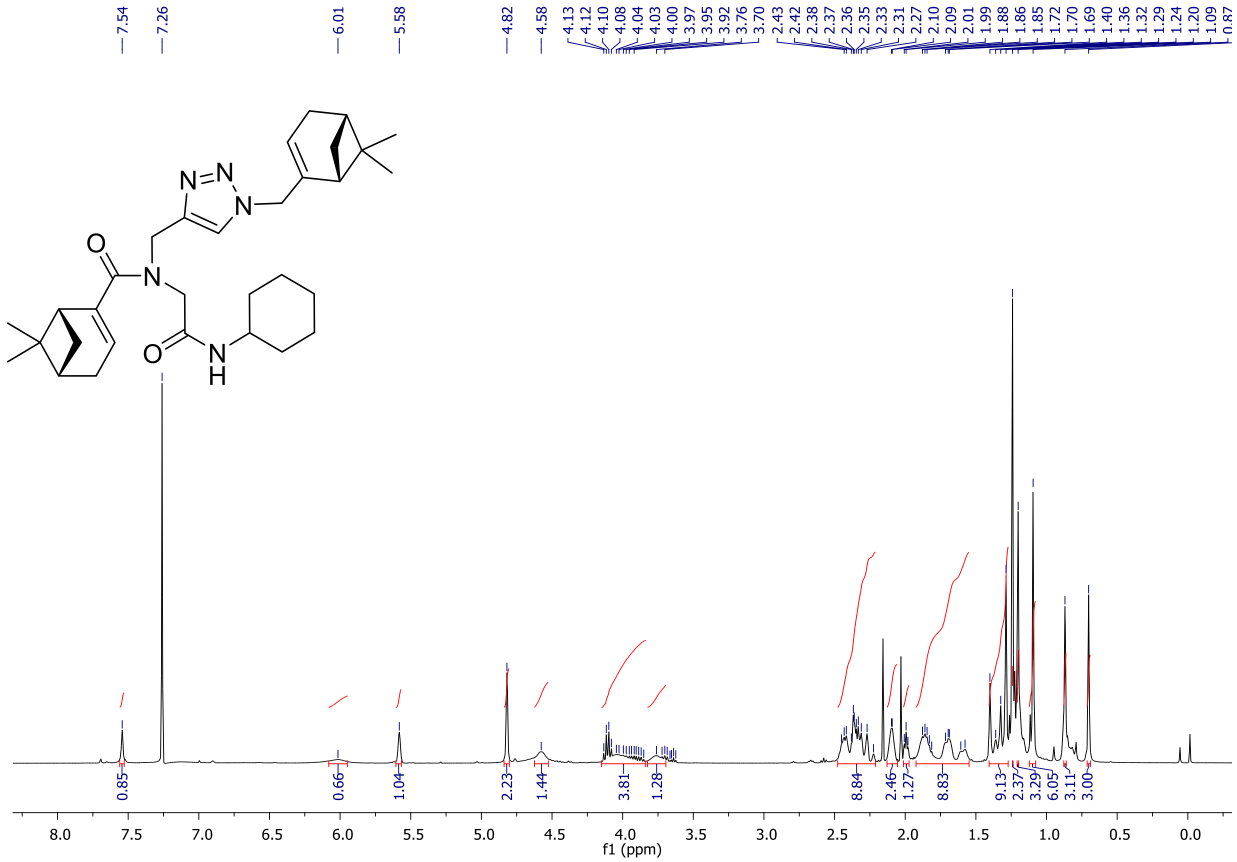

FIGURE S32. 400 MHz ^{1}H NMR spectra in $CDCl_3$ of **3d**.

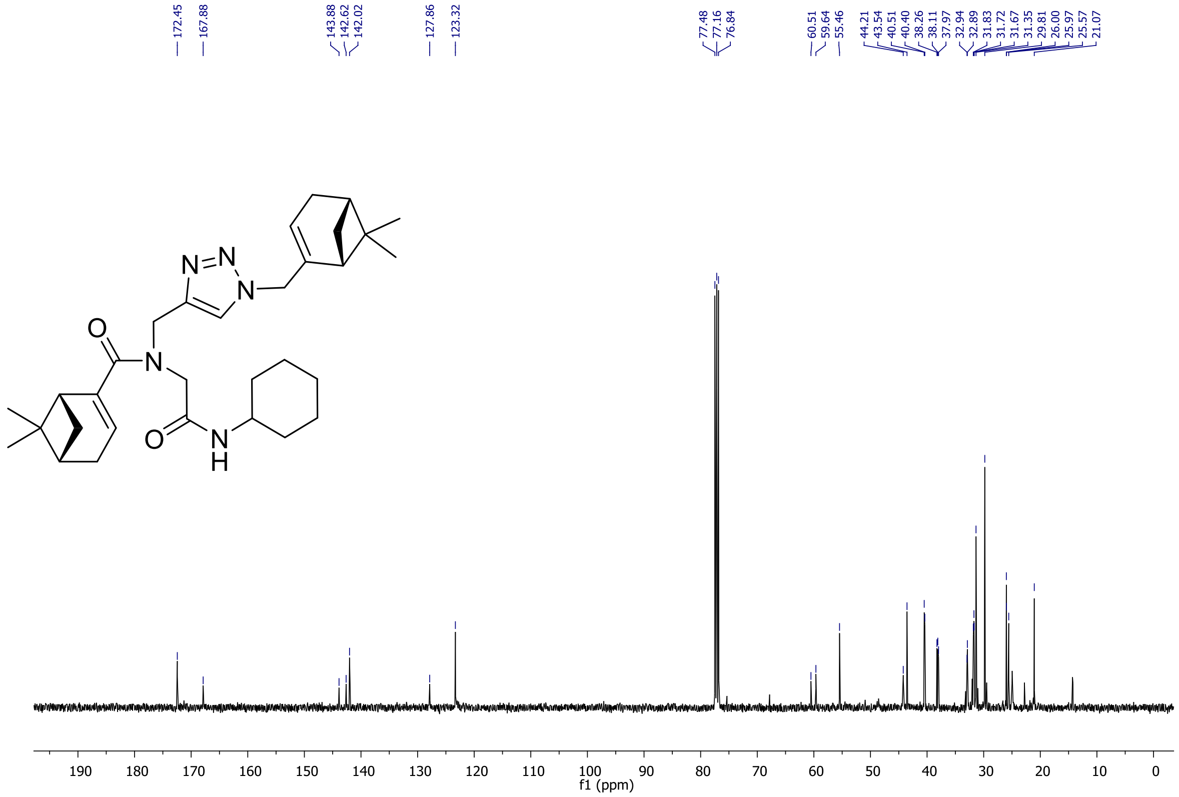

FIGURE S33: 100 MHz ^{13}C NMR spectra in $CDCl_3$ of **3d**.

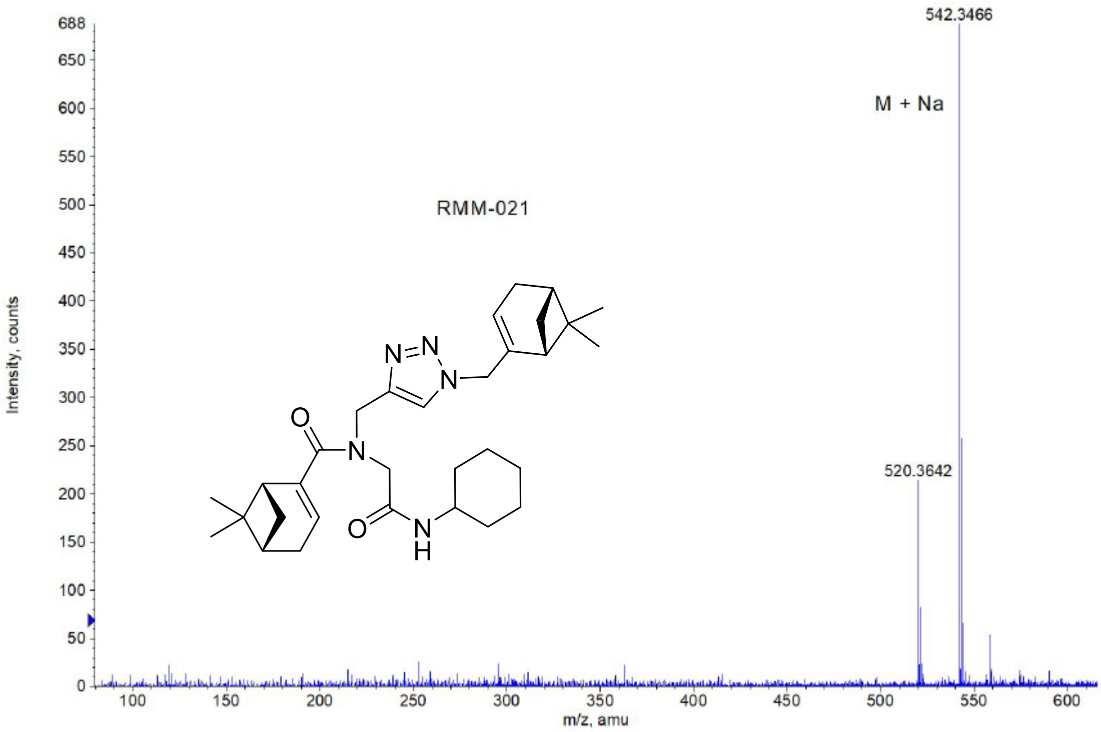

FIGURE S34. HRMS (ESI-FT-ICR) m/z spectra of **3d**.

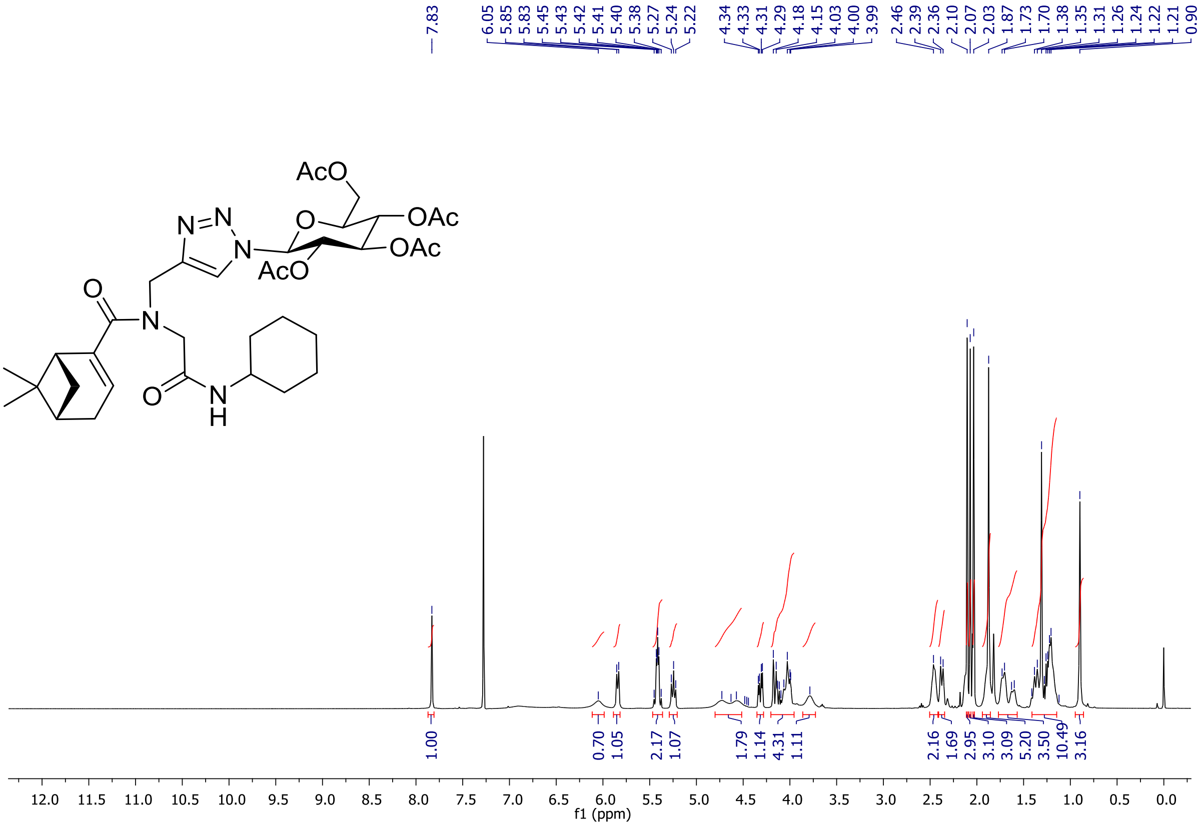

FIGURE S35. 400 MHz ^{1}H NMR spectra in $CDCl_3$ of **3e**.

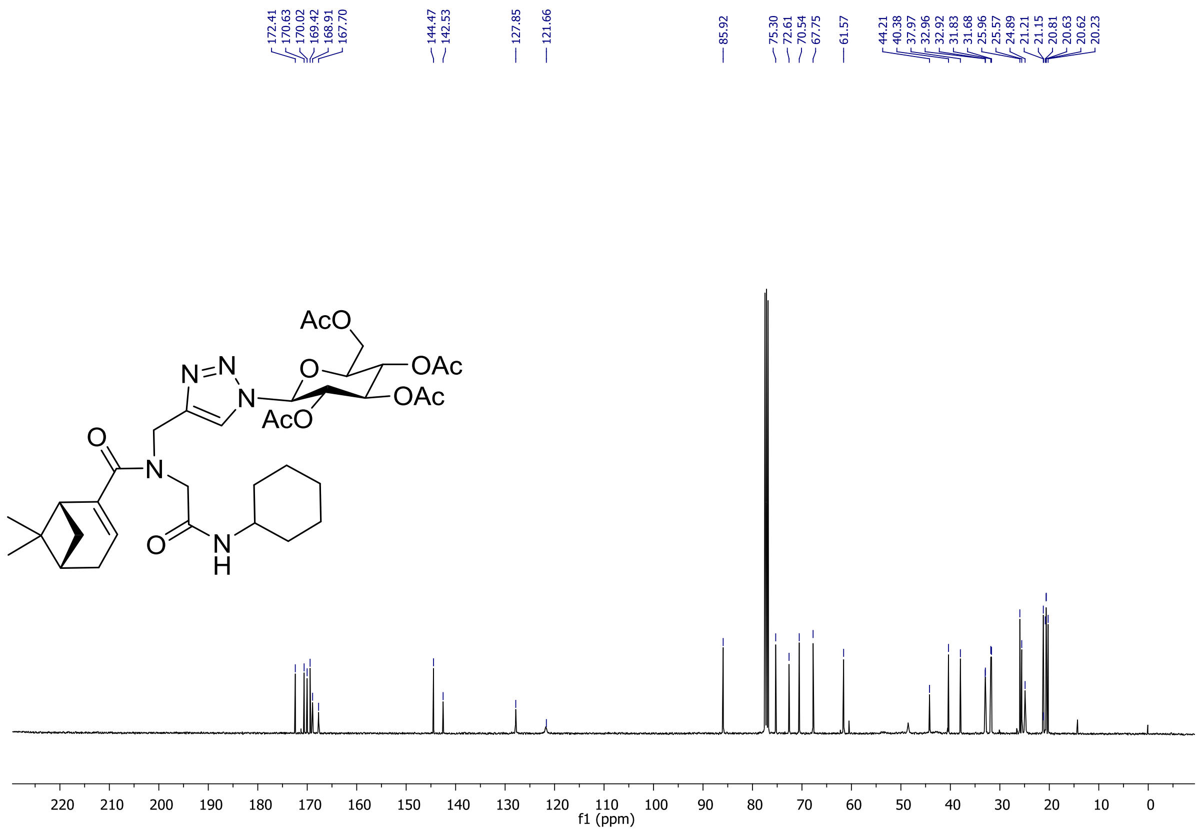

FIGURE S36. 100 MHz ^{13}C NMR spectra in $CDCl_3$ of **3e**.

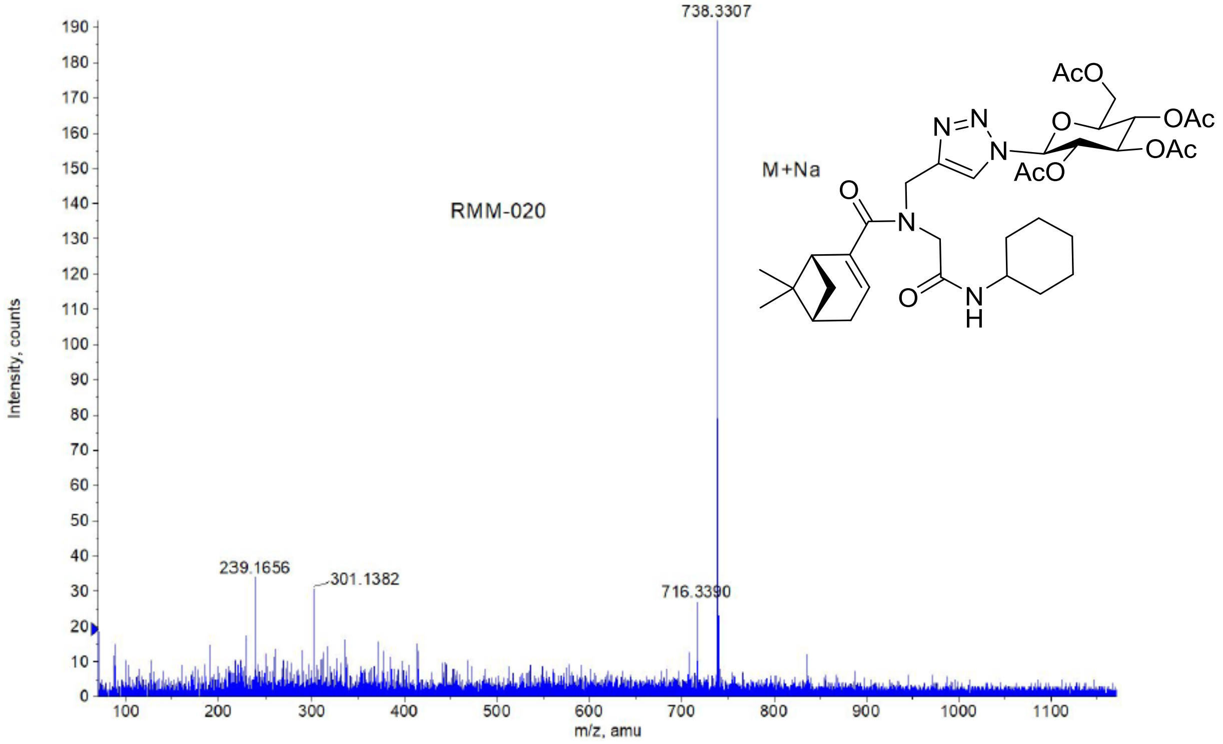

FIGURE S37. HRMS (ESI-FT-ICR) *m/z* spectra of **3e**.

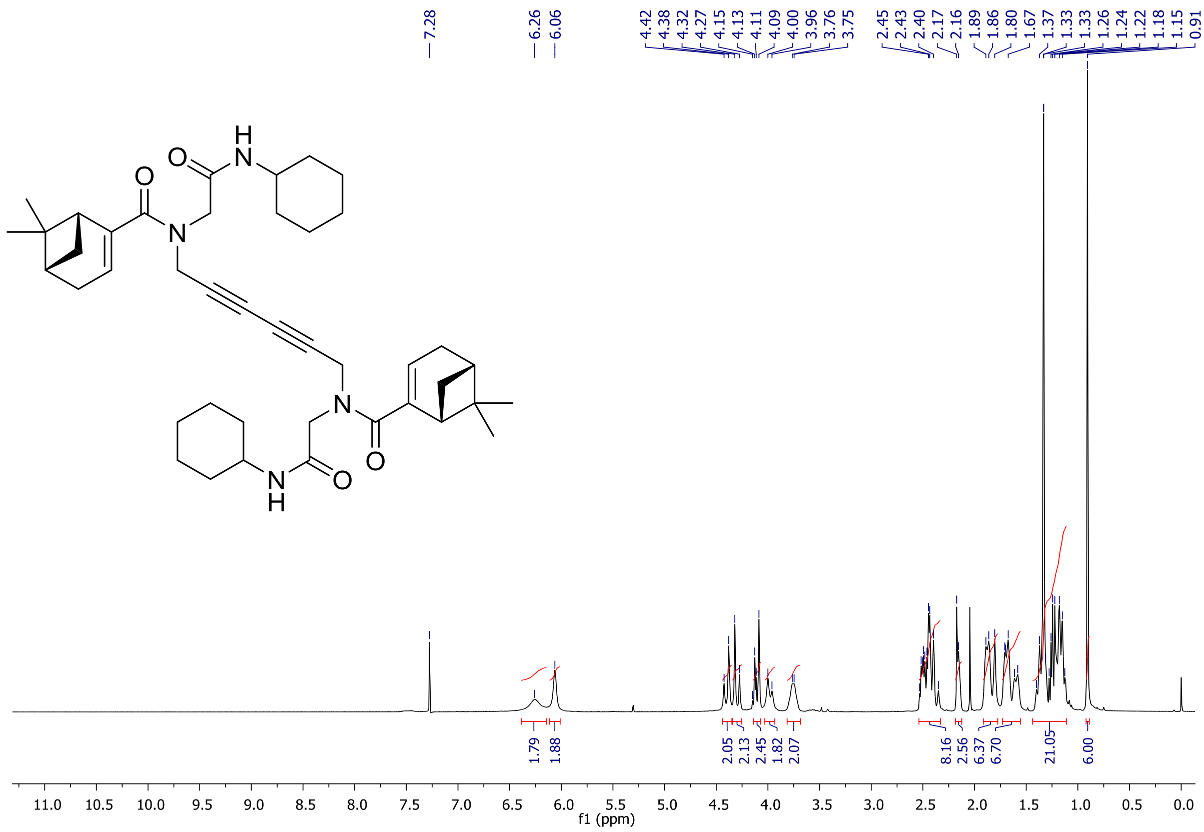

FIGURE S38. 400 MHz ^{1}H NMR spectra in $CDCl_3$ of **4a**.

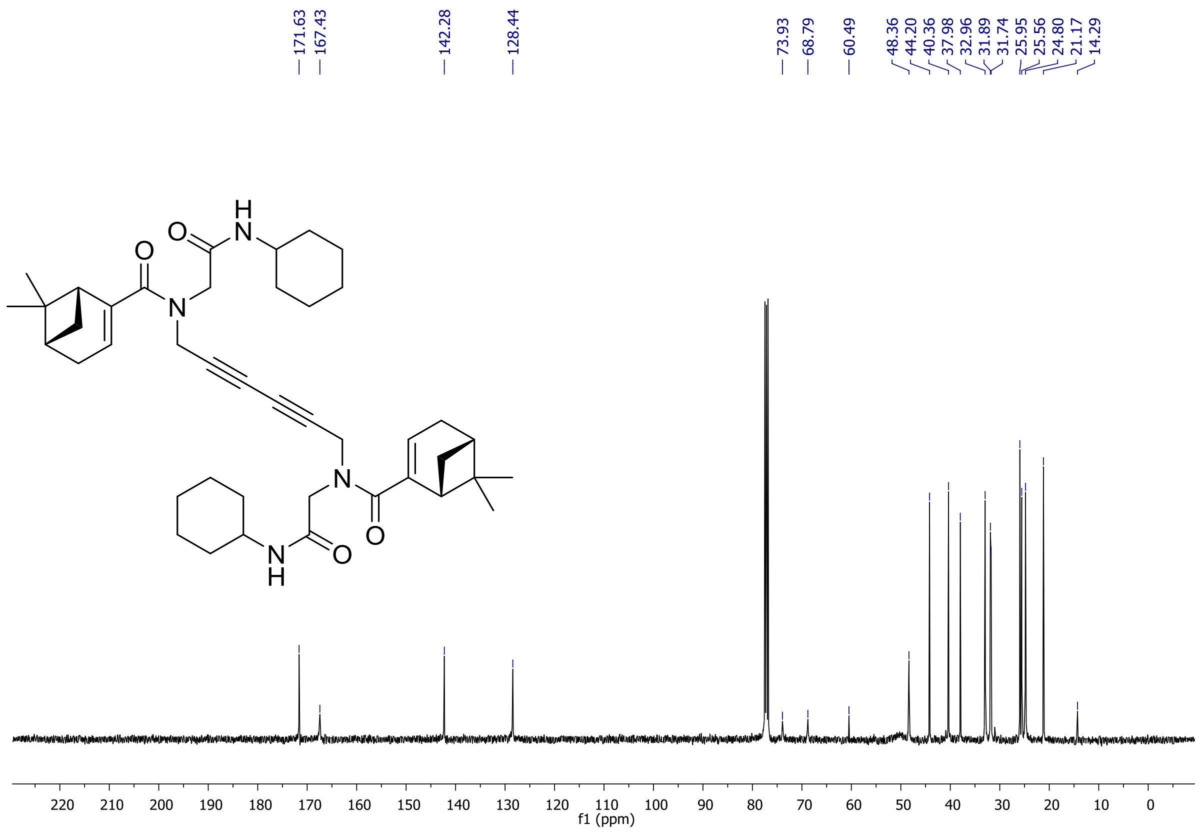

FIGURE S39. 100 MHz ^{13}C NMR spectra in $CDCl_3$ of **4a**.

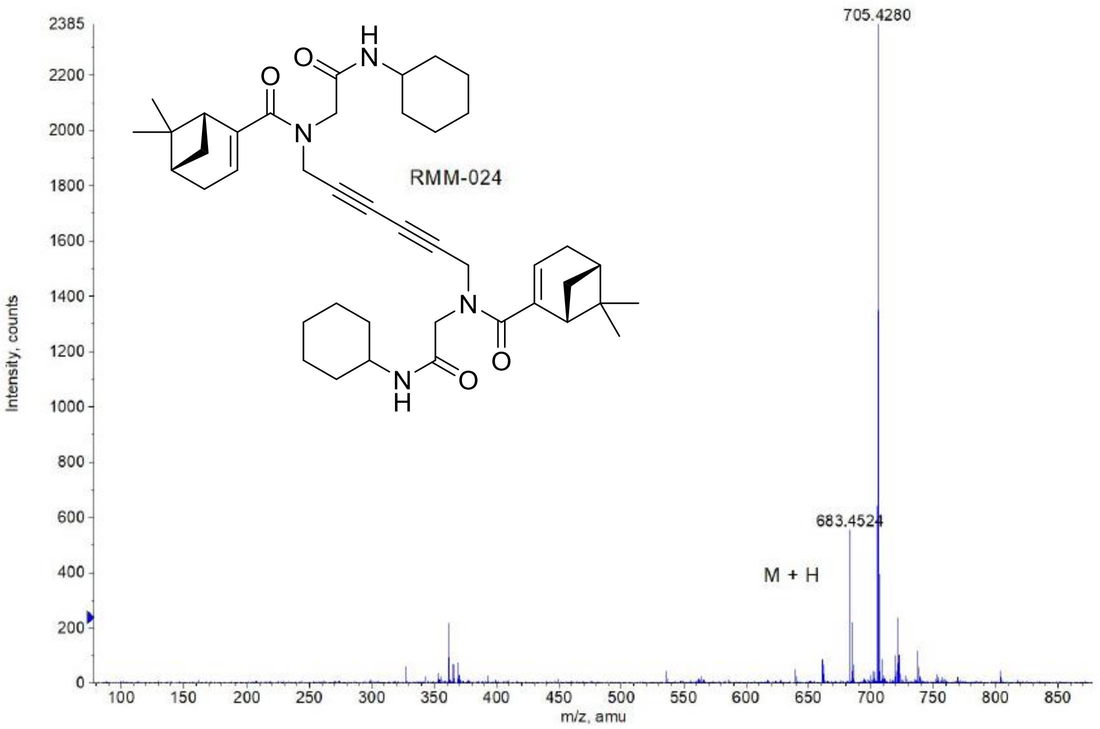

FIGURE S40. HRMS (ESI-FT-ICR) *m*/*z* spectra of **4a**.

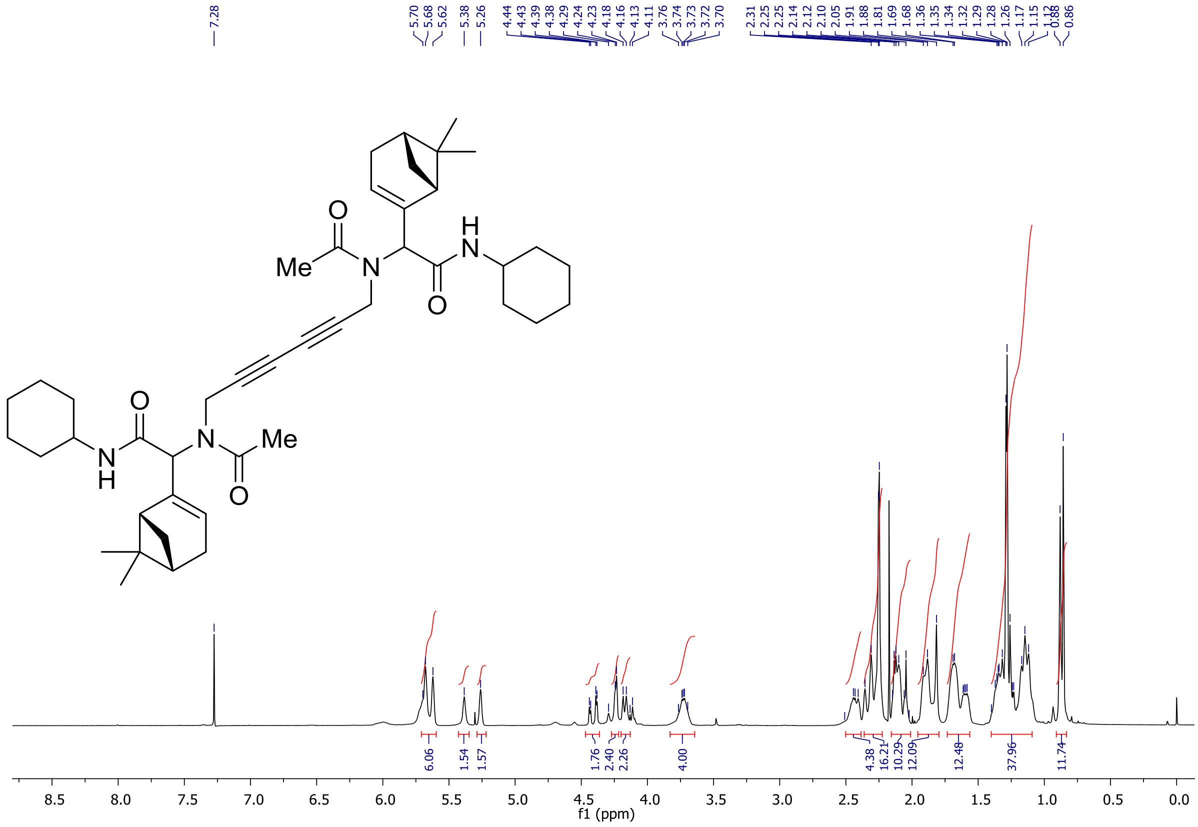

FIGURE S41. 400 MHz ^{1}H NMR spectra in $CDCl_3$ of **4b**.

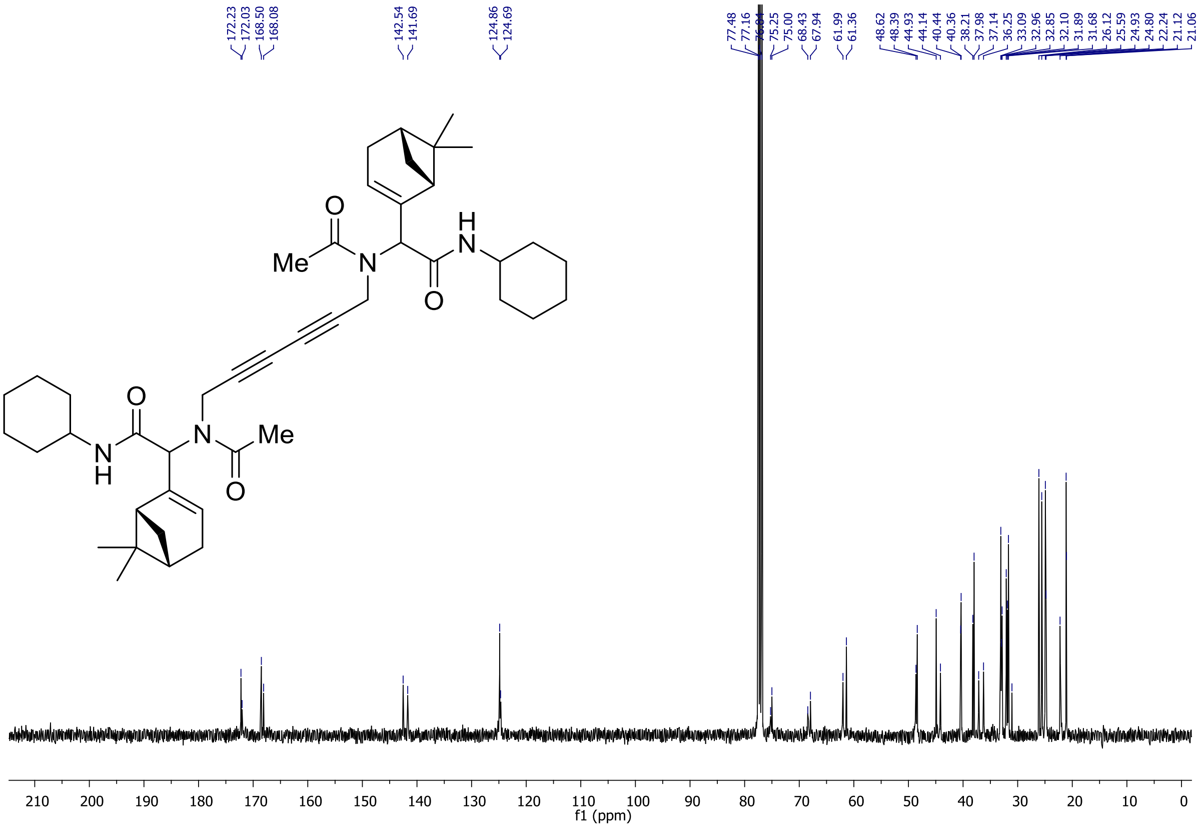

FIGURE S42. 100 MHz ^{13}C NMR spectra in $CDCl_3$ of **4b**.

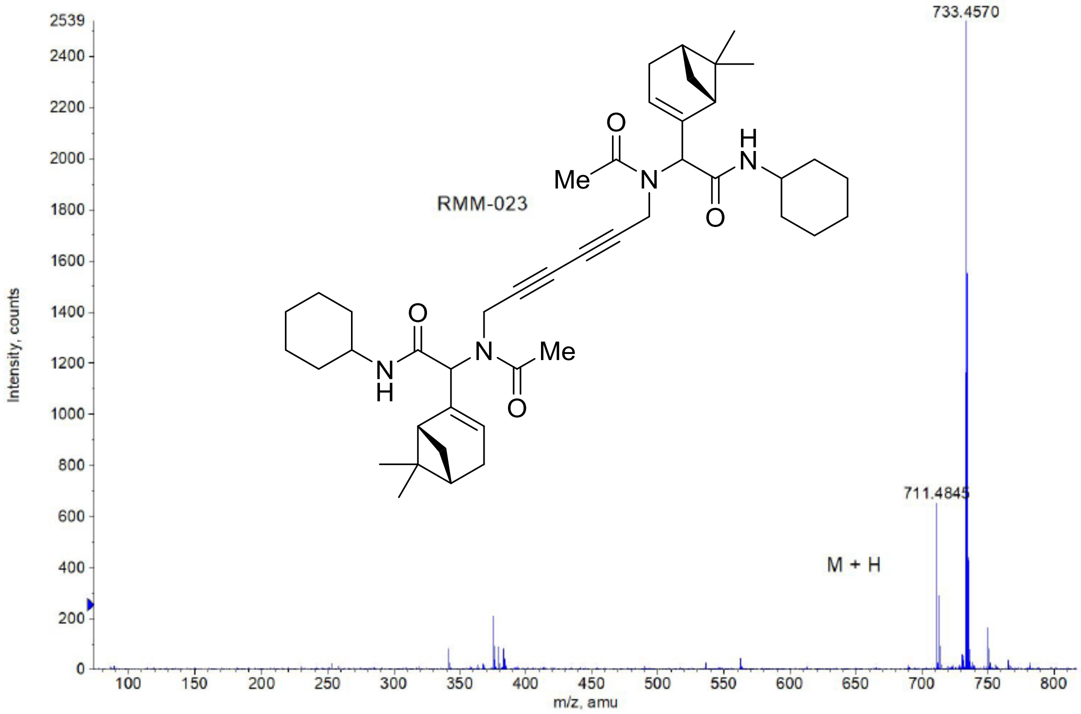

FIGURE S43. HRMS (ESI-FT-ICR) *m*/*z* spectra of **4b**.
